# Direct Observation of a Cross-Coupling Catalyst Intermediate via Single-Molecule Spectroscopy

**DOI:** 10.1021/jacs.6c09868

**Published:** 2026-09-07

**Authors:** Mackinsey A. Smith, Simi Kaur, Ralu Divan, Jeffrey Bartz, Rachel V. Czerwinski, Benjamin A. Brewster, Emily Ertl, Brian W. Hopkins, Desiree M. Bates, Angela N. Marquard, Sunil P. Upadhyay, Edwin R. Chapman, Randall H. Goldsmith

**Affiliations:** † Department of Chemistry, 5228 University of Wisconsin-Madison, Madison, Wisconsin 53706, United States; ‡ Department of Neuroscience and Howard Hughes Medical Institute, 89067 University of Wisconsin, Madison, Wisconsin 53706, United States; § Center for Nanoscale Materials, Argonne National Laboratory, Lemont, Illinois 60439, United States; ∥ Office of Information Technology, University of Mississippi, University, Mississippi 38677, United States

## Abstract

Single-molecule measurements on molecular catalysts are uniquely poised to allow observation of unsynchronized chemical dynamics and transient intermediates that are not observable using traditional bulk techniques. However, single-molecule fluorescence spectroscopy is typically performed at nanomolar concentrations, far lower than typical synthetic conditions. Here, we use nanophotonic zero-mode waveguides (ZMWs) to enable observation of the dynamics of a single catalyst under synthetically relevant (micromolar) reaction concentrations. We deploy ZMWs to perform the direct observation of a previously hypothesized intermediate of Pd-PEPPSI ( **P** yridine- **E** nhanced **P** recatalyst **P** reparation, **S** tabilization, and **I** nitiation), a well-known molecular C–C cross-coupling catalyst, by using a fluorescently labeled ligand to report on ligand binding and dissociation. Kinetic analysis of single-catalyst ligand dynamics reveals how the presence of cross-coupling reactants influences the underlying mechanism. This paradigm for single-molecule investigation of molecular catalysts at synthetically relevant concentrations is general for investigation of a wide variety of ligand and reactant dynamics.

## Graphical abstract

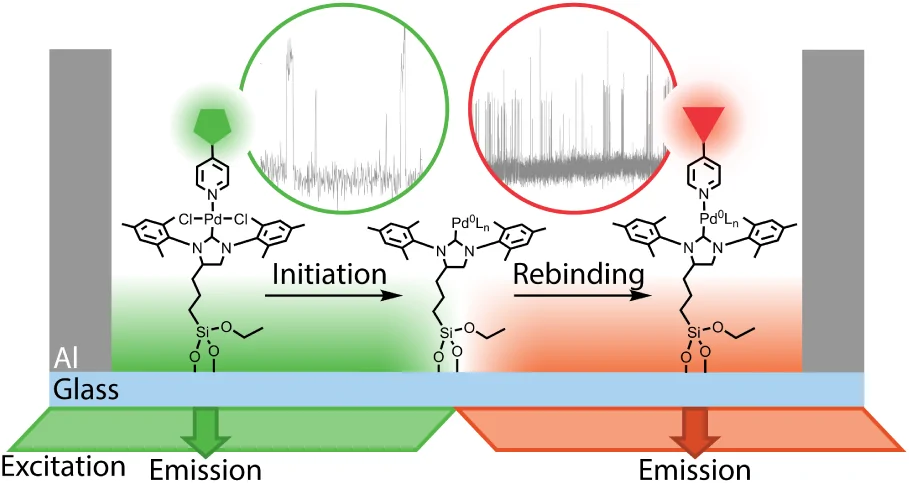

## Introduction

The extensive use of cross-couplings catalyzed by organometallic molecular catalysts has led to great interest in understanding the mechanisms and kinetics of these reactions.^1^ One popular cross-coupling catalyst is Pd-PEPPSI (Pyridine-Enhanced Precatalyst Preparation, Stabilization, and Initiation), Figure 1, which has successfully been used to catalyze Negishi,^2^ Suzuki–Miyaura,^3^ Kumada–Tamao–Corriu,^4^ and Stille couplings^5^ in high yields and a wide substrate scope. The precatalyst is a solid that is tolerant to air and moisture, making it attractive as a commercial catalyst, and the solvated species has been shown to maintain activity for over 24 h.^6^

**Figure 1:**
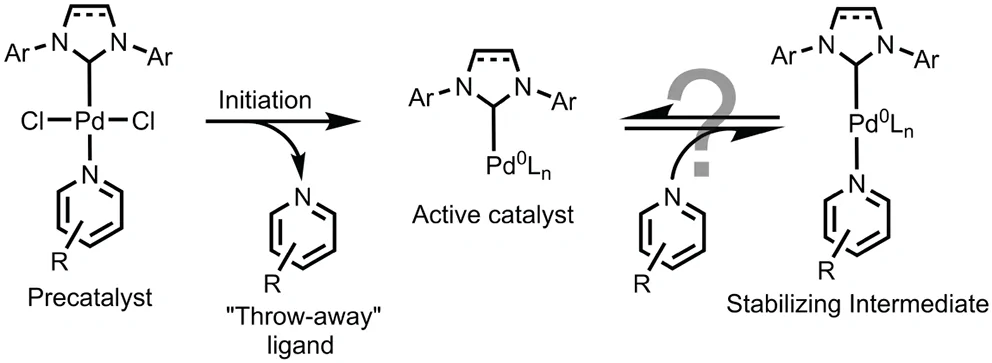
Initiation and proposed rebinding of Pd-PEPPSI-type catalysts.

A key activation step of Pd-PEPPSI is dissociation of the chloropyridine “throw-away” ligand.^3,7^ Previous studies showed that several variants with different throw-away ligands exhibited major differences in yield (changing by as much as 15%) and kinetics (with initial rates differing by an order of magnitude) implying an active role of the ligand.^6^ Additionally, the PEPPSI catalyst demonstrated a significantly higher turnover number than an in situ-generated Pd-NHC presumed to have the same active species, increasing from 7.5 to 300.^3^ The key distinction between the PEPPSI catalyst and the in situ-generated catalyst was the presence of the throw-away ligand. Taken together, these results indicate that the throw-away ligand is involved in catalytic dynamics beyond the initial initiation step. One plausible role is that the ligand transiently and repeatedly rebinds to the catalyst center during the catalytic cycle, stabilizing it and slowing catalyst decomposition (Figure 1).^6^

Although indirect evidence supports the rebinding of the pyridine ligand, there has been no direct experimental observation of the proposed rebounding intermediate. NMR has been used to study speciation of palladium catalysts before^8–10^ but only when the catalyst intermediates being detected accumulate in appreciable amounts over the course of the reaction. Low-concentration catalyst intermediates are often well beyond the detection limit of NMR.^11^ Bulk NMR studies of the PEPPSI catalyst show that 1) dissociation of the throw-away ligand only occurs in the presence of the initiator, 2) no evidence of bound pyridine ligand is observed under reaction conditions (only free, unbound pyridine ligand is detected), and 3) fluorescence accurately tracks the dissociation of the ligand in an ensemble NMR initiation experiment.^7^ These bulk results indicate that any rebound population is either small enough to be below the limit of detection of NMR and/or transient enough that it is below the temporal resolution of NMR.

More generally, there is an outstanding need for observational techniques to directly detect and characterize transient intermediates in catalysis. Because they do not require synchronization of chemical reactions, single-molecule techniques can reveal short-lived intermediates and have been deployed to observe previously hidden dynamics in the action of biological catalysts (enzymes),^12–15^ nanoparticle and heterogeneous catalysts,^16–29^ molecular organometallic catalysts,^7,30–36^ and organocatalysts and organic reactions^37–39^ (see SI, Section 5.4). Here, we report the direct observation of the rebinding of the pyridine ligand and describe a powerful methodology for the mechanistic investigation of molecular catalysts at the single-molecule level.

To utilize single-molecule fluorescence to study ligand dynamics, a relevant moiety must be labeled such that a change in the fluorescence signal (i.e., disappearance, reappearance, duration) can be attributed to the dynamics of interest. For example, by labeling the throw-away ligand, initiation can be observed via loss of fluorescence^7^ and rebinding could conceivably be observed via the reappearance of fluorescence. However, due to the need to resolve the weak single-molecule signal over a high background, single-molecule measurements are typically performed at very low fluorophore concentrations (≤10 nM).^40^ For much of the prior single-molecule work on enzymes and nanoparticle and heterogeneous catalysts where a reagent, ligand, or substrate was labeled,^15,16,18,19,21–27^ this limit was only a minor issue because such catalysts can be active in this range. However, for organometallic molecular catalysts, this nM concentration limit is generally much lower than typical reactant or ligand concentrations. Since the probability of free ligand encountering the catalyst species dramatically decreases as the ligand concentration declines, the resulting kinetics at nM concentrations will likely be nonrepresentative of the reaction at more synthetically relevant concentrations. Consequently, we require a single-molecule approach that allows access to higher concentrations.

Multiple techniques can overcome this “concentration barrier.” ^40,41^ Nanophotonic zero-mode waveguides (ZMWs) allow access to concentrations on the order of μM,^42,43,44,45^ and up to mM when combined with energy transfer,^46^ a concentration range much more representative of reaction conditions, but have thus far been deployed principally to study biological dynamics.^43–45^ Here, we describe the application of ZMWs to chemical catalysis: a method for investigation of unsynchronized behavior capable of revealing the transient, low-concentration intermediates at the heart of microscopic kinetic steps both on and off catalytic cycles. Specifically, we report direct detection of the previously unobserved short-lived intermediate formed by the rebinding of the “throw-away” ligand to the PEPPSI cross-coupling catalyst and the underlying catalyst dynamics made observable by the shifting binding affinity for the ligand.

## Results and Discussion

### Catalyst Design and ZMWs

To study the initiation and rebinding of Pd-PEPPSI using single-molecule fluorescence microscopy, the throw-away ligand must be labeled with a fluorescent dye, and the catalyst must be immobilized onto a surface for long-term imaging. A PEPPSI variant was synthesized in which the pyridine ligand is labeled with a green boron dipyrromethene (BODIPY) dye and the N-heterocyclic carbene (NHC) backbone is functionalized with a silane linker for tethering to glass, **1** (Figure 2a). BODIPY dyes were chosen due to their compatibility with metal centers (no quenching), organic solvents, and a wide range of colors and high fluorescence quantum yields.^7,36,47,48^ Surface-supported cross-coupling catalysts are of interest in their own right; immobilization of homogeneous catalysts (heterogenization) limits certain known catalyst deactivation pathways and allows for easier catalyst separation and recycling when applied to batch reaction or continuous-flow systems, while ideally maintaining the high activity, selectivity, and yields of the original molecular catalyst.^49^ To this aim, the PEPPSI catalyst system has previously been modified to include a silane linker and has been found to retain high activity and selectivity in cross-coupling reactions.^50–52^

**Figure 2:**
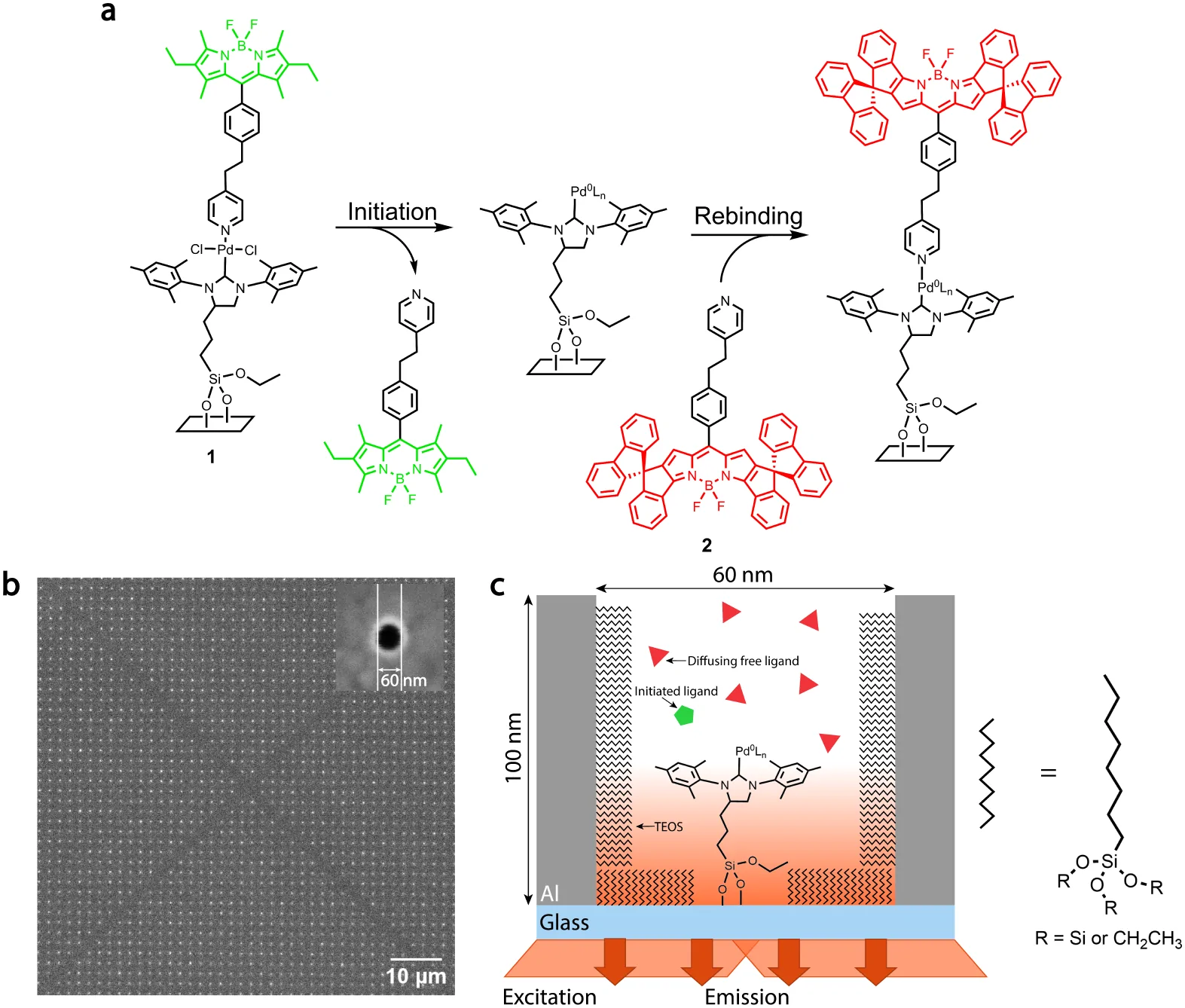
Experimental design. a) Initiation and rebinding of the tethered, labeled catalyst species. b) Representative bright-field image and scanning electron microscopy (SEM) image (inset) of a 60 nm ZMW array and well, respectively. c) Cartoon (not to scale) of the PEPPSI catalyst tethered in a ZMW well. Passivation layer (TEOS = triethoxy­(octyl)­silane) represented by zigzag lines. Initial green BODIPY label used for catalyst localization represented by a green pentagon. Free red BODIPY-labeled ligand for the rebinding study represented by red triangles.

Fluorescence from **1** allows the identification of which ZMW wells contain a single catalyst. The unbound pyridine ligand was also synthesized with a red BODIPY dye label, **2**, which, when added to the microscopy sample, allows detection of rebinding events. The use of this two-color green/red localization/rebinding scheme results in a unique signal being produced upon ligand rebinding (Figure 2a). We elected to label the throw-away ligand with a red dye because excitation with red light reduces the fluorescence background from Raman scattering of the solvent as well as fluorescence from residual impurities, which decreases substantially at higher excitation wavelengths.^53^ In particular, the highly rigid and spiro-conjugated red BODIPY **2** was selected due to its excellent photophysical properties that make it particularly well-suited to a single-molecule experiment. Specifically, **2** demonstrates a higher quantum yield compared to other red dyes, achieved via use of the rigid spiro groups, and high photostability (minimal blinking, see SI, Section 4.2),^54^ all while avoiding heteroatoms capable of substantially interfering with the catalyst.^36^

ZMWs with 60 nm-diameter wells (Figure 2b) were fabricated using electron-beam lithography^42^ on a glass substrate with aluminum walls (see SI, Section 1.4). 100–200 nm-diameter ZMWs are more typical for biological studies,^42–45^ but here, the smaller 60 nm-diameter is still much larger than the catalyst and provides better volume exclusion and lower background. To minimize nonspecific adsorption into the ZMW wells, the surface of the wells required passivation. Passivation of ZMWs with poly­(vinylphosphonic acid) (aluminum-specific passivation) and/or silanes such as polyethylene glycol silanes (nonspecific passivation) is commonly used in biological/aqueous experiments in ZMWs.^43–45,55,56^ However, these passivation protocols have never been tested for their effectiveness in blocking nonspecific adsorption of nonbiological molecules in organic solvents. A new passivation protocol using triethoxy­(octyl)­silane (TEOS) was developed that eliminated nonspecific sticking of **2** in 60 nm ZMWs up to a concentration of at least 6.5 μM (see SI, Sections 1.1 and 4.1).

### Single-Molecule Fluorescence Microscopy and Observation of Rebinding

Microscopy sample chambers were made by bonding glass tubes to the ZMW surface using a solution of sodium silicate at the interface, followed by annealing (see SI, Section 1.1).^47^ This process creates an adhesive-free microscopy chamber compatible with organic solvents. The labeled catalyst **1** was co-deposited into the ZMW chamber in isopropyl alcohol with an excess of TEOS such that multiple occupancy of **1** in wells was discouraged (Figure 2c). The sample was rinsed, refilled with degassed tetrahydrofuran (THF), with ether solvents being common for Suzuki cross-coupling reactions with Pd-PEPPSI,^57,58^ and imaged under 532 nm illumination, and green fluorescence was collected to identify the location of catalysts (Figure 3, left), as previously described (see SI, Section 1.2).^59^ During this catalyst localization step, no initiator is present to liberate the throw-away ligand, therefore, the observed changes in fluorescence intensity in the green channel are attributed to dye blinking and photobleaching and are not representative of rebinding events. Ligand rebinding was investigated under two conditions: 1) “without coupling reagents” with only the catalyst, initiator, and labeled ligand and 2) “with coupling reagents” with Suzuki coupling reagents in addition to the catalyst, initiator, and labeled ligand to more closely resemble in situ conditions. After catalyst localization, a preprepared solution was added to yield 1) a final concentration of 0.5 mM initiator (KOtBu) and 1 μM red-labeled free pyridine ligand **2** for without-coupling reagents or 2) a final concentration of 0.5 mM initiator, 1 μM red-labeled ligand **2**, and 0.5 mM each of bromobenzene and phenylboronic acid for with coupling reagents (see SI, Section 1.1). The sample was excited at 635 nm, and red fluorescence was collected for 1 h (Figure 3, right; see SI, Section 1.2). Fluorescence intensity spikes above background in the red channel are considered rebinding events.

**Figure 3:**
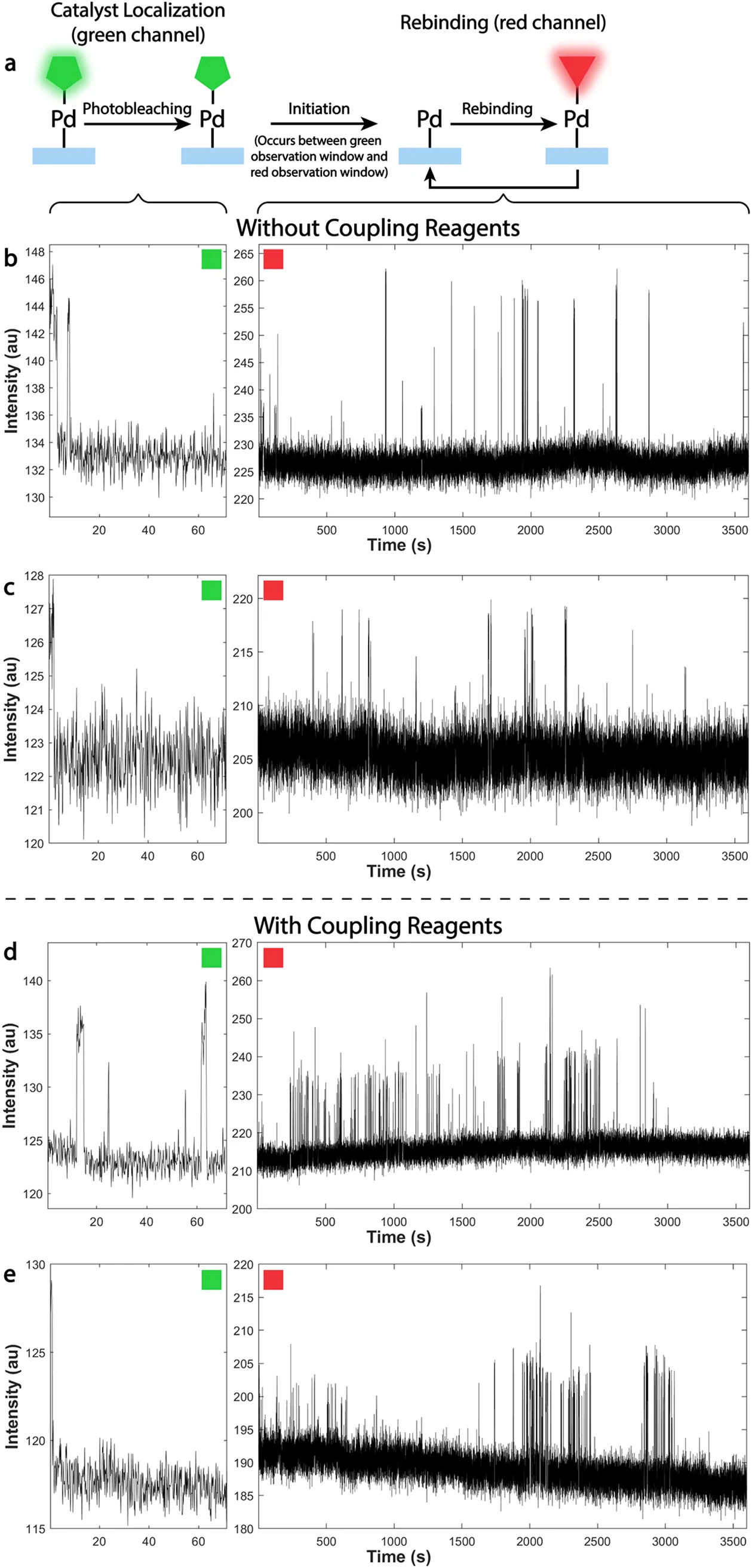
Cartoon representing the rebinding experiment (a). Representative fluorescence traces for single-catalyst molecules without coupling partners (b,c) and with coupling partners (d,e) in solution. Left: catalyst imaged before initiation with green excitation. Blinking and eventual permanent photobleaching of the dye are presumed to be responsible for the observed changes in fluorescence intensity in the green channel. Right: catalyst imaged with red excitation after initiation and introduction of 1 μM red BODIPY-labeled free ligand **2**. Spikes above background represent rebinding events. Each pair of green and red traces represents catalyst localization and rebinding data, respectively, for one single-catalyst molecule observed over the entire course of the experiment. For additional rebinding traces with and without coupling partners in solution, see SI, Sections 2.2 and 2.3. Although all molecules were investigated under the same experimental conditions, the excitation intensity experienced by each molecule will differ, resulting in differences in signal and background intensities when comparing rebinding data for different molecules (see SI, Section 5.1).

Individual catalyst molecules can be easily localized in the green channel and then monitored for rebinding activity in the red channel due to the highly ordered nature of the ZMW array (intensity traces for four individual catalyst molecules are shown in Figure 3b–e; see SI, Sections 2.2 and 2.3 for additional examples). All ZMW wells that were within the excitation area of the beam were examined for fluorescence signals above the background in both the green (catalyst localization) and red (rebinding) channels (Table 1) by simple thresholding.

**Table 1:** Summary of Single-Molecule Rebinding Data

Catalyst Observed	Rebinding Signal Observed	Total Number of Events
Without Coupling Partners		
Yes	Yes	38
Yes	No	214
No	Yes	15
No	No	8909
With Coupling Partners		
Yes	Yes	44
Yes	No	117
No	Yes	19
No	No	2970

In wells observed to contain the catalyst, 15% (without coupling partners) and 27% (with coupling partners) show red channel fluorescence signals indicating rebinding behavior (Table 1, Figure 3). Importantly, only 0.17% (without coupling partners) and 0.64% (with coupling partners) of wells without detected catalyst showed rebinding signals, and when **2** was added without the catalyst, no apparent binding signals were observed (see SI, Section 4.1), indicative of the effectiveness of the passivation and tight optical confinement of the ZMWs. It is likely that the 0.17% and 0.64% originate from rebinding to catalysts that had photobleached prior to the start of the experiment and so were not registered during the catalyst identification step. Most importantly, the significant population showing transient red signals constitutes the direct observation of the rebound PEPPSI intermediate and establishes 60 nm ZMWs as a viable platform for detecting short-lived intermediates of molecular catalysts.

Significant variability is observed in rebinding dynamics. 73–85% of catalysts show no rebinding. When rebinding is observed, the vast majority of catalysts show multiple binding events but exhibit variability in observed liganded and unliganded dwell times. The absence of rebinding activity in these catalysts does not imply a lack of cross-coupling ability or catalyst turnover. Likely, there is heterogeneity in the catalyst microenvironment such that only some can rebind the pyridine. It is also possible that there is some structural difference between the different populations of catalysts such that only some are able to rebind the pyridine. Multiple catalytically active species have been observed for other catalytic systems^60,61^ including a palladium cross-coupling catalyst.^62^ However, in this experiment, it is not possible to distinguish this heterogeneity from that of the local deposition microenvironments. It is known that chemical heterogeneity at the silica surface leads to heterogeneity in the catalyst microenvironment and accessibility within the TEOS layer.^7,63,64^

### Kinetic Analysis

To quantitatively analyze the binding dynamics, a subset of rebinding traces (n = 13 catalyst molecules without coupling partners, n = 11 catalyst molecules with coupling partners) were selected with high signal-to-noise ratios that clearly showed single-step photobleaching in the localization step, confirming that only a single catalyst was present. This subset of data was used for all kinetic analyses described hereafter. Although the use of this subset of rebinding data for kinetic analysis may introduce some selection bias, a high signal-to-noise ratio is required for the subsequent data processing.

Data were idealized using AutoDISC (Automated DIvisive Segmentation and Clustering),^65,66^ an unsupervised top-down algorithm designed to idealize noisy stepwise data, such as single-molecule intensity traces. Following idealization, a custom MATLAB code was used to extract the lengths of time the catalyst was liganded (bright, t_ons_) and unliganded (dark, t_offs_). Comparison of monoexponential and biexponential fits shows conspicuously nonmonoexponential behavior for dwell times in both liganded and unliganded states (Figure 4), indicating at least two liganded states (i.e., different chemical states with the same high fluorescence intensity level) and at least two unliganded states (i.e., different chemical states with the same low fluorescence intensity level) with different characteristic lifetimes. The possibility of overfitting precludes identifying more than two liganded or unliganded states, but it is clear that a more complicated mechanism is observed than a simple binding/unbinding equilibrium, which would produce monoexponentially distributed dwell times.

**Figure 4:**
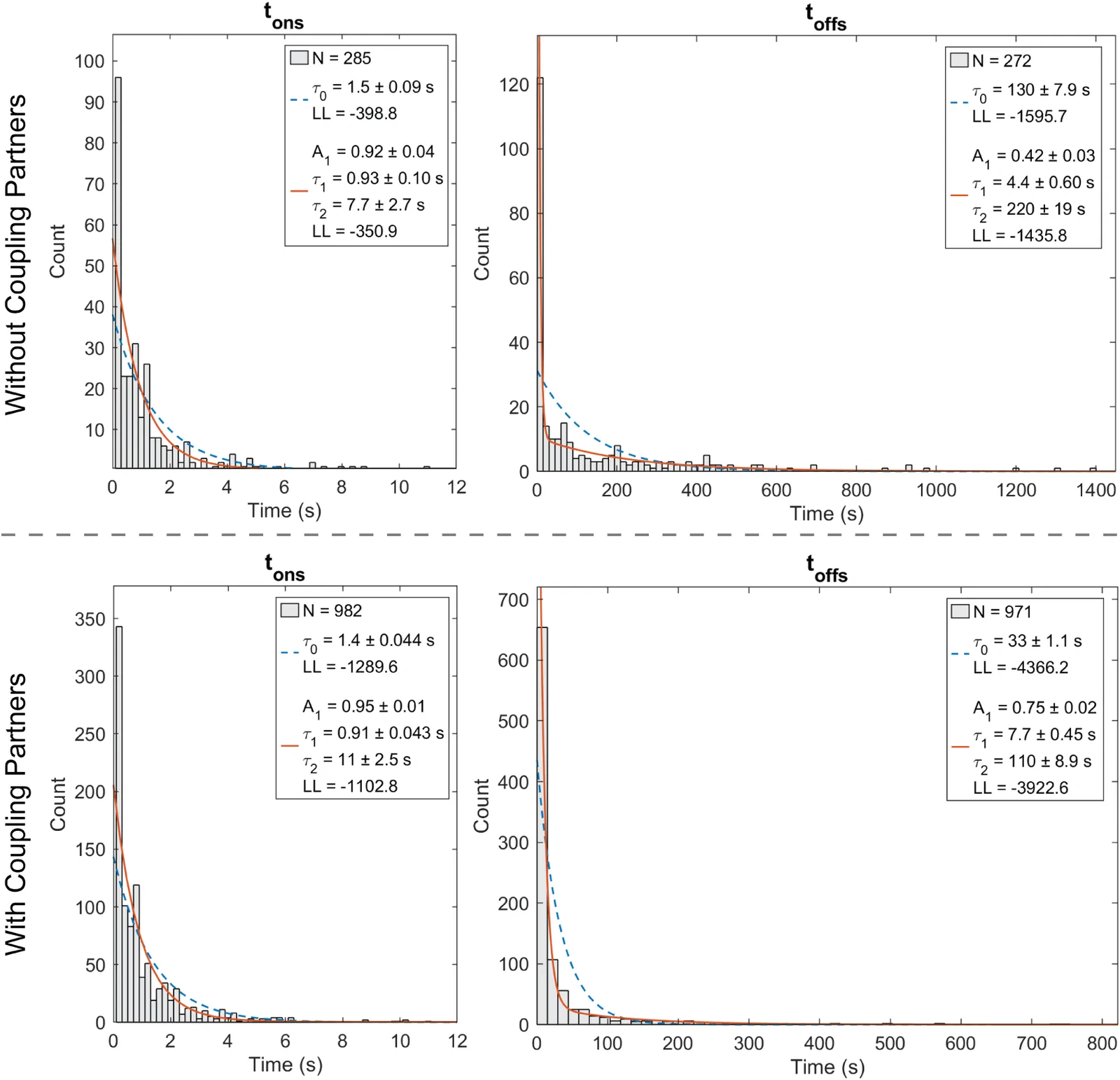
Histogram of t_ons_ (left, liganded) and t_offs_ (right, unliganded) for a subset of the single-molecule rebinding data without coupling partners (top) and with coupling partners (bottom). N is the number of events. Monoexponential fits are shown with dotted blue lines and mean τ_0_. Biexponential fits are shown in red with means τ_1_ and τ_2_ and amplitudes A_1_ and A_2_ (not listed) where A_2_ = (1 – A_1_). Log likelihood of the fit was used to determine the best fit. Less negative log-likelihood (LL) values indicate better fits. Note that *x*-axes are the same for t_ons_ and different for t_offs_ to emphasize the differing shape of the distributions.

Interestingly, rebinding traces from experiments including coupling reagents generally show a much higher density of rebinding events compared to those without coupling partners (see SI, Sections 2.2 and 2.3, for additional rebinding traces). Dwell time analysis reveals that the average lifetime of the liganded states is comparable with and without coupling partners, while the lifetime of at least one of the unliganded states is significantly shorter with coupling partners (Figure 4). This result suggests that access to the full catalytic cycle facilitates ligand rebinding, with two possible explanations: case 1: participation in the catalytic cycle deters the catalyst from entering a state where rebinding is difficult or impossible or case 2: intermediates in the catalytic cycle are able to rebind the throw-away ligand more easily than the catalyst that does not have access to the cycle. Dwell time analysis alone cannot differentiate these possibilities; therefore, we rely on additional kinetic analysis to do so.

The mechanism can be further probed via hidden Markov model^67^ analysis using the QuB (Quantifying Unknown Biophysics) software.^68^ Given single-molecule data, QuB is able to globally optimize transition rates to calculate relative model likelihood. The idealized AutoDISC data was analyzed via QuB, and all possible two-, three-, and four-state models were evaluated using the Maximum Idealized Point algorithm^69^ (see SI, Section 3.3), yielding log-likelihood (LL) values. Then, Bayesian information criterion^70^ (BIC) values, which penalize overfitting, were determined for each model. As expected from the dwell time analysis, the single-molecule data were found to be better fit by a four-state model, with two unliganded dark states and two liganded bright states. Although we cannot definitively rule out the possibility of more than two bright states and/or dark states being responsible for the observed kinetics, it is still informative to discuss the set of four-state models and the possible underlying mechanisms under the assumption that only two bright states and two dark states are responsible for the observed kinetics. We will focus on the scheme in Figure 5, as this scheme gave the most favorable BIC values for both datasets among all considered models (see SI, Section 3.3). Simulation of this scheme results in dwell time distributions qualitatively similar to Figure 4 (see SI, Section 3.4).

**Figure 5:**
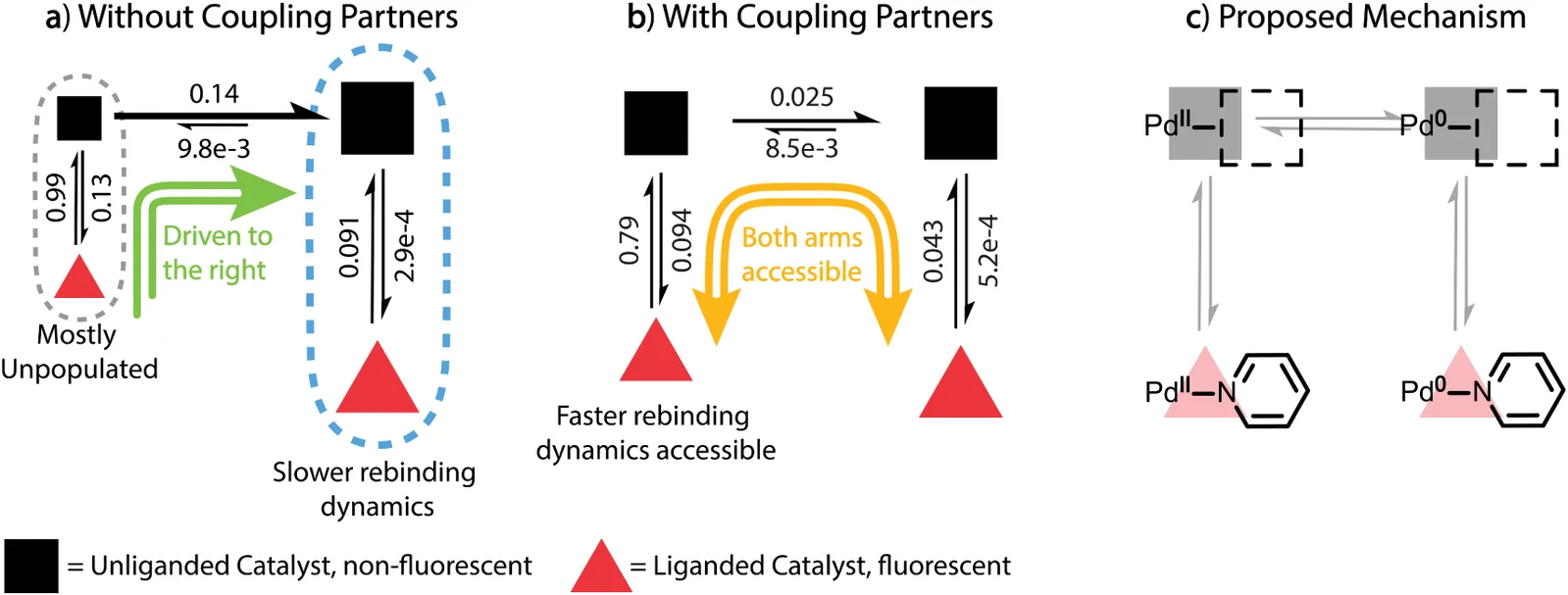
a,b) Visualization of how the addition of coupling partners changes the equilibrium between unligated (dark) states, which allows access to the faster “left arm” of the model. Each arm is qualitatively scaled to illustrate the change in the relative populations as a result of the changing equilibrium between unligated species. Transitions between states are labeled with their respective rates (in s^–1^) calculated by QuB; see SI, Figure S6 for standard errors. Black squares represent unligated (dark) states and red triangles represent ligated (bright) states. c) Illustration of a plausible mechanism for the observed bright and dark states in which each ligated/unligated pair (i.e., each “arm” of the model) corresponds to Pd­(II) or Pd(0).

The addition of coupling reagents does not change the favored model but does alter the rates in mechanistically interesting ways. The rates associated with the left and right “arms” of the scheme do not change significantly upon the addition of the coupling reagents (Figure 5a and b). In contrast, kinetics associated with interconversion between unliganded states (center equilibrium) change substantially, with one rate staying the same while the other drops by nearly an order of magnitude. Consequently, the equilibrium constant associated with this process shifts from a “product” favoring K_eq_ = 14 for the scheme with no coupling reagents to a more equally distributed K_eq_ = 2.9 when coupling reagents are added. Thus, even as rates associated with the ligand association and dissociation remain mostly unchanged, the left arm is largely inactive without the coupling reagents due to the higher equilibrium constant of the unliganded (central) state interconversion, while both arms contribute more equally to the kinetics when coupling reagents are added. Since the left arm is characterized by substantially faster binding rates than the right arm, altering the unliganded state interconversion equilibrium constant results in dramatically lowered unliganded state dwell times (t_offs_), as observed in Figure 4.

Thus, Figure 5 suggests that case 1 is operative. In case 1, the addition of coupling reagents discourages states where rebinding is difficult or impossible, consistent with the shifted equilibrium constant of the unliganded state interconversion and the resulting deemphasis of the slow ligand binding in the right arm. The other possibility, case 2, required the addition of the coupling reagents to open access to new states with higher binding rates. This possibility would have manifested as a kinetic scheme with a binding step with a substantially elevated rate that was not observed.

Given the modifications to the native catalyst species, we do not expect extracted kinetic parameters to exactly map onto those of the unmodified system (see the SI, Section 5.2). However, previous studies of rates of initiation of labeled, surface-supported catalysts in ZMWs showed good agreement with bulk solution-phase values,^7^ suggesting that our model system provides a reasonable approximation for solution-phase behavior in the unmodified system. Considering the inability of bulk measurements of unmodified catalysts to even detect the transient species of interest, the single-molecule kinetic analysis presented here provides a substantial new mechanistic perspective.

### Mechanistic Interpretation of the Kinetic Scheme

The discussion above showcases the ability of single-molecule experiments to capture unsynchronized reaction dynamics. On the other hand, single-molecule experiments, which typically lack the chemical specificity of, say, NMR or vibrational spectroscopy, generally rely on additional information to identify the observed states. Although direct experimental determination of the chemical structures responsible for the observed bright and dark states is beyond the scope of this paper, and thus, we cannot definitively assign molecular states to the kinetic scheme in Figure 5, here, we offer a plausible mechanistic interpretation consistent with the data, after ruling out contributions from dye blinking (see SI, Section 4.2) and static heterogeneity (see SI, Section 3.2).

Focusing first on conditions without coupling reagents, our kinetic scheme is consistent with equating each liganded/unliganded pair with a Pd(0) or Pd­(II) species, respectively (Figure 5c). In this interpretation, the left “arm” is associated with the Pd­(II) species since the relatively hard Pd­(II) species is expected to bind the borderline hard throw-away ligand more strongly than the very soft Pd(0) species. This expectation is corroborated by DFT calculations, which show the dissociation energy of 3-chloropyridine from Pd­(II) is 5 kcal mol^–1^ higher than from Pd(0).^3^ Thus, with the liganded species as the product, the left “arm” has K_eq_ = 0.13 while the right “arm” has K_eq_ = 3.2 × 10^–3^. A variety of oxidation rates have been reported for palladium complexes, ranging from diffusion-limited rates for electron-rich phosphine-ligated or bis-NHC-functionalized species^71,72^ to NHC-Pd catalysts capable of performing under aerobic conditions,^73^ suggesting substantially slower rates of reaction with O_2_. The measured ∼100 s lifetime of the proposed Pd(0) species (Figure 5a,b) is consistent with this broad range, especially considering that here, experiments were performed in unmixed, degassed THF dispensed and used in open air (see SI, Section 1.1), conditions where oxygen saturation is expected to take many hours.^74^ In our kinetic scheme, reformation of the Pd(0) species from the Pd­(II) species has a lifetime of ∼7 s (Figure 5a,b). We previously measured initiation of the precatalyst,^7^ which includes formation of the Pd(0) species from Pd­(II) followed by ligand loss,^6^ to take 10s of seconds. Reduction of the palladium complex unliganded with the pyridine ligand, and thus likely less electron-rich, is expected to be somewhat faster than initiation of the precatalyst, as seen. Thus, the measured rates are consistent with these state assignments.

The inclusion of coupling reagents adds the catalytic cycle as an additional way to cycle between the Pd(0) and Pd­(II) species. After oxidative addition, the newly formed Pd­(II) species must first undergo transmetalation, followed by reductive elimination to regenerate the Pd(0) species. The presence of two sequential steps will serve as a bottleneck for the Pd­(II) species,^12^ leading to a decrease in the apparent rate of consumption relative to Figure 5a; thus, the presence of the catalytic cycle is observed as an increase in the lifetime of the Pd­(II) species. Trends in the reverse process, the conversion of Pd(0) to Pd­(II), are more difficult to rationalize. Here, the rates of consumption of Pd(0) via oxidation by O_2_ and oxidative addition by the aryl bromide species should be additive, resulting in a faster observed transition rate. Instead, the rate is largely unchanged upon addition of the coupling reagents, suggesting that another unidentified process may be masking the effect. The unligated (central) equilibrium has an equilibrium constant K_eq_ = 2.9, suggesting that the Pd(0) species is the catalyst resting state, as is often encountered in Suzuki couplings.^75^ An alternate mechanistic interpretation focused on surface dynamics of the catalyst is discussed in the SI (Section 5.3) and deemed less plausible.

Although we have presented a plausible mechanism consistent with our experimental observations, structural determination of the exact chemical species responsible has not been achieved. However, substantial insight into the nature of the catalyst species can be provided by studying the dependence of rebinding dynamics on ligand or substrate concentration. For example, repeating these rebinding studies while systematically varying the concentration of the labeled ligand **2** and/or adding unlabeled throw-away ligand to the microscopy solution would more concretely demonstrate which steps of the model are dependent on ligand concentration. Similarly, investigation of how the measured kinetics change with the concentration of the coupling partners would reveal the dependence of specific steps of the model on the coupling partner concentration. The coupling partners could also be varied to determine how sensitive the ligand rebinding is to changes in the substrate. This strategy can also be combined with fluorogenic readouts^15,16,18,19,21–29,40,41,76^ to provide direct information about turnover. We emphasize that our method is a highly informative readout that can be combined with many typical ways of exploring the catalyst mechanism.

## Conclusions

Single-molecule microscopy using zero-mode waveguides allowed direct observation of the dissociation and association of the pyridine “throw-away” ligand to Pd-PEPPSI to form a previously only hypothesized intermediate. That intermediate was detected via changes in fluorescence intensity in a two-color labeling scheme at synthetically relevant μM concentrations of the fluorescent species. The liganded and unliganded dwell times were extracted and show conspicuously nonmonoexponential behavior, indicating a more complicated mechanism than a simple association/dissociation involving at least two liganded states and at least two unliganded states. Hidden Markov Model analysis demonstrated that, in the presence of coupling agents, the equilibrium between the unliganded species shifts to favor the species with faster ligand binding, resulting in a higher density of observed rebinding events than in experiments without coupling partners. Pd(0) and Pd­(II) were identified as candidates for the observed states consistent with the dynamics.

Importantly, this study shows how powerful ZMWs can be in the study of a nonbiological molecular catalyst. Beyond providing direct confirmation of a previously hypothesized intermediate, our approach provides a blueprint for using single-molecule microscopy at synthetically relevant concentrations of chemical reagents to observe transient species and provide mechanistic insight and can be deployed with a variety of ligands. Further, the use of labeled ligands to monitor the shifting coordination environment of a molecular catalyst offers a fundamentally different operational capacity for examining molecular catalysis. In future work, reaction schemes could be investigated akin to traditional kinetic studies—varying reactant concentration, reactant or catalyst structure, or temperature—but at the single-molecule level, where kinetics, equilibria, and chronology of transient species can all be simultaneously extracted under synthetically relevant concentrations.

## Supplementary Material



## Notes

Methods, supplemental figures, supplemental analysis, control experiments, supplemental discussion, and supplemental synthetic protocols and characterization (PDF)

## Abbreviations

AutoDISC: Automated DIvisive Segmentation and Clustering
BIC: Bayesian Information Criterion
BODIPY: Boron dipyrromethene
LL: Log Likelihood
NHC: N-Heterocyclic Carbene
PEPPSI: Pyridine-Enhanced Precatalyst Preparation, Stabilization, and Initiation
QuB: Quantifying Unknown Biophysics
SEM: Scanning Electron Microscopy
TEOS: Triethoxy­(octyl)­silane
THF: Tetrahydrofuran
ZMW: Zero-Mode Waveguide
