# Supplementary material for: Direct Observation of a Cross-Coupling Catalyst Intermediate via Single-Molecule Spectroscopy
Source: J Am Chem Soc. 2026 Sep 7;148(37):39751–61. doi: 10.1021/jacs.6c09868 (PMC13614192; doi:10.1021/jacs.6c09868)
Supplement: Supplementary file 1 [file ja6c09868_si_001.pdf]

Supporting Information for

Direct Observation of a Cross-Coupling Catalyst Intermediate via Single-Molecule Spectroscopy

*Mackinsey A. Smith[†], Simi Kaur[†,‡], Ralu Divan[§], Jeffrey Bartz[†], Rachel V. Czerwinski[†], Benjamin A. Brewster[†], Emily Ertl[†], Brian W. Hopkins[¶], Desiree M. Bates[†], Angela N. Marquard[†], Sunil P. Upadhyay[†], Edwin R. Chapman[‡], Randall H. Goldsmith[†]**

[†] Department of Chemistry, University of Wisconsin-Madison, Madison, WI, 53706, United States

[‡] Department of Neuroscience and Howard Hughes Medical Institute, University of Wisconsin, Madison, WI, 53706, United States

[§] Center for Nanoscale Materials, Argonne National Laboratory, Lemont, IL, 60439, United States

[¶] Office of Information Technology, University of Mississippi, University, Mississippi 38677, United States

Table of Contents

1. Methods S3
   1.1 Microscopy Sample Preparation S3
   1.2 Fluorescence Microscopy S5
   1.3 Data Analysis S6
   1.4 ZMW Fabrication S7
2. Supplemental Figures S8
   2.1 Beer's Law Plot Used to Calculate Concentration of **2** S8
   2.2 Additional Examples of Rebinding Traces Without Coupling Partners S9
   2.3 Additional Examples of Rebinding Traces With Coupling Partners S10
   2.4 Standard Errors of Rates in Scheme from Figure 5 ..... S11
3. Supplemental Analysis S11
   3.1 Effect of the BODIPY Label on Ligand Dynamics S11
   3.2 Data Distribution by ROI S13
   3.3 Model Selection for Mechanistic Discussion S22
   3.4 Simulated Dwell Time Distribution S25
4. Control Experiments S26
   4.1 Controls for nonspecific adsorption S26
   4.2 Blinking Control Experiment S29
5. Supplemental Discussion S32
   5.1 Differences in Observed Intensities S32
   5.2 Effect of Confinement and Passivation on Kinetic Results S32
   5.3 Alternative Identification of States S33
   5.4 Summary of some important prior work in single-molecule catalysis S34
6. Supplemental Synthetic Protocols and Characterization S35
7. Supplemental References S49

1. Methods

1.1 Microscopy Sample Preparation

All glassware used for single-molecule experiments was rinsed with HPLC grade methanol (Fisher), Millipore water, then given a final rinse with HPLC methanol from a cleaned glass bottle before being exposed to ambient oxygen plasma (Harrick Plasma PDC-001-HP, 45W) for at least five minutes to remove any remaining organic impurities. ZMWs were only exposed to plasma for five minutes to prevent additional etching from occurring. SEM imaging shows no change to the well sizes and no evidence of additional defects after five minutes of plasma exposure. HPLC grade THF (Fisher) dispensed from a Pure Process Technology solvent purification system and HPLC grade IPA (Fisher) was found to be sufficiently absent of fluorescent impurities in single-molecule ZMW experiments without further purification.

ZMW microscopy chambers were prepared (Figure S1).[1] Glass tubes (OD: 13 mm, ID: 10 mm, borosilicate, 33 COE) were cut to the desired length using a diamond circular grinder and the end was polished on a 500-grit silicon carbide disk. Both the tube and the ZMW were cleaned as described above. A solution of 40% sodium silicate (40° Baumé, J. T. Baker) in Millipore water was made and the glass tube was dipped into the solution, then tapped 2-3 times on clean aluminum foil to remove excess. The glass tube was placed onto the ZMW, using the registration cross and arrow to ensure all arrays were within the chamber, and weighed down using a ball bearing. The glass tube was twisted one quarter turn on the ZMW surface to ensure a good seal, then the sodium silicate solution was cured by heating the chamber to 75°C on a hot plate. The temperature was increased by 15°C every fifteen minutes until a final temperature of 135°C was reached and maintained for 16 hours. The chambers were allowed to cool to room temperature before use. This procedure generates adhesive-free ZMW microscopy chambers that are compatible with organic solvents.

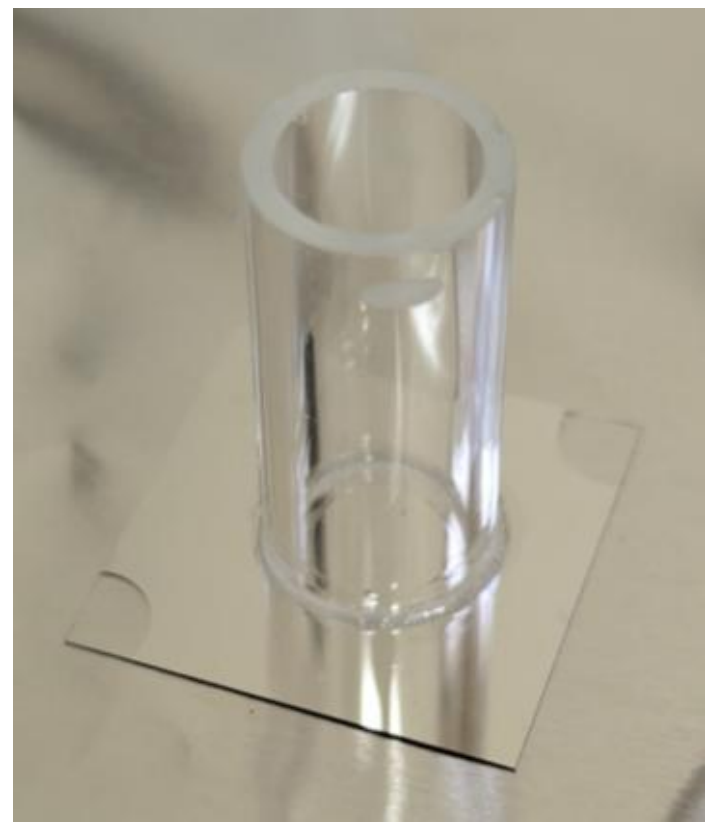

Figure S1. ZMW Chamber.

New solutions were made for each experiment. All deposition, cleaning, and microscopy solutions were added to the ZMW chamber via use of an appropriately sized micropipette. A solution of 20% V/V triethoxy(octyl)silane in IPA was made and used to create a dilute solution of **1** for ZMW single-molecule deposition (~µM in catalyst). A 4 mM solution of KOtBu in THF was sonicated for ~2 hours to fully dissolve. The ZMW chamber was cleaned as described above and 200 µL of the triethoxy(octyl)silane + **1** solution was added to the chamber and allowed to physisorb, covered, for one hour with light agitation via a mini blot mixer, then for an additional hour without agitation. The solution was removed and the ZMW was dried with nitrogen before being heated at 110°C for 10 minutes to drive off water and covalently bind the passivation layer and catalyst to the surface. Before imaging, the chambers were rinsed out twice with 900 µL of HPLC grade IPA, twice with 900 µL of clean THF, then dried with nitrogen. A solution of **2** was made in THF and the concentration was determined by UV-vis. Just before the microscopy experiment a solution of 1 mM base and 2 µM **2** in THF was made for rebinding experiments without coupling partners. For rebinding experiments including Suzuki coupling reagents, major fluorescent impurities were removed via the cleaning procedures described in the next paragraph, and a solution of 1 mM base, 2 µM **2**, 1 mM bromobenzene, and 1 mM phenylboronic acid in THF was made.

Prior to use, bromobenzene was added to a clean vial and left in a 2.5”x2.5”x4.5” aluminum box containing 18 high power LEDs (Luxeon, 700 mA, 450 nm) for 8 hours twice and stored at -20°C.

Phenylboronic acid was dissolved in minimal hot clean THF and then recrystallized in Millipore water. The crystals were isolated using gravity filtration and dried briefly under reduced pressure before being stored at 5°C under a nitrogen atmosphere before use.

To minimize drift in the xy plane, the sample was taped to the microscope stage. 100 µL of clean THF was added to the chamber and the ZMW was first imaged under 532 nm illumination to localize catalysts. Then, 100 µL of the 1 mM base and 2 µM **2** solution or 100 µL of the 1 mM base, 2 µM **2**, 1 mM bromobenzene, and 1 mM phenylboronic acid solution was added to yield a final imaging solution of 0.5 mM base and 1 µM **2** or 0.5 mM base, 1 µM **2**, and 0.5 mM of each coupling reagent, respectively, and the ZMW was imaged under 635 nm illumination for one hour. To minimize evaporation of the reaction solution over the course of the experiment, a coverslip was placed on top of the chamber.

1.2 Fluorescence Microscopy

A modified inverted microscope (Nikon Eclipse Ti-E) was used for single-molecule fluorescence experiments.[2] Excitation was provided using a 200 mW 532 nm laser (Lasertack) or a 140 mW 638 nm laser (Vortran, measured wavelength 635 nm). Each laser was attenuated with an ND filter (13.6 mW at back of microscope for green, 13.0 mW at back of microscope for red), then the beam size was expanded 20x (532 nm) or 14x (638 nm) to be somewhat larger than the back aperture of the objective (Nikon, Apo TIRF, 60x, NA 1.49, oil). The beams were coaligned using a 556 short-pass dichroic (Semrock, FF556-SDi01-25x36). A 405/488/532/635 nm multi-edge dichroic (Semrock, Di03-R405/488/532/635-t1) was used to direct the excitation light to the sample and direct fluorescence to the detection setup. During excitation with 532 nm light, a 532 nm long-pass emission filter (Semrock, BLP01-532R-25) and a 562/40 nm band-pass emission filter (Semrock, FF01-562/40-25) were used. During excitation with 635 nm light, a 635 nm long-pass emission filter was used (Semrock, BLP01-635R-25). Emission was recorded on an sCMOS camera (Andor, SONA-4BV11). The sCMOS was cooled to -45°C during data acquisition. Data acquisition was done

using Andor Solis software (532 nm illumination, catalyst localization) and a custom written LabView code (635 nm illumination, rebinding imaging). Fluorescence recordings were taken with an exposure of 100 ms and a frame rate of 7 fps. Brightfield images were taken with an exposure of 500 ms. The LabView code was written to take five minutes of fluorescence data before taking a brightfield image and repeating this for an hour. The brightfield images were used as registration images to correct for any observed xy drift. The Nikon Perfect Focus System was used to maintain focus in the optical axis.

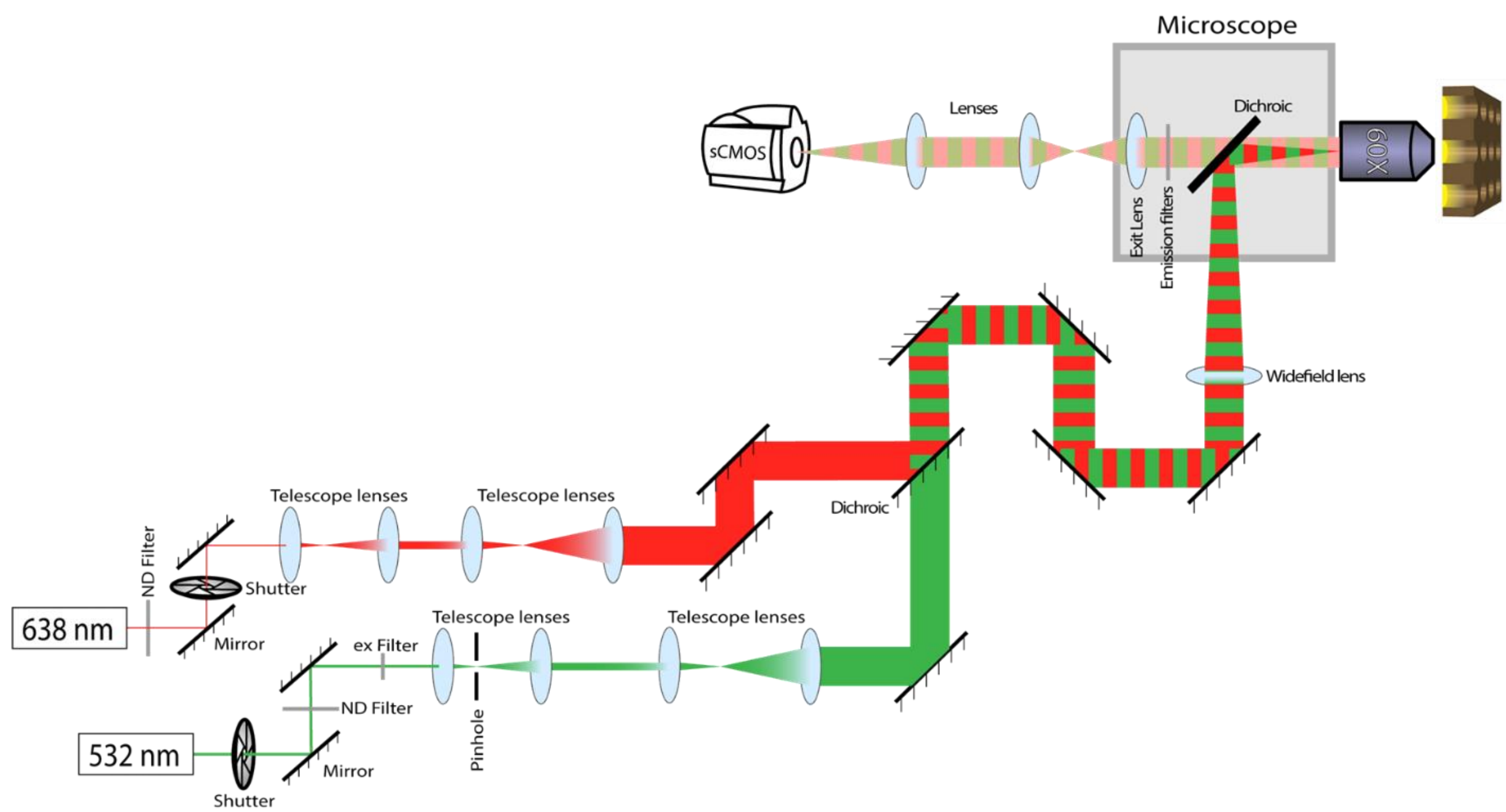

Figure S2. Diagram of optical setup.

1.3 Data Analysis

Videos were analyzed using Fiji. Brightfield images and max intensity z-projection fluorescence images were used to correct for xy drift during data acquisition. The ZMW array serves as a well-defined registration image. Max intensity z-projection fluorescence images were used to identify fluorescent molecules, which were then selected by picking a circular region of interest 5 pixels in diameter. Intensity traces over time were extracted and fed into a custom MATLAB code to plot and save in a format that can

be read by AutoDISC.[3, 4] Traces were idealized using AutoDISC with a threshold alpha value of 0.01. Idealized intensity traces were extracted and analyzed in MATLAB to extract and plot kinetic parameters of interest. Code to generate the plots in Figure 4 was adapted from Ref 5. Custom MATLAB code was used to reformat idealized intensity traces for QuB analysis. Each model was optimized using several sets of starting parameters to increase the likelihood of finding the global minimum. If optimization of the same model using different starting parameters resulted in different rates and log likelihoods (LL), the optimization with the least negative LL was selected. Traces that showed rebinding but not catalyst presence were not included in any kinetic analysis, as the presence and number of catalysts could not be confirmed.

1.4 ZMW Fabrication

ZMWs were fabricated at the Center for Nanoscale Materials facility at Argonne National Lab using positive-tone electron-beam lithography. Cover glasses (Fisher Scientific Cat. No. 12548C or Cat. No. 12541039, 130-170 µm thickness, 25x25 mm) were cleaned by soaking in piranha solution for 25 minutes at 120°C. Substrates were rinsed with DI water that had been heated to 60°C, rinsed in a cascade bath three times, dried with filtered, dry nitrogen gas from a hand-held nozzle, and oxygen plasma-cleaned with a March CS-1701 for 10 minutes at a pressure of 200 mTorr, a power of 150 W, and 30 standard cubic centimeters (sccm) gas flow to remove any remaining organic impurities on the surface. 100 nm of aluminum was deposited onto the substrates at a rate of 1 Å/s on a Temescal FC2000 e-beam evaporator. An internal quartz crystal was used to measure the thickness of the aluminum layer in situ. The substrates were then spin-coated with positive tone electron-beam photoresist ZEP520A (ZEONREX Electronic Chemicals) at 3000 rpm for 35 seconds followed by baking for 2 minutes at 180°C to a final thickness of 425 nm. ZMW features of 60, 80, 100, and 150 nm diameter dots as well as a large registration arrow and cross were patterned using a JEOL JBX-8100FS e-beam lithography system with a dose of 650 µC cm^{-2} (60 nm), 575 µC cm^{-2} (80 nm), 500 µC cm^{-2} (100 nm, arrow, and cross), and 450 µC cm^{-2} (150 nm), 100 kV

acceleration voltage, a 4 nA beam current, and a beam spot size of 5 nm. Substrates were developed in xylenes with agitation for 45 seconds, then agitated in isopropanol for 15 seconds and dried with filtered, dry nitrogen. Prior to etching, the substrates were put through a 20 second oxygen plasma descum (170 mTorr, 20W, 24 sccm O_2). 100 nm of aluminum was dry etched in an Oxford Instruments PlasmaPro 100 Cobra inductively coupled plasma reactive ion etcher by exposing the substrate to 5 sccm chlorine and 30 sccm boron trichloride for 7 seconds (breakthrough) then 20 sccm chlorine and 30 sccm boron trichloride for 50 seconds (etch) at 50°C. The remaining photoresist was removed by soaking in Microposit Remover 1165 at 90°C for twelve hours. Substrates were rinsed with DI water twice, then rinsed in IPA and dried with filtered, dry nitrogen gas from a hand-held nozzle. This procedure resulted in arrays of round ZMW wells of 60, 80, 100, and 150 nm diameter and 2 µm pitch confirmed by scanning electron microscopy.

2. Supplemental Figures

2.1 Beer's Law Plot Used to Calculate Concentration of **2**

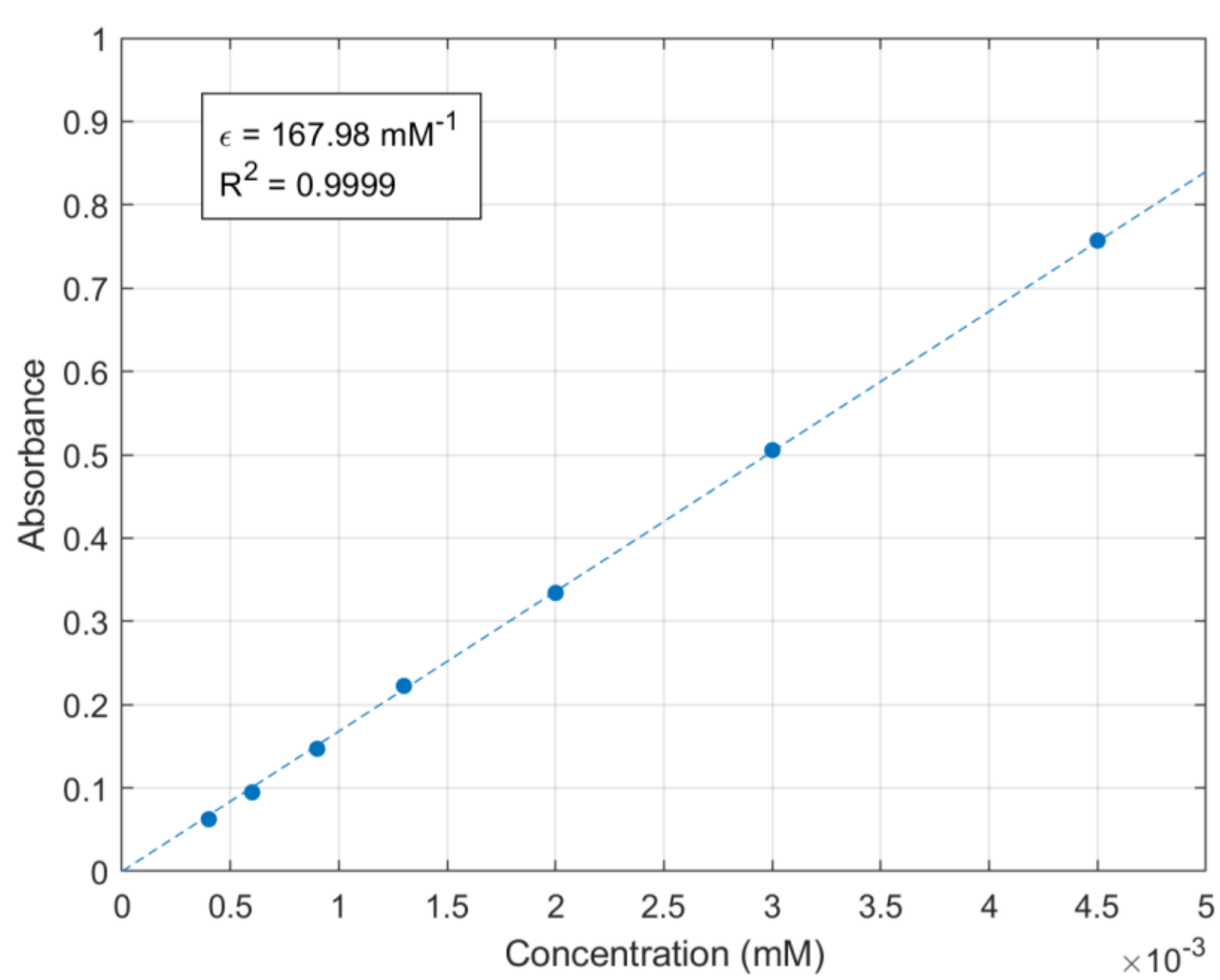

Figure S3. Beer's Law plot for the red BODIPY dye.

2.2 Additional Examples of Rebinding Traces Without Coupling Partners

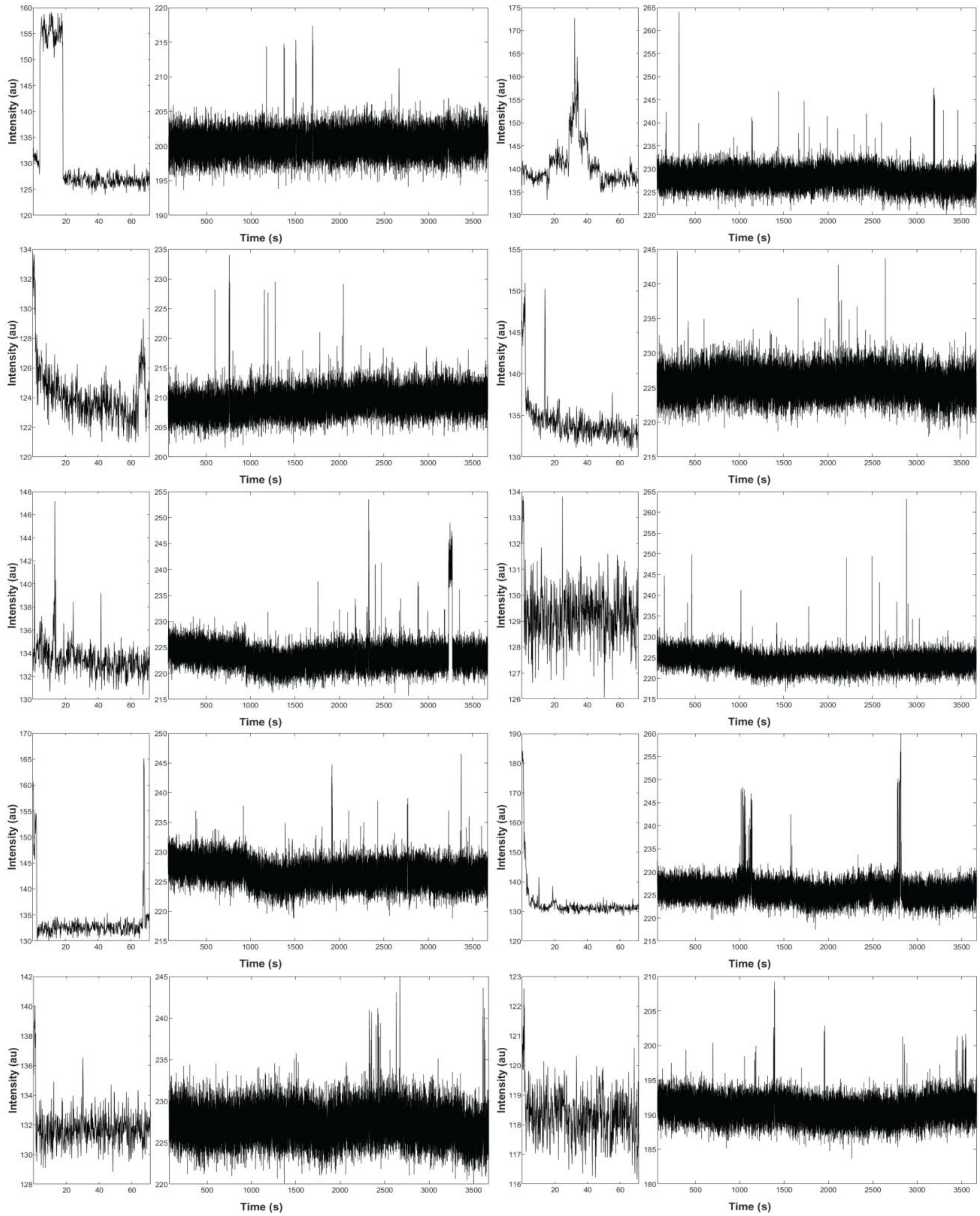

Figure S4. Representative traces for rebinding experiments without coupling partners.

2.3 Additional Examples of Rebinding Traces With Coupling Partners

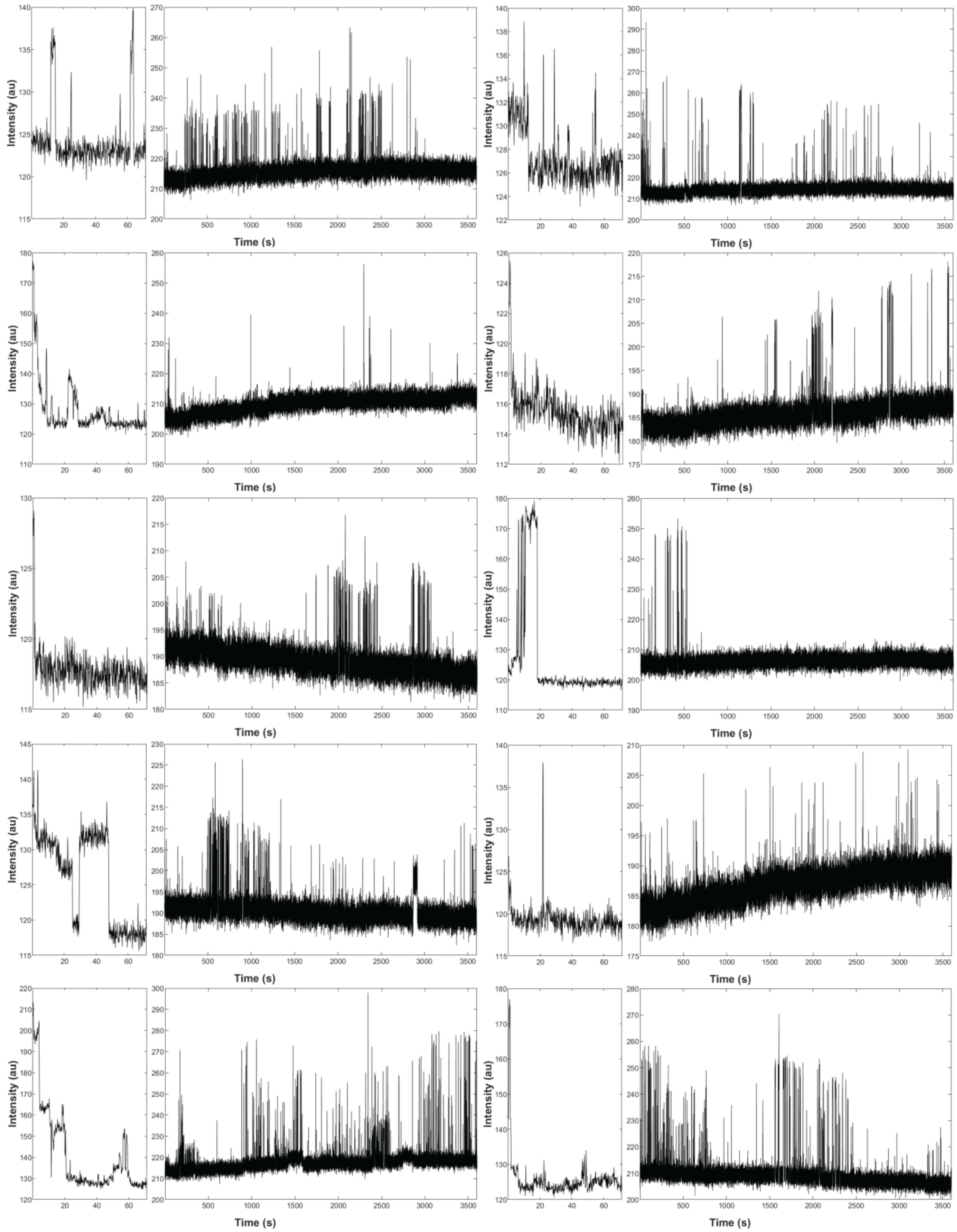

Figure S5. Representative traces for rebinding experiments with coupling partners.

2.4 Standard Errors of Rates in Scheme from Figure 5

The rates shown in Figure 5 are robust to choice of initial guess of parameters. A version with standard errors as outputted by QuB is shown below.

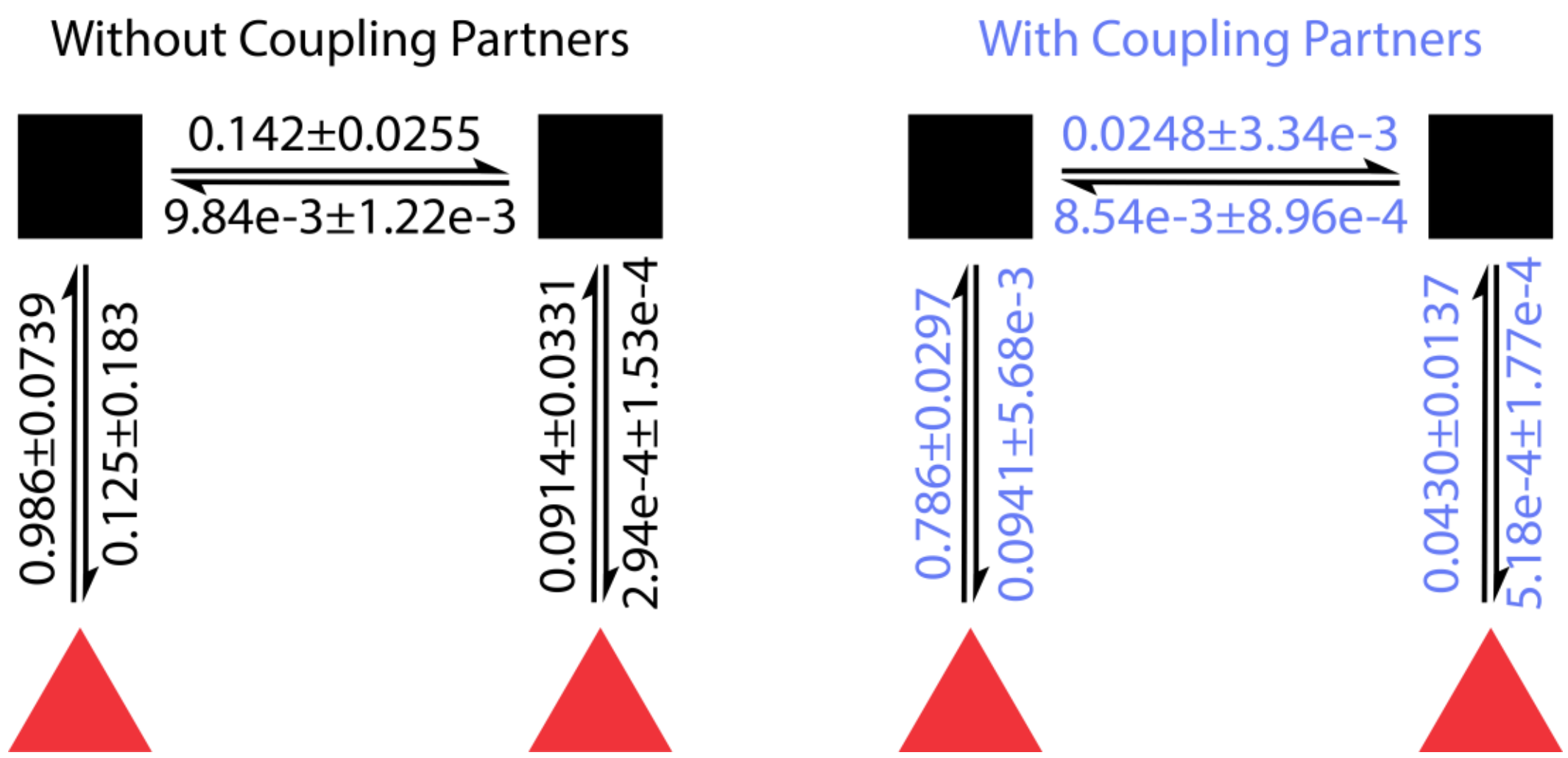

Figure S6. Version of Figure 5 scheme with rates and standard errors (s^{-1}) determined by QuB.

3. Supplemental Analysis

3.1 Effect of the BODIPY Label on Ligand Dynamics

The spiro red BODIPY was chosen to functionalize the pyridine throw-away ligand due to its fluorescence properties being well suited for a single-molecule experiment. However, the addition of the large BODIPY moiety may affect the steric and electronic properties of the ligand. As one indicator of how the BODIPY affects reactivity, we calculated the pK_a of **2** (Table S1). The computed pK_a difference between **2** and 4-methyl pyridinium is 0.45-1.13 depending on the basis set (see details below). Regarding sterics, the diffusion constant of **2** will likely be somewhat lower than an unlabeled pyridine molecule, however this difference should be negligible on the timescale of rebinding being observed in these experiments.

Molecule	Experimental pK_a	Computed pK_a (pyr ref)
4-methyl pyridinium	5.98	6.46
**2**	—	6.01

Table S1. Computed and experimental pK_a of 4-methylpyridinium and computed pK_a of **2** against the reference pyridinium cation. Structures optimized with B3LYP and the 6-31G* basis set.

Computational Details

As a general matter, for a given base B, the pK_a value of the conjugate acid BH^+ can be computed by first computing the Gibbs energy of the protonation:

$$\Delta G^0 = G^0(BH^+) - G^0(B) - G^0(H^+) \quad \text{(Eq. S1)}$$

and then relating this ΔG^0 value to pK_a in the usual way:

$$pK_a = \frac{-\Delta G^0}{2.303RT} \quad \text{(Eq. S2)}$$

However, in practice the energy of a solvated proton is very challenging to compute.[7] As a result, it is common practice to compute pK_a in reference to the known experimental pK_a of a reference molecule.[7] In this case, we computed the Gibbs energy of proton exchange between the base B and the conjugate acid of the reference $RefH^+$:

$$\Delta G^0 = G^0(BH^+) + G^0(Ref) - G^0(B) - G^0(RefH^+) \quad \text{(Eq. S3)}$$

which can be related to the

$$pK_a(BH^+) = pK_a(RefH^+) - \frac{\Delta G^0}{2.303RT} \quad \text{(Eq. S4)}$$

To demonstrate this approach and to validate the methods and basis sets selected, we considered the model system 4-methylpyridine, the conjugate acid of which has a known experimental pK_a of 5.98.[8] We used as reference the pyridinium cation, with a known experimental pK_a of 5.23,[8] which yields the results in Table S2.

Method	Basis	Computed pK_a	Error wrt Experiment
RHF	6-31G*	6.54	0.56
B3LYP	6-31G*	6.46	0.48
MP2	6-31G*	6.25	0.27
RHF	6-31G**	6.57	0.59
B3LYP	6-31G**	6.51	0.53
MP2	6-31G**	6.21	0.23
RHF	6-31++G**	6.63	0.65
B3LYP	6-31++G**	6.49	0.50
MP2	6-31++G**	6.28	0.30

Table S2. Computed pK_a of 4-methylpyridinium against the reference pyridinium cation. All structures optimized with B3LYP and the corresponding basis sets.

As demonstrated in Table S2, across all basis sets used here, the B3LYP density functional calculations reproduce the experimental pK_a with an accuracy of approximately half a pK_a unit. With this validation in mind, the pK_a of **2** can be computed in the same way using both pyridinium (pK_a = 5.23) and 2-methylpyiridinium (pK_a = 5.98) as reference acids (Table S3).

Method	Basis	pK_a (pyr ref)	pK_a (4-me pyr ref)
B3LYP	6-31G*	6.01	5.53
B3LYP	6-31++G**	5.36	4.86

Table S3. Computed pK_a of **2** against the reference pyridinium cation (pyr ref) and 4-methyl pyridinium (4-me pyr ref).

3.2 Data Distribution by ROI

Another plausible explanation for the multiexponential kinetics in Figure 4 is that the non-monoexponential behavior originates from multiple static subpopulations of molecules. To examine for catalyst-to-catalyst heterogeneity, the t_{ons}, t_{offs}, and time of signal initialization (the time, relative to the start of the rebinding portion of the experiment, when each rebinding event is detected, i.e., the beginning of each "on" interval) was extracted for each ROI used in the kinetic analysis. Inspection of the rebinding traces below reveals that a range of t_{on}, t_{off}, and time of signal initialization values are observed from nearly all traces, suggesting that, while molecule-to-molecule static heterogeneity certainly exists, it is not principally responsible for the heterogeneous kinetics observed.

Without Coupling Partners

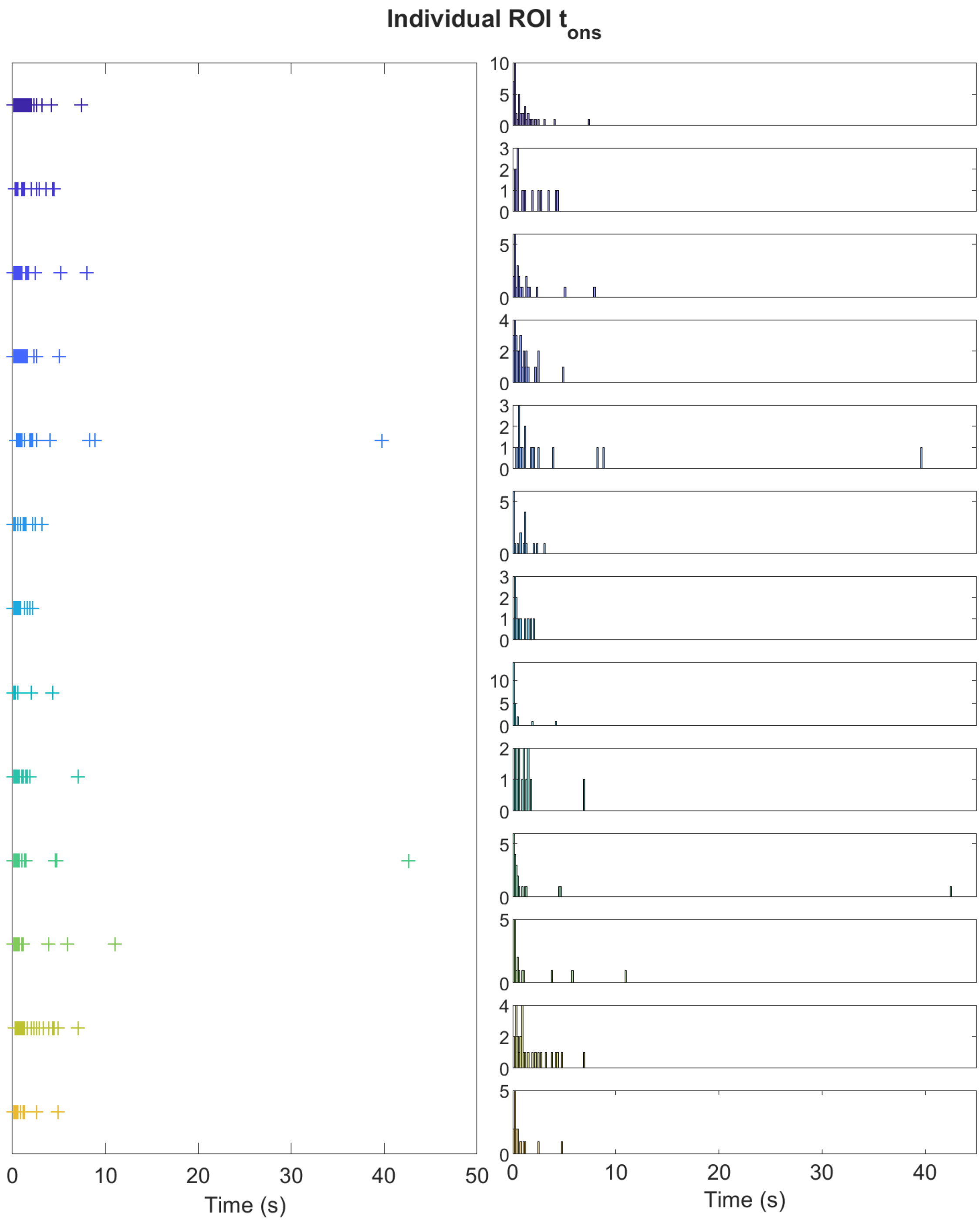

Figure S7. Distribution of t_{ons} used in the kinetic analysis for initiation experiments without coupling partners separated by ROI. Data shown as a scatter plot (left) and a histogram (right). Each row/color represents a separate ROI. To see data distribution scaled to show short time events and exclude the few large time data points, see Figure S8.

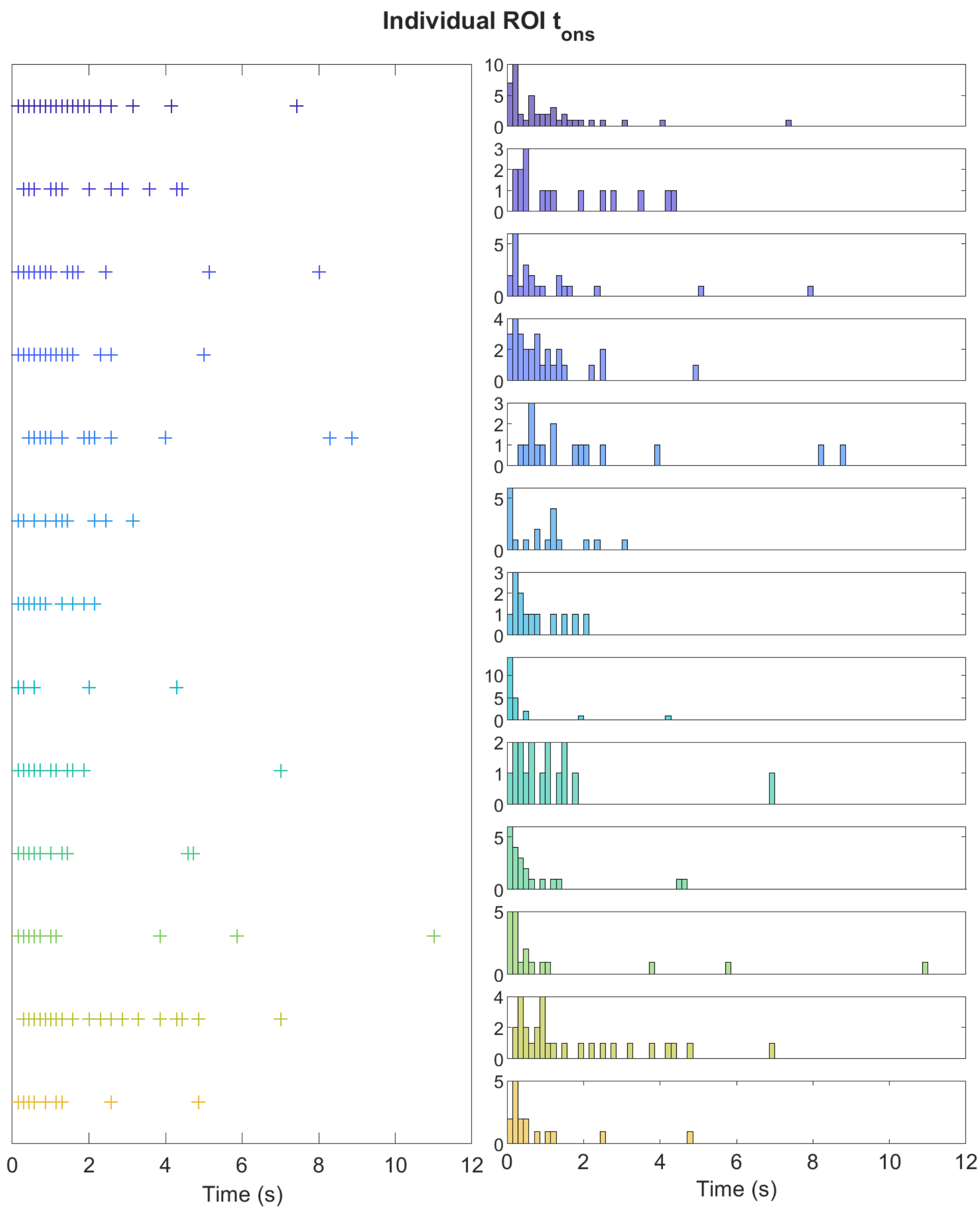

Figure S8. Scaled distribution of t_{ons} used in the kinetic analysis for initiation experiments without coupling partners separated by ROI. Data scaled to show data distribution for the majority of data points (excludes two data points with long times). For the full distribution, see Figure S7. Data shown as a scatter plot (left) and a histogram (right). Each row/color represents a separate ROI.

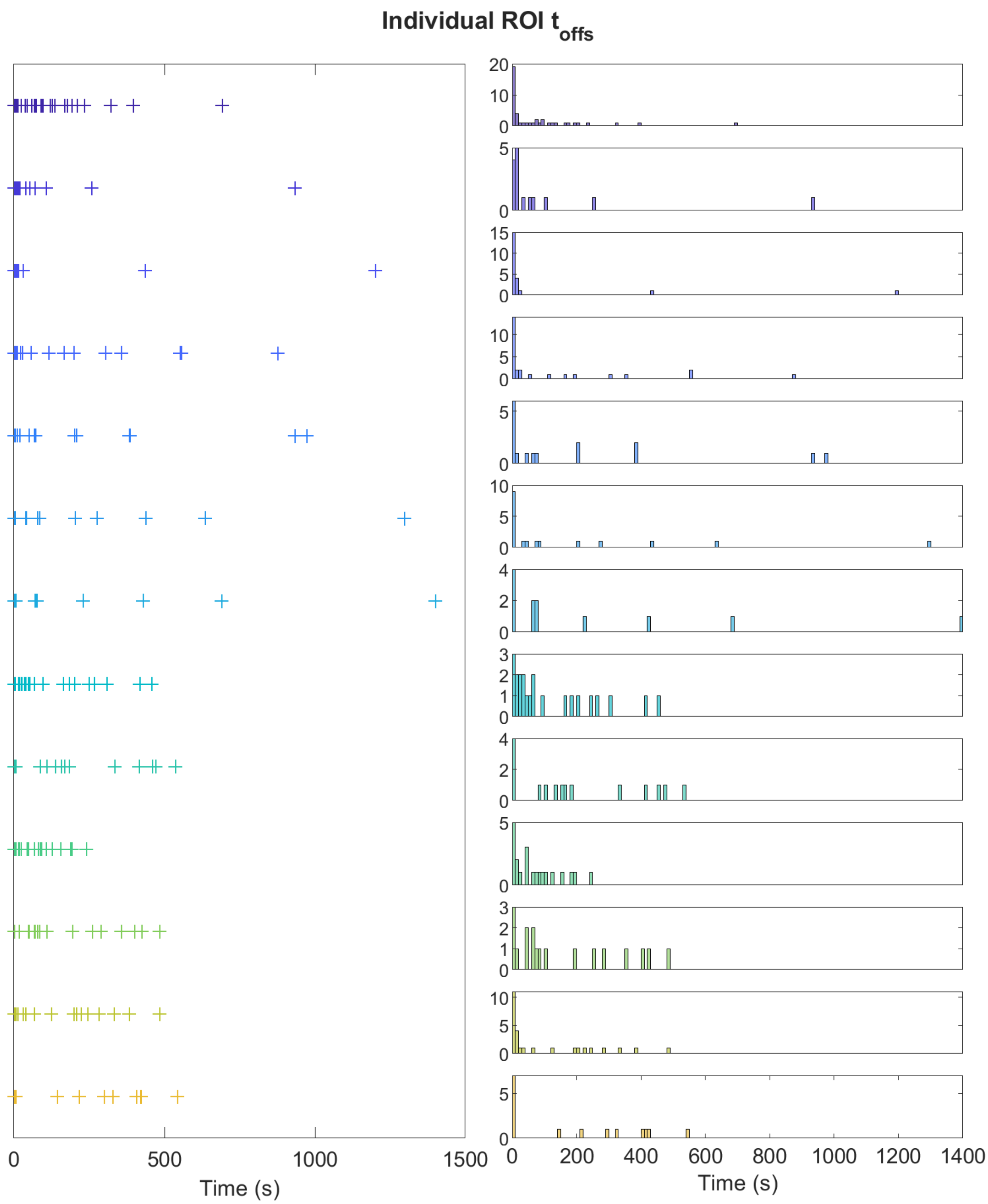

Figure S9. Distribution of t_{offs} used in the kinetic analysis for initiation experiments without coupling partners separated by ROI. Data shown as a scatter plot (left) and a histogram (right). Each row/color represents a separate ROI.

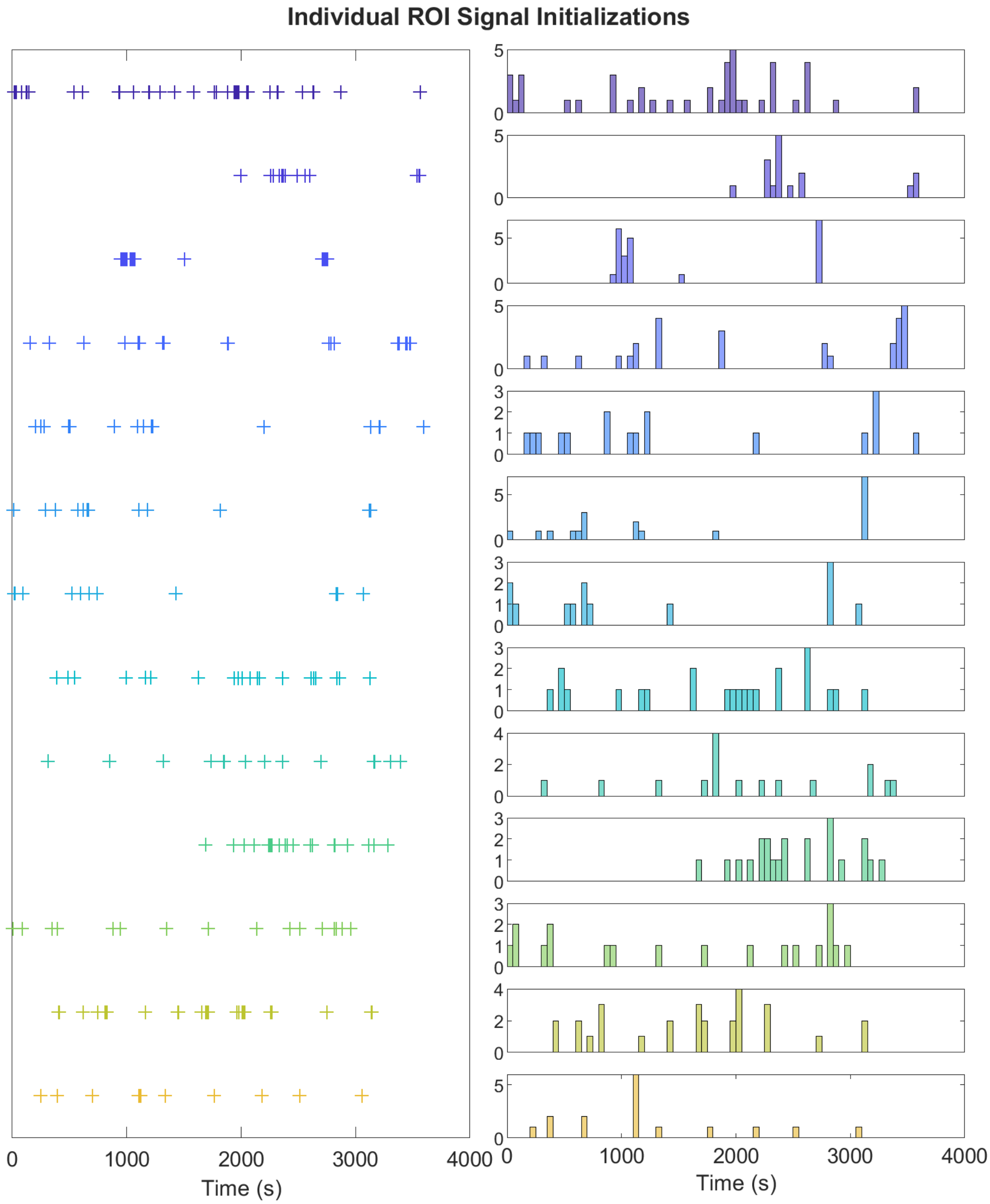

Figure S10. Distribution of times of signal initialization of rebinding events used in the kinetic analysis for initiation experiments without coupling partners separated by ROI. Data shown as a scatter plot (left) and a histogram (right). Each row/color represents a separate ROI.

With Coupling Partners

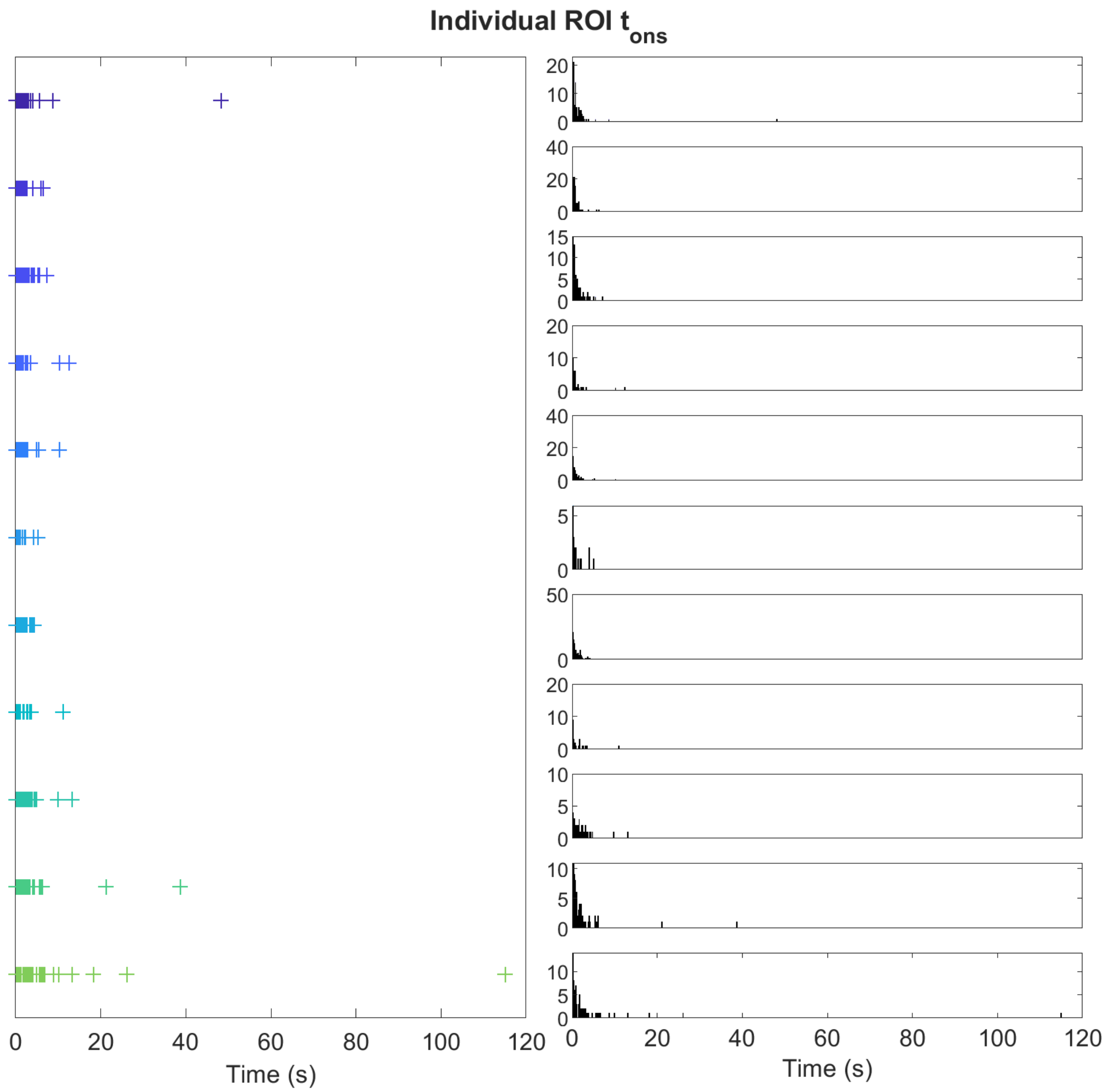

Figure S11. Distribution of t_{ons} used in the kinetic analysis for initiation experiments with coupling partners separated by ROI. Data shown as a scatter plot (left) and a histogram (right). Each row/color represents a separate ROI. To see data distribution scaled to show short time events and exclude the few longest time data points, see Figure S12.

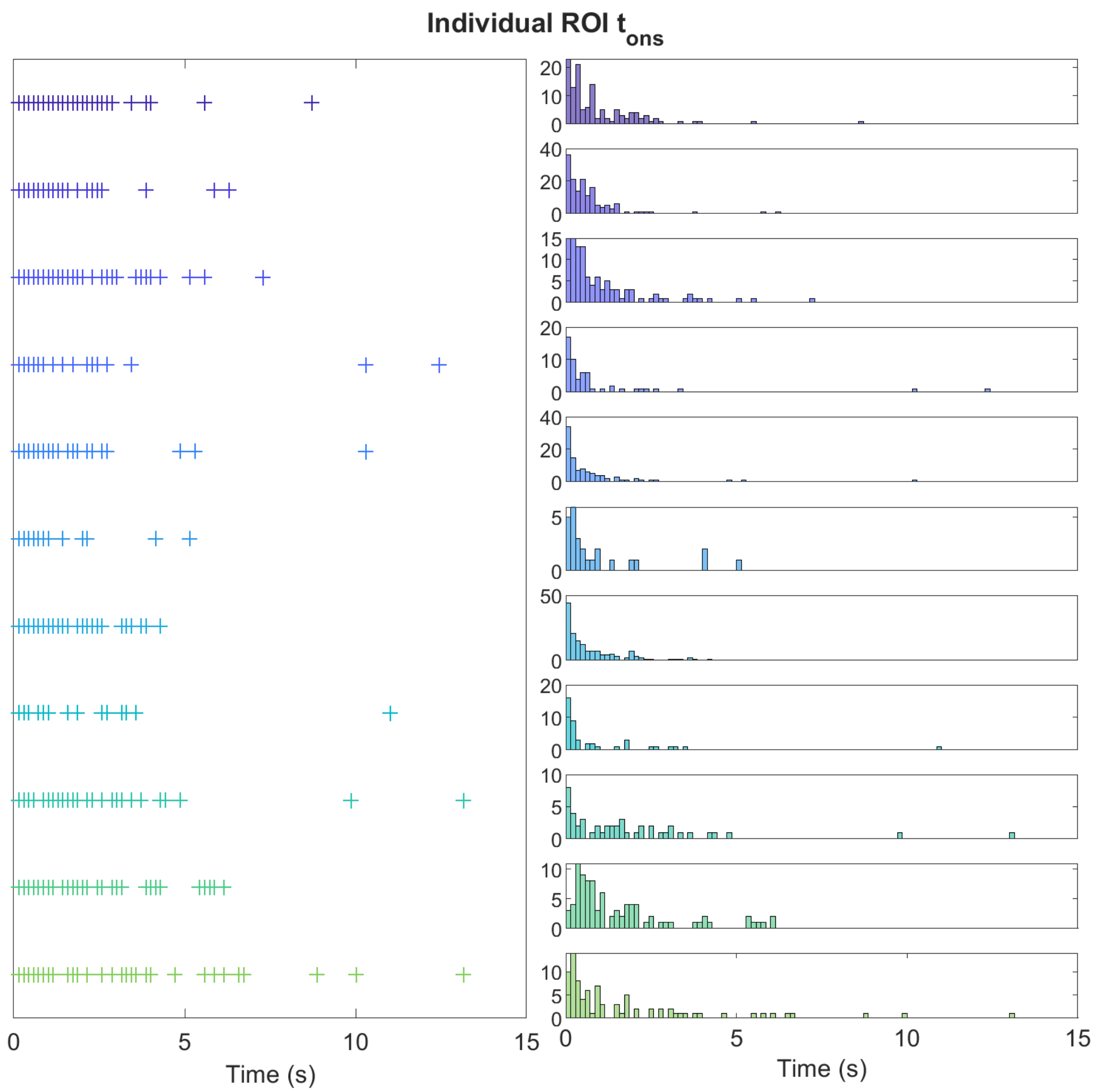

Figure S12. Scaled distribution of t_{ons} used in the kinetic analysis for initiation experiments with coupling partners separated by ROI. Data scaled to show data distribution for the majority of data points (excludes six data points with longest times). For the full distribution, see Figure S11. Data shown as a scatter plot (left) and a histogram (right). Each row/color represents a separate ROI.

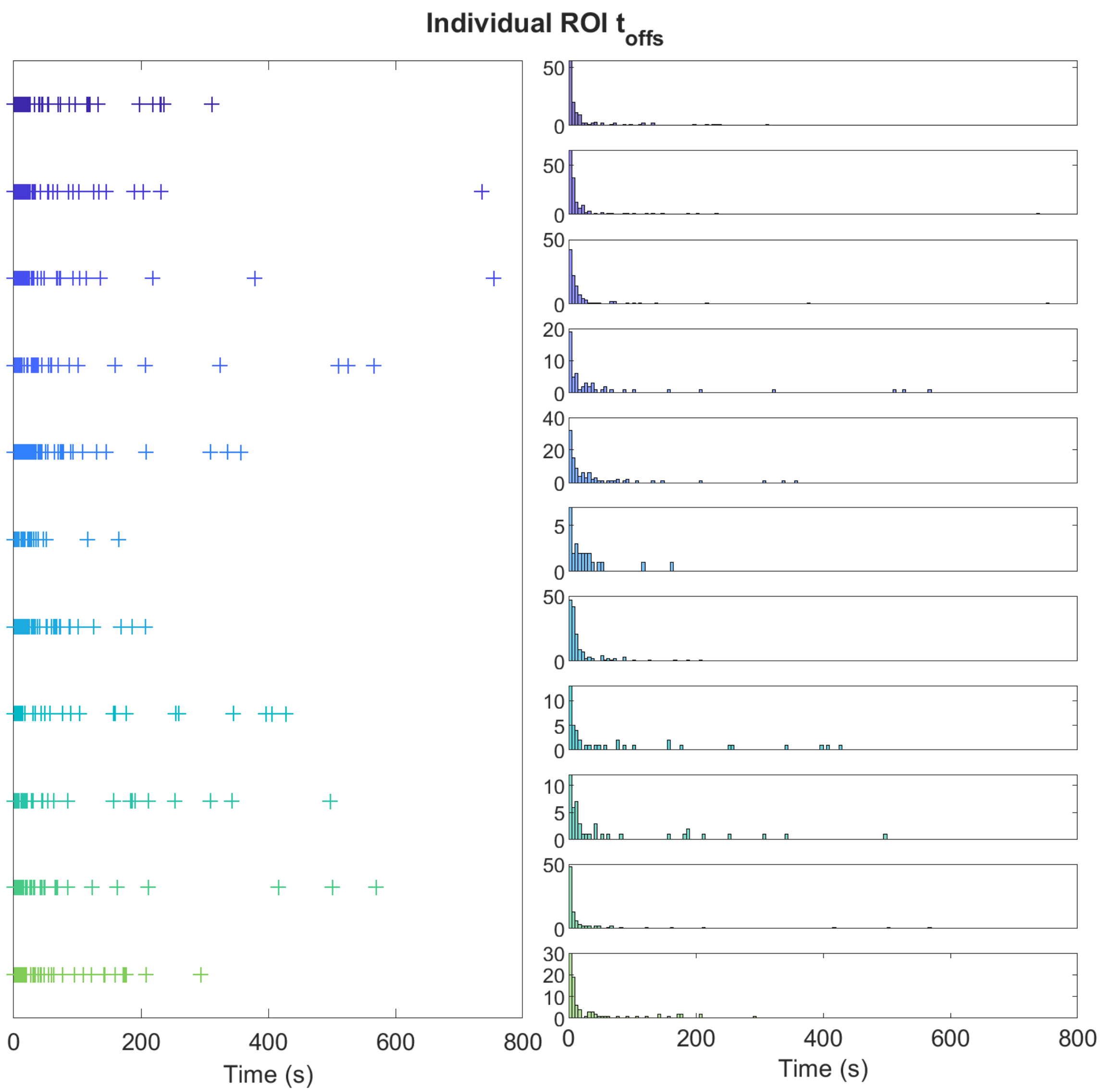

Figure S13. Distribution of t_{offs} used in the kinetic analysis for initiation experiments with coupling partners separated by ROI. Data shown as a scatter plot (left) and a histogram (right). Each row/color represents a separate ROI. To see data distribution scaled to show short time events and exclude the few longest time data points, see Figure S14.

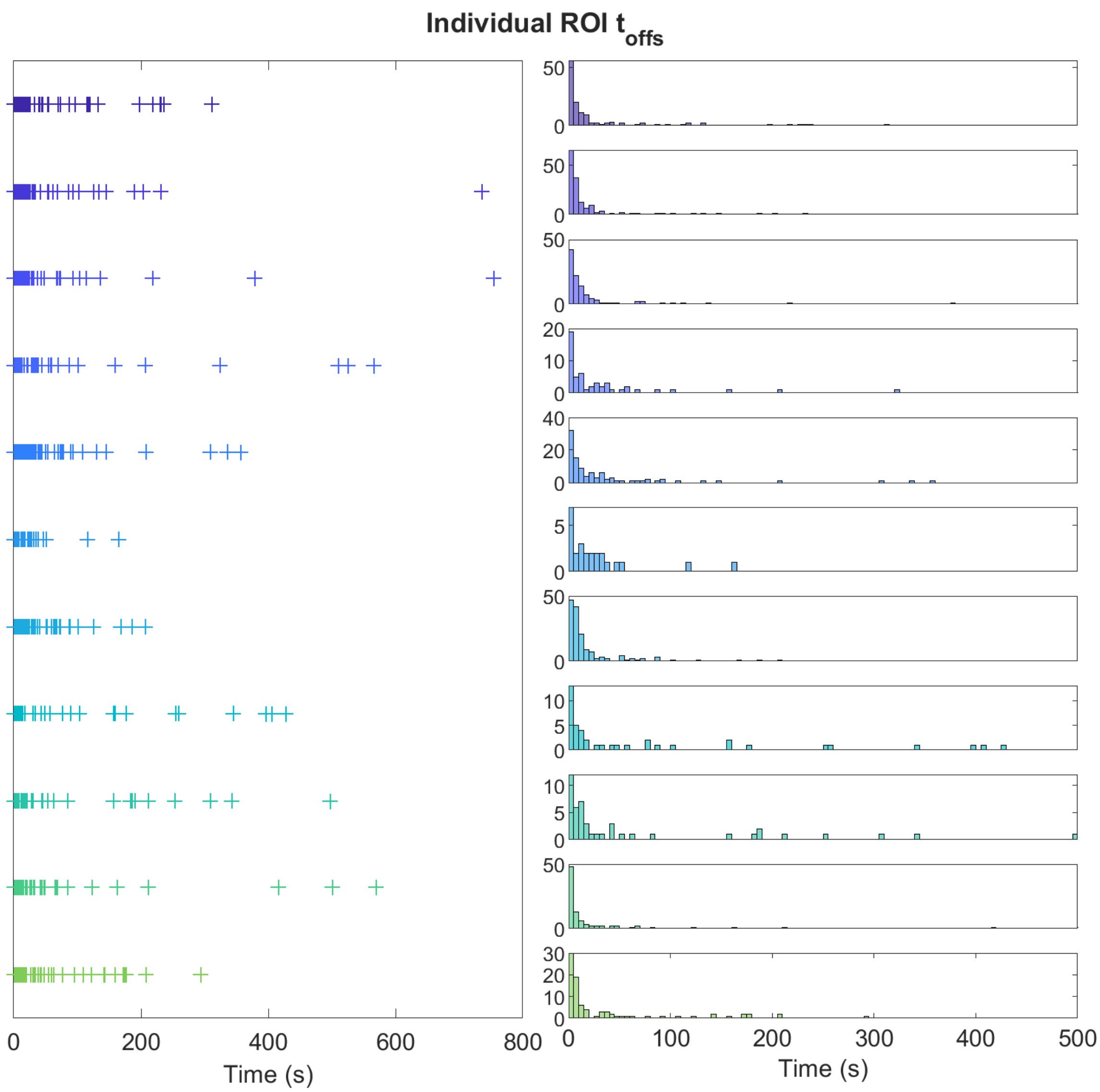

Figure S14. Scaled distribution of t_{offs} used in the kinetic analysis for initiation experiments with coupling partners separated by ROI. Data scaled to show data distribution for the majority of data points (excludes seven data points with longest times). For the full distribution, see Figure S13. Data shown as a scatter plot (left) and a histogram (right). Each row/color represents a separate ROI.

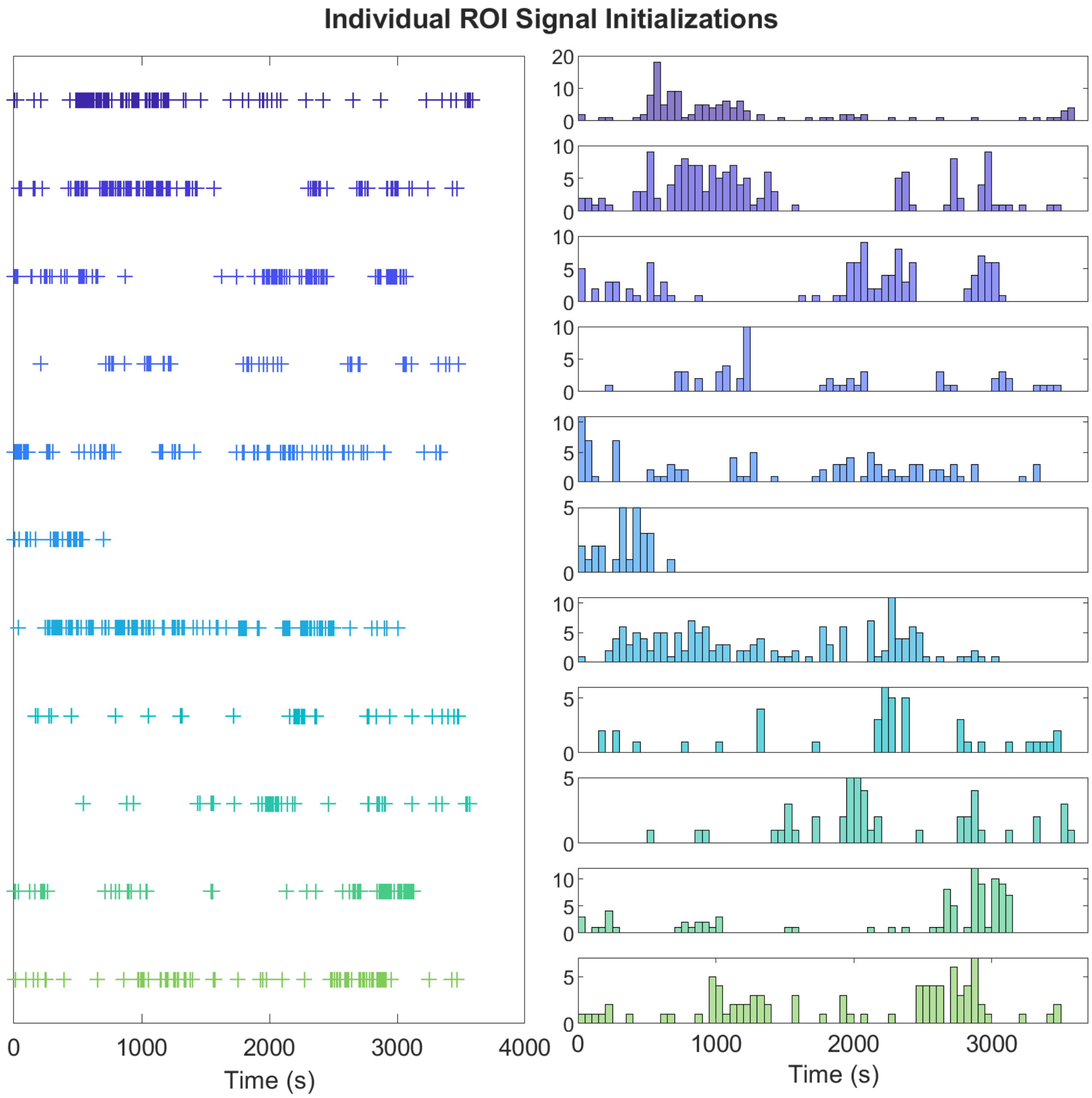

Figure S15. Distribution of times of signal initialization of rebinding events used in the kinetic analysis for initiation experiments with coupling partners separated by ROI. Data shown as a scatter plot (left) and a histogram (right). Each row/color represents a separate ROI.

3.3 Model Selection for Mechanistic Discussion

There are 24 unique two-, three-, or four-state models with one to six reversible connections between states. All are depicted graphically below, with log likelihood (LL) values (determined by QuB) and Bayesian Information Criteria (BIC)[9] values for single-molecule data without reactants (black) and with reactants (blue) (Figure S16). BIC penalizes models for containing excess parameters. For LL values (less

negative indicates a better fit), more complex models always yield improved values. However, the BIC values (lower value is better fit) are seen to reach a minimum with four states and three connections. BIC is calculated as follows:

$$BIC = -2 \cdot LL + k \cdot \ln(N) \qquad \text{(Eq. S5)}$$

Here, LL refers to the ln(likelihood) [note, there is inconsistency in sources with LL sometimes referring to **L**ike**L**ihood and other times **L**og(**L**ikelihood). We consistently use LL to mean ln(likelihood) in this text]. k refers to the number of free kinetic parameters, equal to twice the number of connections for each model (since each double-sided arrow includes two rates). N is the total number of experimental on and off intervals: 557 for without reagents and 1953 for with reagents. As an example, for the scheme in the first row of Figure S16 (two states, one connection) without coupling partners, the BIC is calculated as:

$$\begin{aligned} BIC &= -2(-3042) + 2 \cdot \ln(557) \\ &= 6084.2 + 12.6 \\ &= 6096.8 \end{aligned}$$

The scheme in the green box is the one chosen for kinetic discussion. It possesses the smallest BIC value amongst all schemes for rebinding experiments with coupling reagents (blue values). For rebinding experiments without coupling reagents (black values), it is one of several on that same row (four states, three connections) with similar BIC values that are the lowest among all of the schemes. Since, as discussed in the main text, we associate these states with different molecular configurations, we exclude "diagonal" processes that would entail two molecular processes occurring at the same time. With diagonal processes excluded, the green boxed scheme has the smallest BIC values for rebinding experiments both with and without reagents.

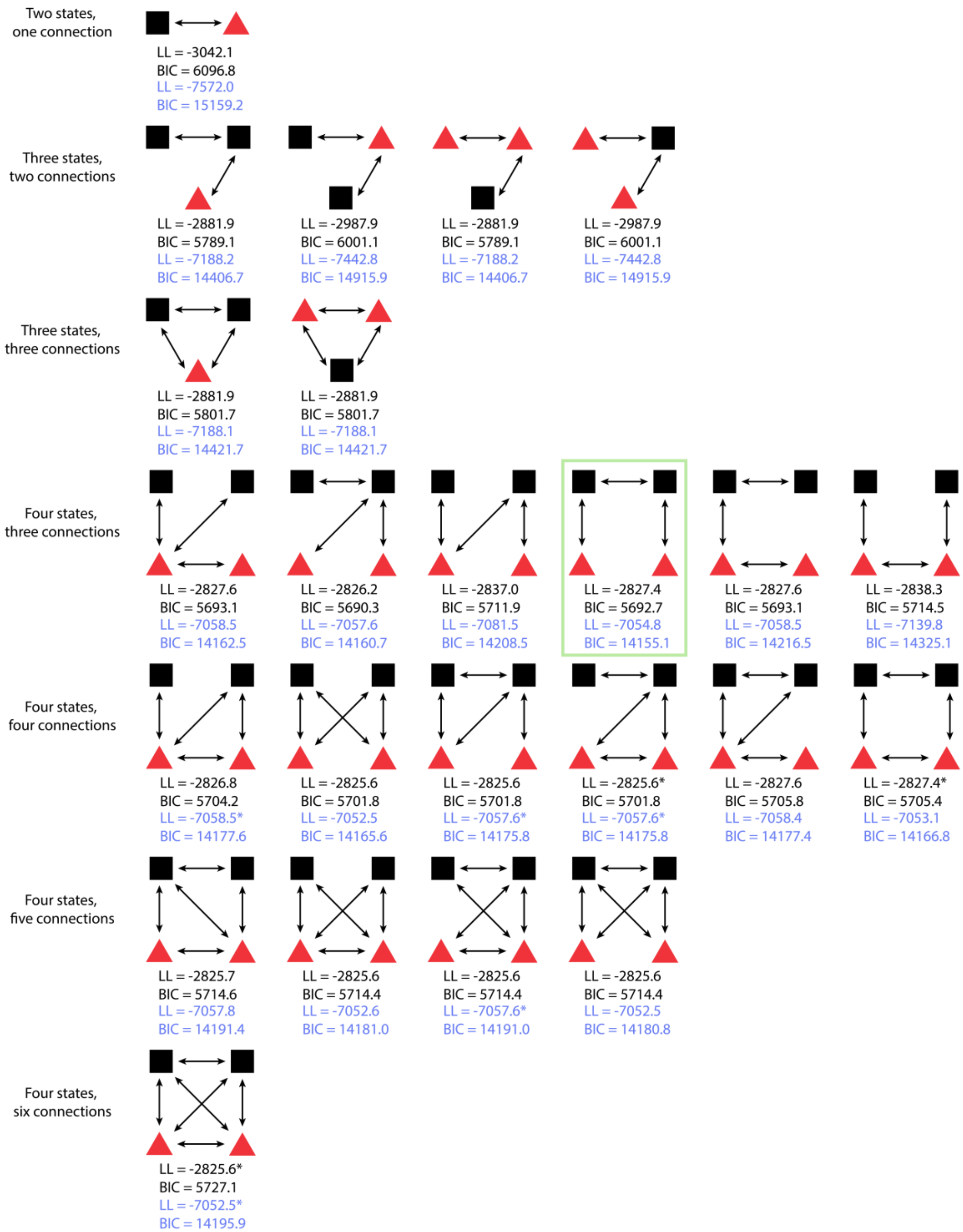

Figure S16. Comparison of different mechanistic models for the observed single-molecule fluorescence data with log-likelihood of the fit (LL) reported as determined by QuB analysis. LLs for rebinding experiments without coupling partners are shown in black and LLs for rebinding experiments with coupling partners are shown in blue. Less negative values indicate better fits. Black squares represent dark states and red triangles represent bright states. Asterisks indicate that the shown model collapsed to a simpler model (fewer connections) during optimization. Model used for further discussion shown within a green box.

3.4 Simulated Dwell Time Distribution

Taking the schemes in Figure 5 and simulating expected dynamics yields a series a monoexponential decays for the dwell times in each state, as expected from simplified single-molecule kinetics.[10, 11] After summing these monoexponential decays pairwise for the two liganded and two unliganded states (since the experiment cannot use fluorescence intensity alone to distinguish between them), biexponential decays are seen, Figure S17 and Figure S18, highly reminiscent of Figure 4. Comparing the dwell time distributions with and without coupling reagents, the distribution of bright state dwell times is largely unchanged, while the distribution of dark state dwell times is noticeably shortened upon addition of coupling reagents, as seen in Figure 4. Note, the number of counts here is higher than in Figure 4, but just reflects a larger number of simulated instances.

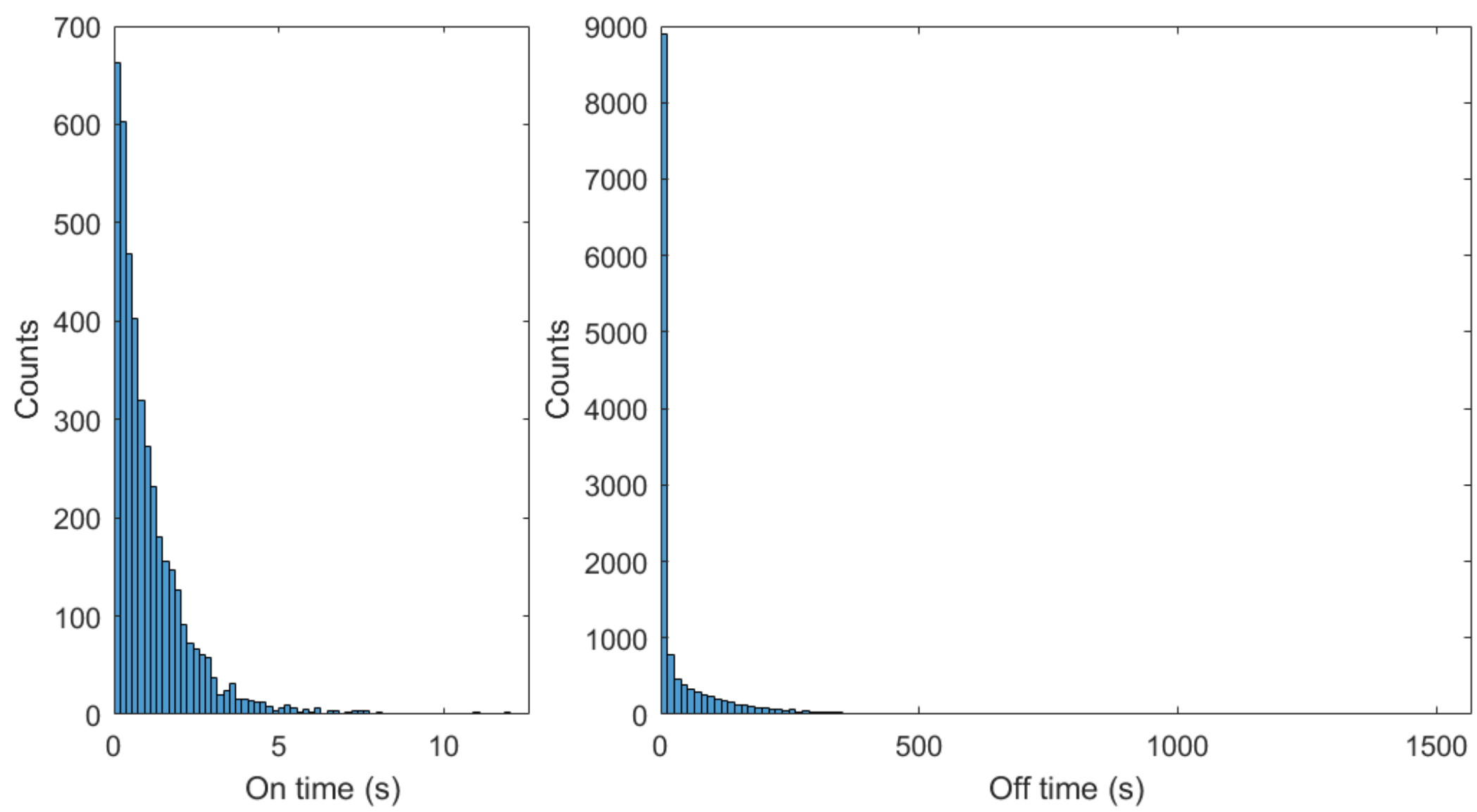

Figure S17. Simulated dwell times without coupling reagents.

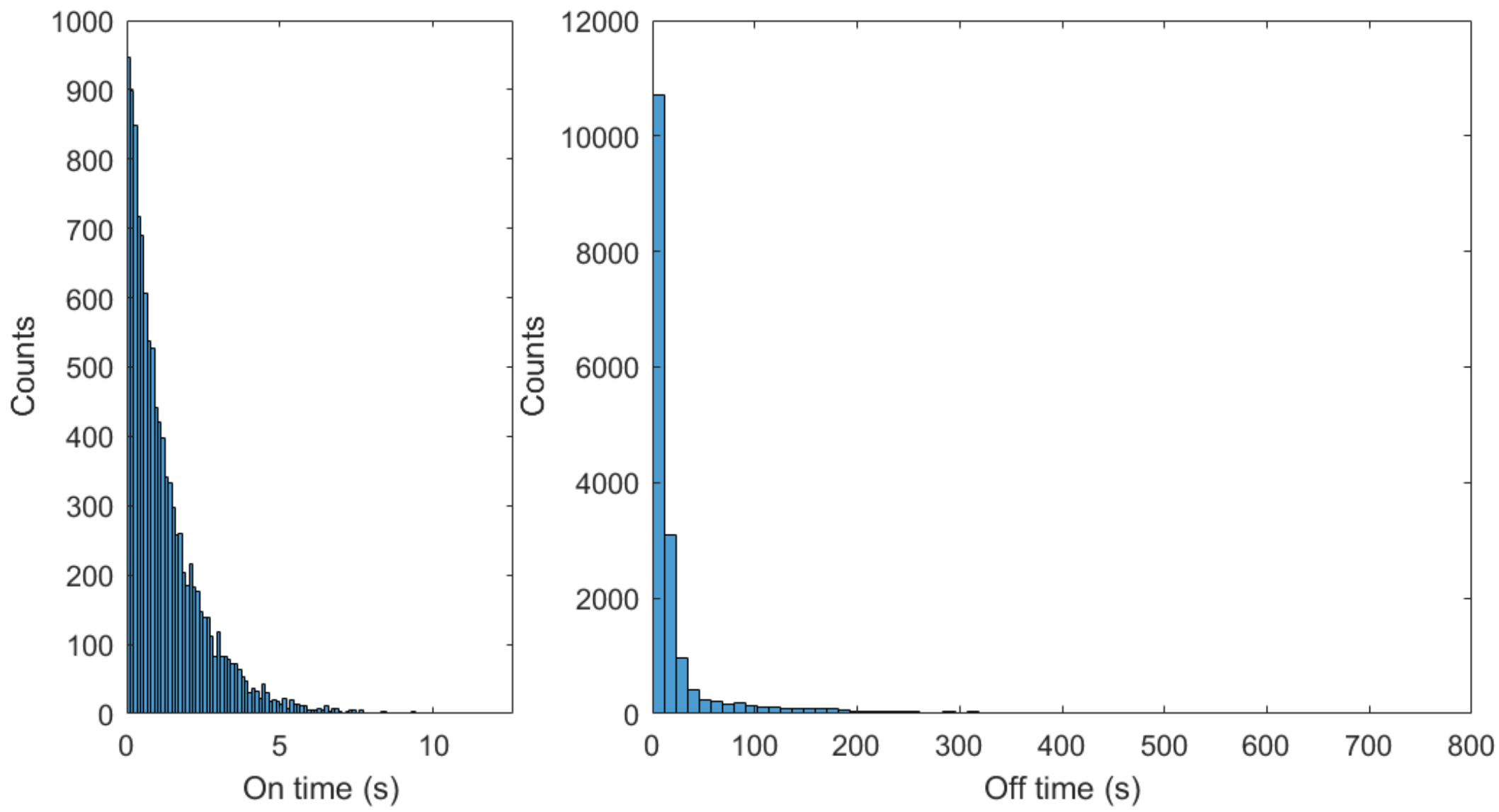

Figure S18. Simulated dwell times with coupling reagents.

4. Control Experiments

4.1 Controls for nonspecific adsorption

For rebinding experiments, ZMWs must be passivated to prevent nonspecific adsorption to the surface. Control experiments with bare waveguides showed, upon introduction of **2**, long lived fluorescent events in the wells, indicating passivation is required (Figure S19). ZMWs were passivated with 20% V/V triethoxy(octyl)silane and imaged with a solution of **2** in THF to test the effectiveness of the passivation. In the 60 nm ZMWs used in this study, no evidence of any nonspecific adsorption was observed for a 6.5 µM solution of **2** (6.5 times higher than the concentration used for rebinding experiments) using the same acquisition parameters as described above for rebinding experiments (Figure S20).

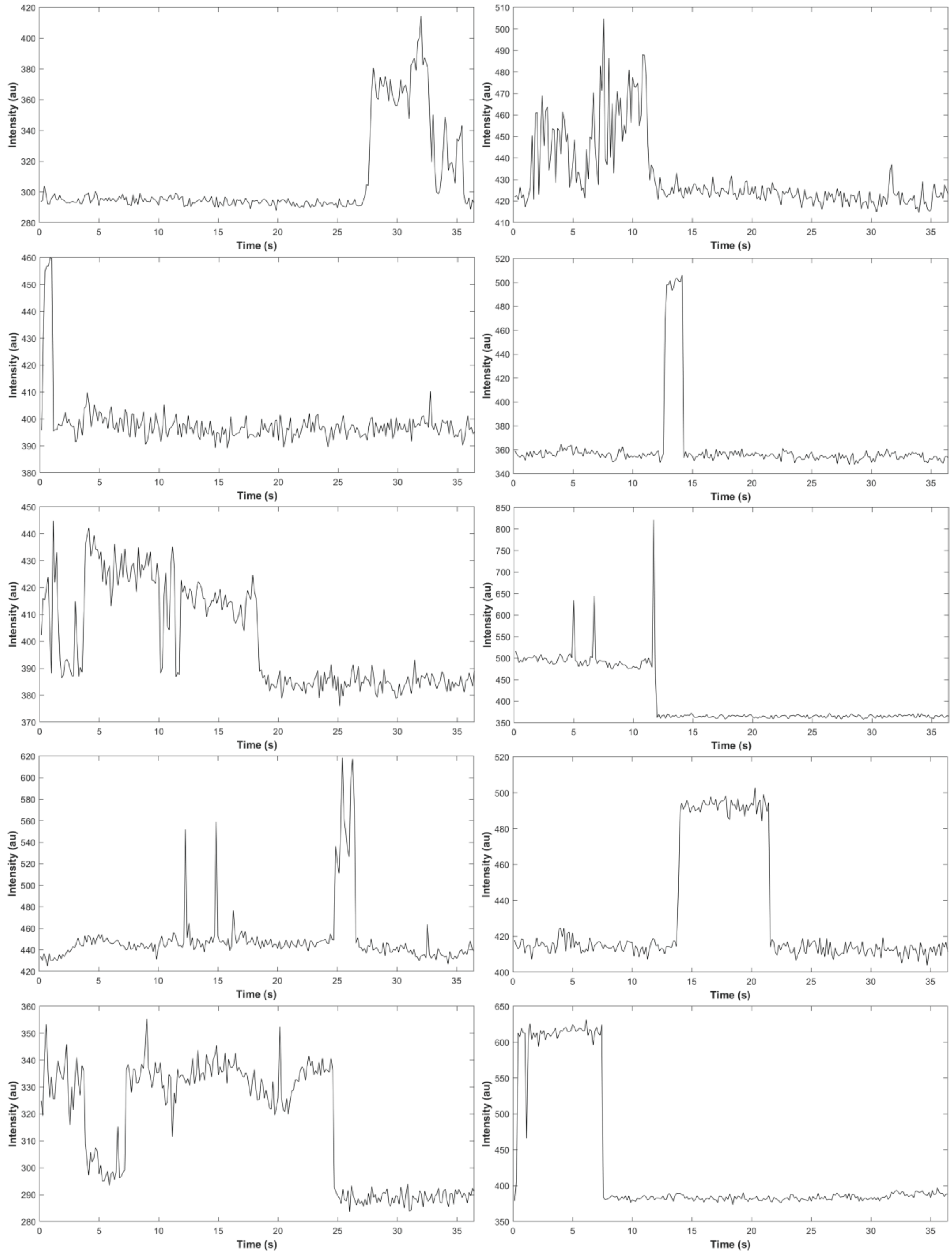

Figure S19. Representative traces from unpassivated 150 nm ZMW wells showing long-lived fluorescence of **2** is observed when added to the ZMW microscopy chamber even at lower concentrations than those used in the rebinding experiments (~nM). Maximum fluorescence intensities are higher than those in the rebinding data because no laser attenuation was used to collect this data. All other microscopy conditions are consistent with those described above.

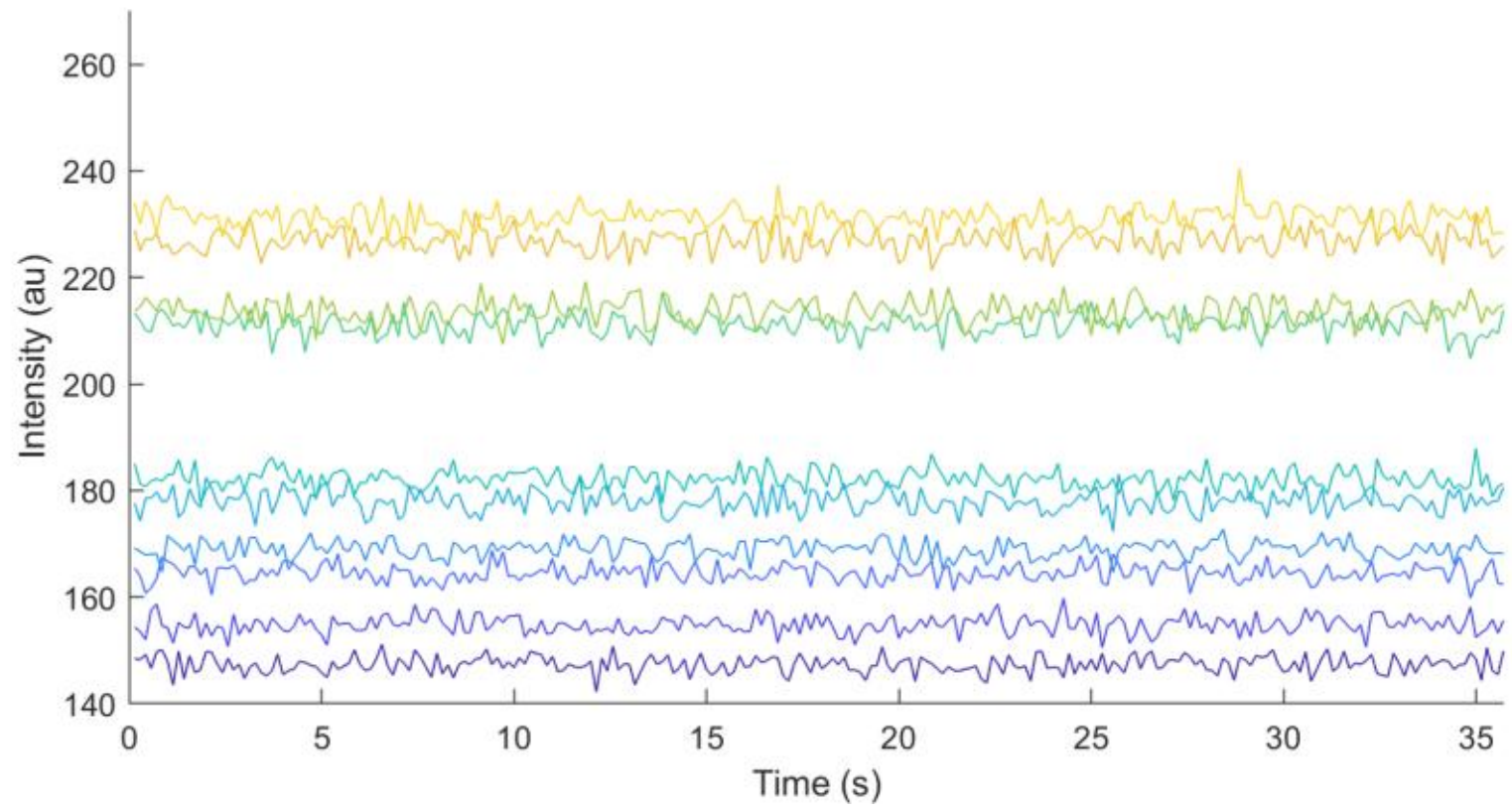

Figure S20. Ten representative traces from ZMW nonspecific sticking control experiment showing no fluorescence activity above background. All other traces analyzed in this experiment showed similar behavior. ZMWs were passivated with a 20% V/V triethoxy(octyl)silane solution using the same protocol as described above and imaged with a solution of **2** in THF (final concentration 6.5 µM) using the same microscopy conditions as rebinding studies, described above. Differences in baseline intensity are due to different well positions relative to the excitation beam resulting in different wells experiencing different excitation powers.

Controls were also completed to investigate base degradation of the passivation layer. ZMWs were passivated with 20% V/V triethoxy(octyl)silane and imaged with a solution of 0.5 mM KOtBu and 1 µM **2**. No evidence of any nonspecific adsorption was observed over the course of an hour, indicating that this passivation is robust to base under microscopy conditions and over the timescale of this experiment (Figure S21).

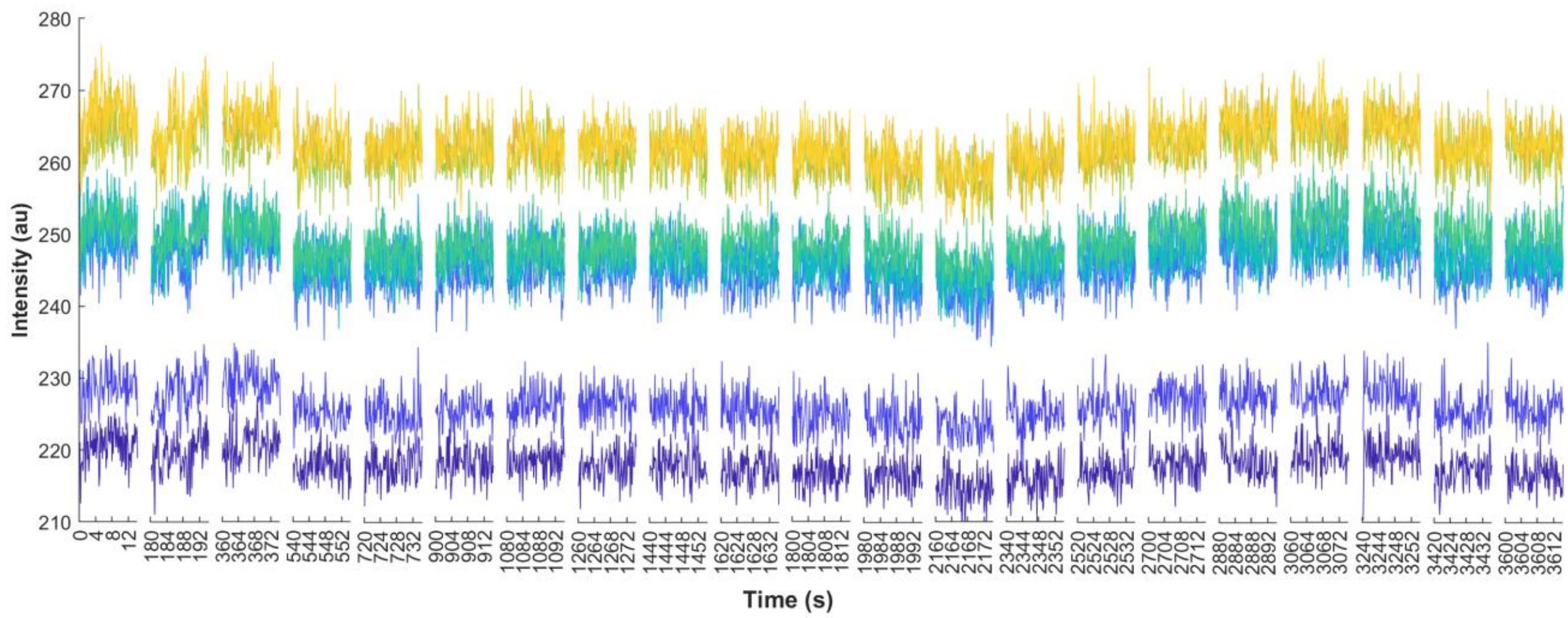

Figure S21. Ten representative traces from base degradation experiment show no fluorescence activity above background. All other traces analyzed in this experiment showed similar behavior. ZMWs were passivated with a 20% V/V triethoxy(octyl)silane solution using the same protocol as described above and imaged with a solution of fluorescent **2** (final concentration 1 μM) and initiator (final concentration 0.5 mM KOtBu) in THF using the same microscopy conditions as rebinding studies, described above. Data was collected every 3 minutes over the course of an hour. Differences in baseline intensity are due to different well positions relative to the excitation beam resulting in different wells experiencing different excitation powers.

4.2 Blinking Control Experiment

Our two-color scheme was designed to minimize the misidentification of long-lived dark states of the precatalyst label as rebinding. Still, because a variety of molecular processes can contribute to the observed kinetics, it is important to rule out some plausible alternatives. One such source is dye blinking. It is relatively common for dyes to undergo transitions into states that do not allow fluorescence emission, such as intersystem crossing into a triplet state or a more longer-lived electron transferred state, causing the observation of a temporary dark state. However, BODIPY dyes are generally known to be stable emitters with low blinking and with very low rates of intersystem crossing.[12, 13]

In order to determine if dye blinking was a major contributor to the observed dynamics, control experiments were conducted under experimental microscopy conditions using a variant on the catalyst molecule labeled with the red BODIPY ligand (**S1**).[14] Coverslip microscopy chambers were prepared as described above using a clean coverslips and glass tubes (Section 1.1). Coverslips were used for these

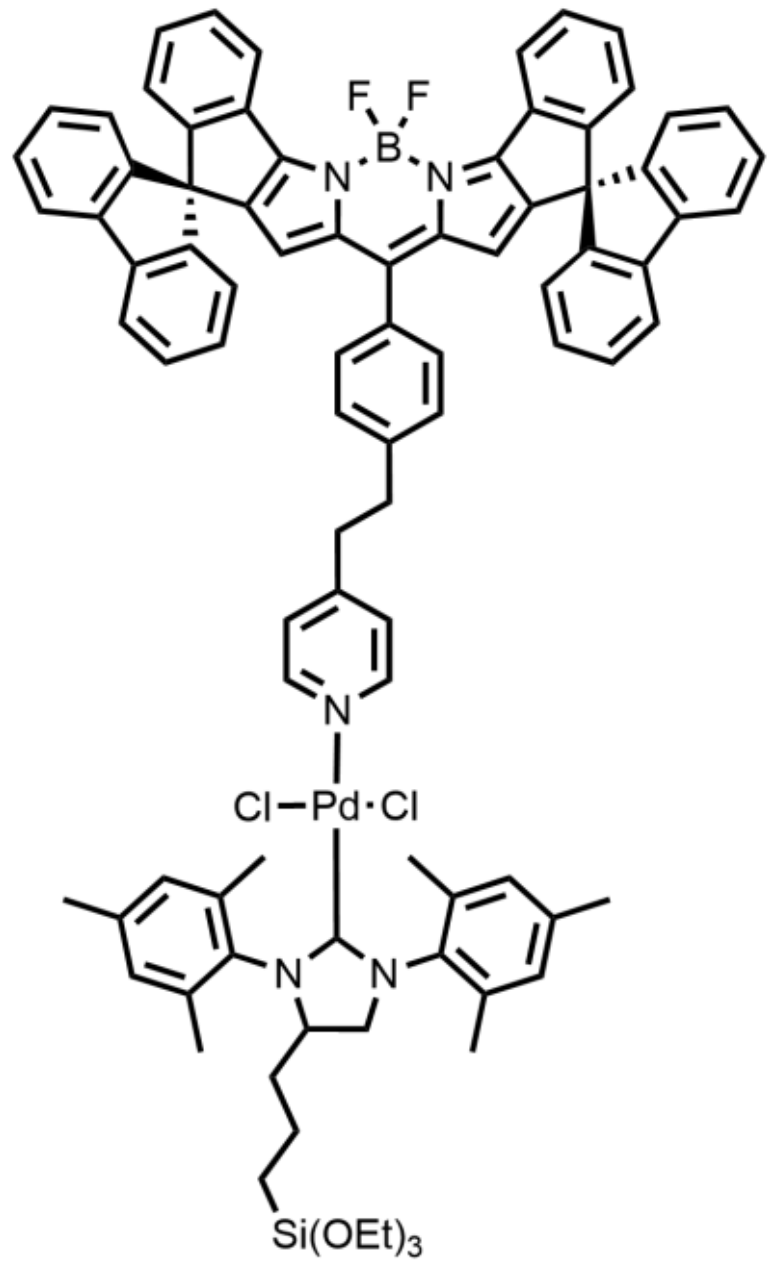

S1

experiments rather than ZMWs in order to increase the number of observed molecules per experiment and to preserve ZMWs. A solution of 5% V/V triethoxy(octyl)silane in IPA was made and used to create a dilute solution of **S1** for single-molecule deposition (~nM in **S1**). The deposition concentrations for these experiments were selected to be within ranges shown to be effective for single-molecule experiments on coverslips.[14] The coverslip chamber was cleaned as described above and 200 µL of the triethoxy(octyl)silane + **S1** solution was added to the chamber and allowed to physisorb, covered, for one hour. The solution was removed and the coverslip chamber was dried with nitrogen before being heated at 110°C for 10 minutes for to drive off water and covalently bind the passivation layer and **S1** to the surface. Before imaging, the chambers were rinsed out twice with 900 µL of HPLC grade IPA, twice with 900 µL of clean THF, then dried with nitrogen.

100 µL of clean THF was added to the chamber and the sample was imaged under 635 nm illumination using total internal reflection fluorescence (TIRF) for just over 10 minutes using the Andor Solis software (Figure S2). TIRF was used in order to minimize background fluorescence and only observe fluorescence at the surface of the chamber, similar to (although much deeper than) the evanescently decaying excitation profile of ZMWs. All other microscopy conditions are the same as those used for rebinding experiments (Section 1).

There was no evidence of large-scale blinking of **S1**, so dye blinking is very unlikely to be responsible for the observed dynamics in the rebinding experiments. In fact, many molecules showed constant fluorescent signals for several hundred seconds before photobleaching (Figure S22). The small background fluctuations observed are consistent with the higher background due to the use of coverslips rather than ZMW.

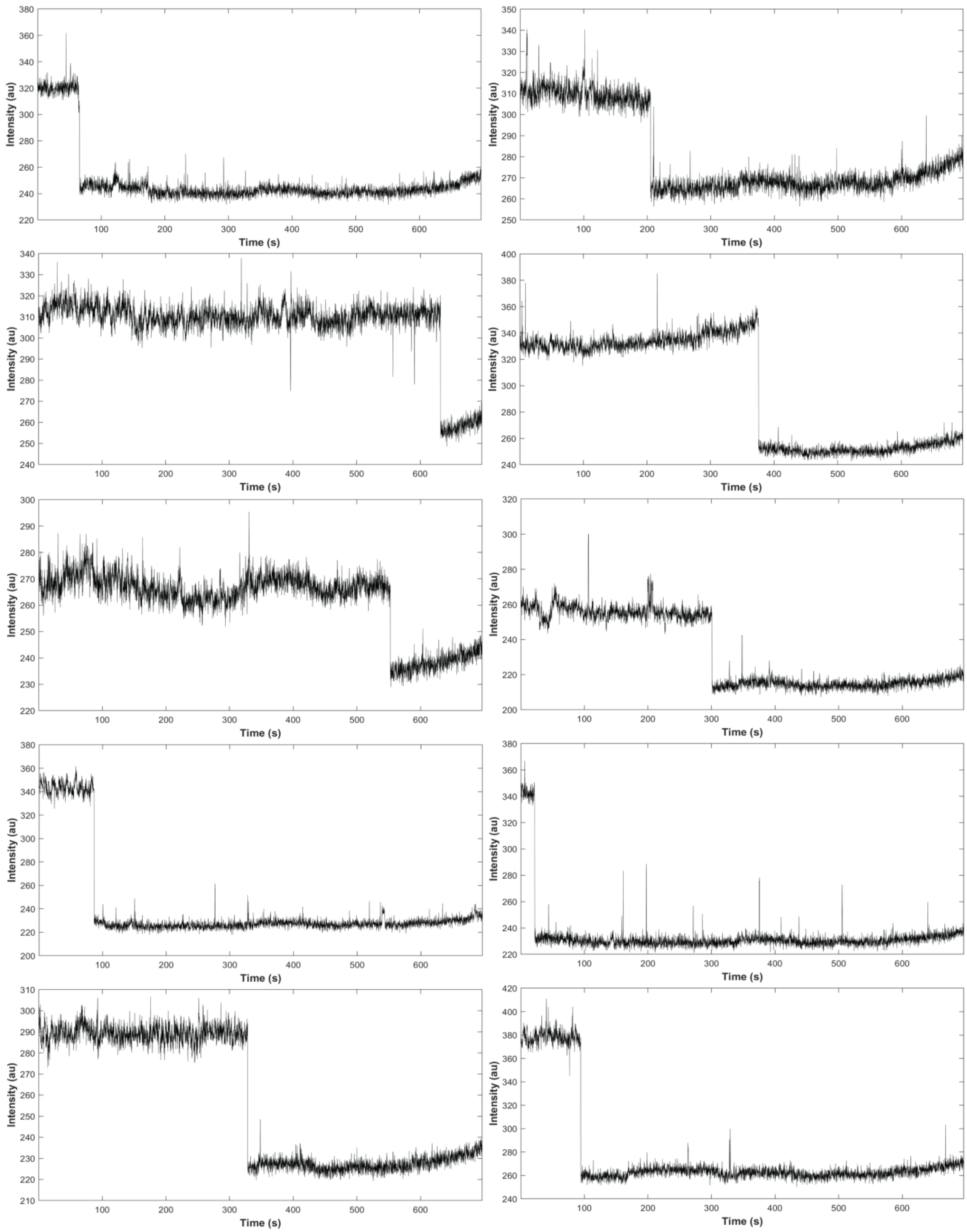

Figure S22. Representative traces from the blinking control experiment showing no large-scale blinking of **S1** is observed under the microscopy conditions used for rebinding experiments.

5. Supplemental Discussion

5.1 Differences in Observed Intensities

Differences in signal-to-background and fluorescence/background intensity magnitudes from molecule to molecule, even under the same microscopy conditions, are not atypical for single-molecule widefield fluorescence measurements. The excitation power experienced by each molecule will differ depending on the location of the molecule with respect to the beam center due to the gaussian shape of the expanded widefield beam. Thus, the location of the ZMW well with respect to the center of the excitation is primarily responsible for the observed differences in intensity magnitudes in Figure 3, Figure S4, Figure S5, Figure S19, Figure S20, and Figure S21, with catalysts in the center of the beam generally displaying higher signal-to-background and fluorescence intensity. However, fluorescence emission is also modulated in ZMWs based on the analyte's position within the well and proximity to the metal side walls.[15] The positioning of the catalyst within the ZMW well is not precisely controlled in this study and likely also contributes to the differences in intensity.

5.2 Effect of Confinement and Passivation on Kinetic Results

Due to the requirements of this study, catalyst **1** must be tethered to the bottom of a ZMW well along with a passivation layer. Given this change in environment compared to the homogeneous parent PEPPSI catalyst, it is reasonable to consider the effect of this confinement and passivation layer on the reported kinetic rates. Although we do not expect these rates to quantitatively match those of the homogeneous catalyst, we have reason to believe that they provide a reasonable approximation of in situ kinetics that, thus far, cannot and have not been measured via other means.

ZMWs have been used to measure diffusion coefficients of small molecules and have good agreement with bulk values,[16] suggesting that diffusion is not significantly impeded by the confinement within ZMW wells. Regarding effects of the passivation layer, we have previously studied the single-

molecule initiation of a tethered PEPPSI analog using a triethoxy(octyl)silane passivation layer on coverslips.[14] Past NMR experiments have shown qualitative agreement between the initiation times of the homogeneous catalyst and single-molecule data, with both proceeding in seconds,[14] indicating that the passivation does not substantially affect the kinetics of initiation. However, we do not rule out the possibility of catalyst interaction with the passivation layer and the potential for varied microenvironments affecting the distribution of dwell times, as discussed below.

5.3 Alternative Identification of States

Another mechanistic interpretation of Figure 5 originates in the surface-supported nature of the catalyst. There is variation in the local environment of the catalyst caused by heterogeneity in the surface sites of the silica surface.[17-21] As investigated previously,[14] the high triethoxy(octyl)silane (TEOS) deposition concentration may result in clusters of coalesced silane groups within the passivation layer, which were hypothesized to be responsible for observed static heterogeneity and had tangible effect on initiation dynamics. There may be mobility of the tethered catalyst species within the TEOS passivation layer,[22, 23] and it is possible that the catalyst can become less accessible to the pyridine binding partner when buried within the TEOS layer. We can then imagine each "arm" represents not a palladium oxidation state, but a different microenvironment inside the passivation layer, with a reversible transition between a more accessible catalyst and a less accessible buried catalyst leading to two different dark states and two corresponding bright states. With slower rates of ligand binding and unbinding, the right "arm" would represent a deeper, less accessible conformation. This interpretation offers several features that make it a plausible fit for the scheme in Figure 5. The deposition of the catalyst and the TEOS passivation is identical between experiments with and without coupling partners, so the pockets in the TEOS should be the same regardless of the presence or absence of the coupling partners, consistent with the largely unchanged kinetics in the "arms" of the kinetic scheme. When bound to the catalyst species, the large size of BODIPY labeled pyridine ligand **2** could prevent significant movement though the TEOS layer, explaining why

interconversion between the liganded states is slow or absent. The steric influence of the passivation layer is likely also at least partially responsible for the large population of catalyst molecules, as identified from the presence of the green initial ligand, that do not show ligand rebinding.

However, difficulties arise when explaining the role of the coupling partners in altering the equilibrium: why would access to the catalytic cycle result in a different equilibrium constant between the more and less accessible configuration? One hypothesis is that the presence of other ligands on the metal, possibly due to a shift in the catalyst resting state from a less liganded species (without coupling partners) to a more liganded species (with coupling partners), makes it more sterically difficult for the catalyst to burrow more deeply into the passivation layer, resulting in a slower rate of transition from the left unliganded state to the right. However, once in the less accessible conformation, the process of reemerging might be gated by a TEOS molecule, leaving the reverse rate unchanged. Overall, the shifting of the equilibrium constant away from the less ligand accessible state is consistent with this picture. Still, this model seemed a less natural fit than the one proposed in the main text, but could not be completely ruled out.

5.4 Summary of some important prior work in single-molecule catalysis

In enzymes, single-molecule studies have successfully observed enzymatic turnover in real-time,[24, 25] investigated how enzyme conformation changes during reaction,[26] and observed single redox events.[10] Single-particle studies on nanoparticle and heterogeneous catalysts have probed surface intermediates,[27, 28] revealed differences in reactivity between different catalyst particles,[27, 29] mapped active sites,[30-35] investigated how nanoconfinement affects catalytic activity,[36] determined if the active site of a nanoparticle catalyst is the solid surface or leached solution-phase particles or ions,[37] visualized ion transport from individual battery cathode mesoparticles,[38] and measured turnover frequency.[39] In molecular organometallic catalysts, single-molecule fluorescence has been used to study catalyst

initiation,[14] track the 3D growth of polymer chains,[40] uncover chemoselectivity in polymer formation,[41] and study the kinetics of polymer growth.[42, 43] Application of single-molecule fluorescence to organocatalysts and organic reactions have allowed direct observation of product formation at the single-molecule level,[44] detection of low concentration intermediates.[45] real-time monitoring of changes in chirality over the course of reaction.[46] Despite these advances in the field, there is a noticeable lack of single-molecule methods for studying catalysis of small molecule reactions at synthetically relevant concentrations, which is crucial for gaining representative mechanistic information.

6. Supplemental Synthetic Protocols and Characterization

General Considerations

Unless otherwise noted, all reagents were used as obtained from the vendor without further purification and all glassware was oven-dried overnight at 200°C. Reactions were carried out under a dry nitrogen atmosphere using Schlenk techniques unless otherwise noted. Tetrahydrofuran and dichloromethane were taken from a Glass Contour Solvent System by Pure Process Technology, LLC under argon. Reactions were monitored by thin-layer chromatography on pre-coated glass-backed plates and were visualized using UV light. Flash chromatography was performed on silica gel from Sigma-Aldrich. ^{1}H and ^{13}C NMR spectra were acquired on a Bruker Avance 400 or 500 MHz spectrometer. UV-vis spectra were recorded on a Varian Cary 50 Scan UV-visible Spectrometer. High resolution mass spectra were recorded on a Thermo Q Exactive Plus. Plasma cleaning was done in a Harrick Plasma PDC-001-HP using atmospheric oxygen.

Synthetic Procedures

Scheme S1: Synthesis of the catalyst dimer precursor.

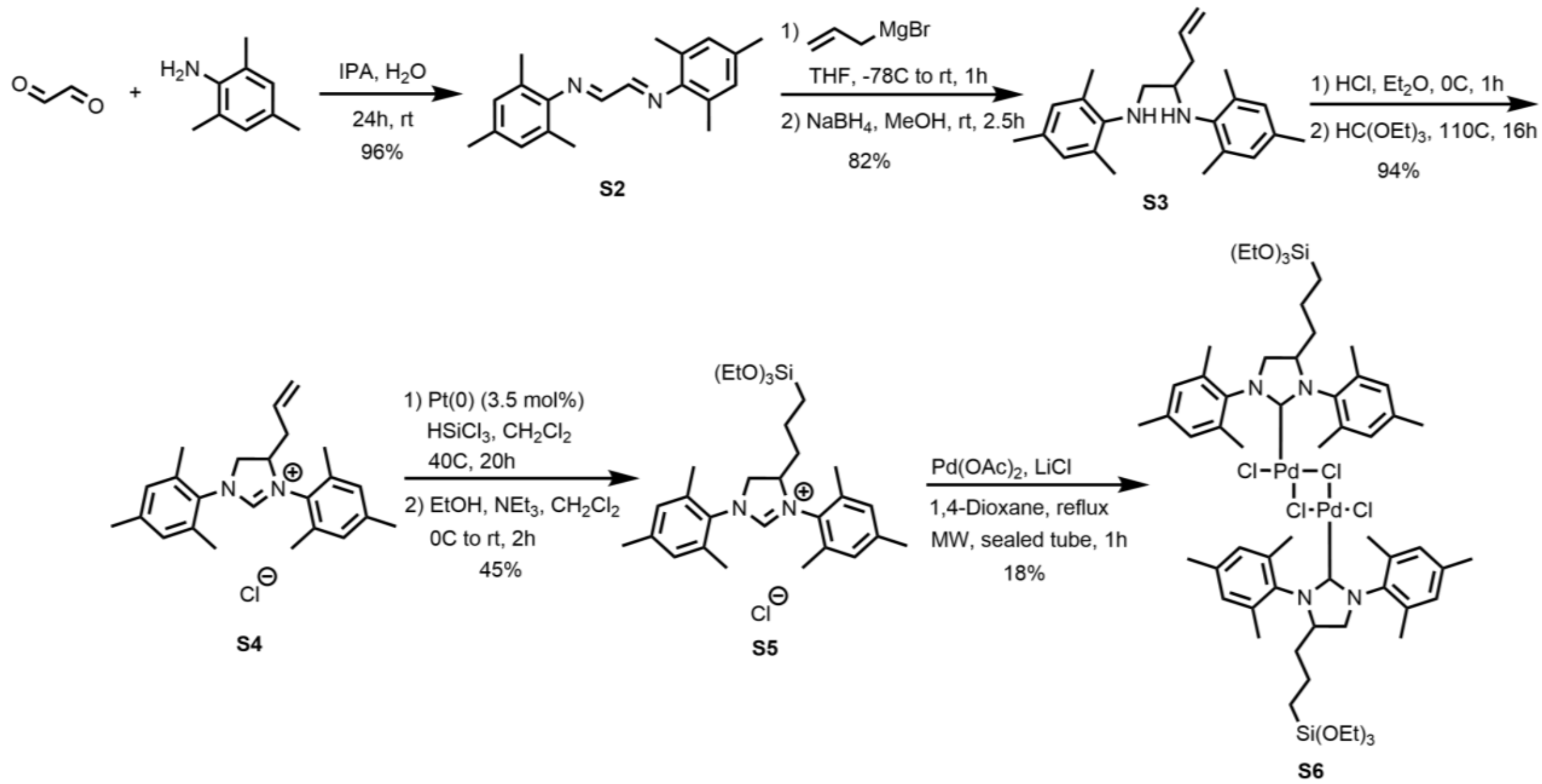

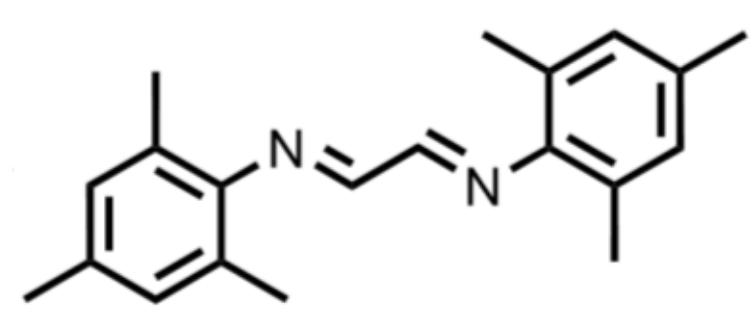

S2

2,4,6-trimethylaniline (10.4 mL, 74 mmol) was dissolved in 40 mL IPA. A solution of 40% glyoxal in water (5.24 mL, 37 mmol) was dissolved in 15 mL IPA and 15 mL DI water. The solutions were combined into a 250 mL round bottom flask and stirred for 24 hours. The yellow precipitate was filtered through a Buchner funnel and washed with DI water before drying in air overnight. 10.061 g product isolated (96% yield) verified by ^{1}H NMR.

^{1}H NMR (400 MHz, $CDCl_3$) δ 8.10 (s, 2H), 6.91 (s, 4H), 2.29 (s, 6H), 2.16 (s, 12H).

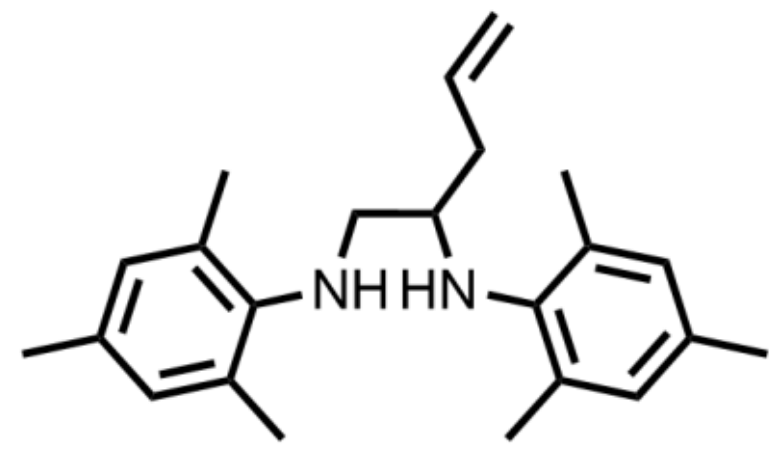

S3

The literature procedure was followed.[47] **S2** (2.0 g, 6.9 mmol) was dissolved in 200 mL dry THF in an oven dried 1000 mL round bottom flask and cooled to -78°C. Allylmagnesium bromide (6.92 mL, 1 M in ether, 6.92 mmol) was added dropwise. The solution was allowed to warm to room temperature and stirred for one hour. The reaction was diluted with 160 mL methanol and sodium borohydride (1.60 g, 42.3 mmol) was added in two portions with 30 minutes between additions. The mixture was then stirred for 2 hours following the second addition. The reaction was quenched with saturated NH_4Cl until bubbling ceased, then the organic layer was extracted with ethyl acetate and the aqueous layer was washed with ethyl acetate (3 x 50 mL). The combined organic extracts were washed with brine and dried over $MgSO_4$. The product was purified via column chromatography (5% EtOAc/hexanes, 5 mL TEA/1L eluent) to give 1.896 g product (82% yield) verified by ^1H NMR.

^1H NMR (400 MHz, C_6D_6) δ 6.80 (s, 2H), 6.79 (s, 2H), 5.70 – 5.58 (m, 1H), 4.98 – 4.90 (m, 2H), 3.53 (tt, *J* = 8.4, 4.7 Hz, 1H), 3.33 (br s, 3H), 3.17 (dd, *J* = 12.0, 4.4 Hz, 4H), 2.72 (dd, *J* = 12.0, 6.7 Hz, 1H), 2.23 (s, 6H), 2.20 (s, 9H), 2.18 (s, 3H), 2.00 (dt, *J* = 13.8, 8.0 Hz, 1H).

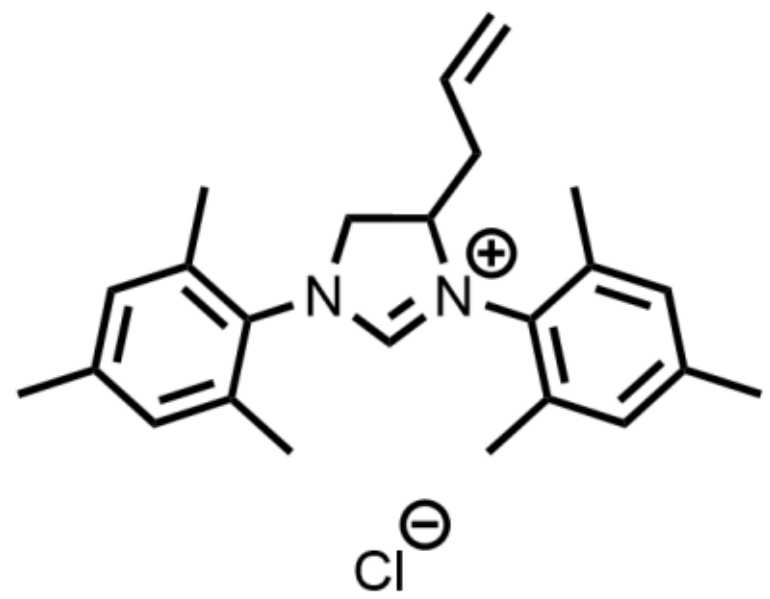

S4

The literature procedure was followed.[47] **S3** (1.55 g, 4.61 mmol) was dissolved in 25 mL ether in an oven dried 100 mL round bottom flask and cooled to 0°C. HCl (2.75 mL, 2 M in ether, 5.5 mmol) was added dropwise and the solution was warmed to room temperature and let stir for one hour. The resulting precipitate was isolated by filtration, washed with ether, and briefly dried under reduced pressure before being transferred to an oven dried 100 mL round bottom flask. Triethyl orthoformate (10 mL, 60.2 mmol) was added and the mixture was stirred at 110°C for 16 hours. Volatiles were removed under vacuum and the product and washed with ether and hexane to give 1.526 g product (94% yield over two steps) verified by ^{1}H NMR.

^{1}H NMR (400 MHz, CD_2Cl_2) δ 10.60 (s, 1H), 7.04 (d, *J* = 6.7 Hz, 4H), 5.68 – 5.52 (m, 1H), 5.24 – 5.15 (m, 2H), 4.86 – 4.75 (m, 1H), 4.45 (t, *J* = 11.7 Hz, 1H), 3.96 (dd, *J* = 12.1, 8.4 Hz, 1H), 2.63 – 2.47 (m, 2H), 2.45 (s, 3H), 2.43 (s, 3H), 2.40 (s, 6H), 2.33 (d, *J* = 2.6 Hz, 6H), 1.71 (s, 3H).

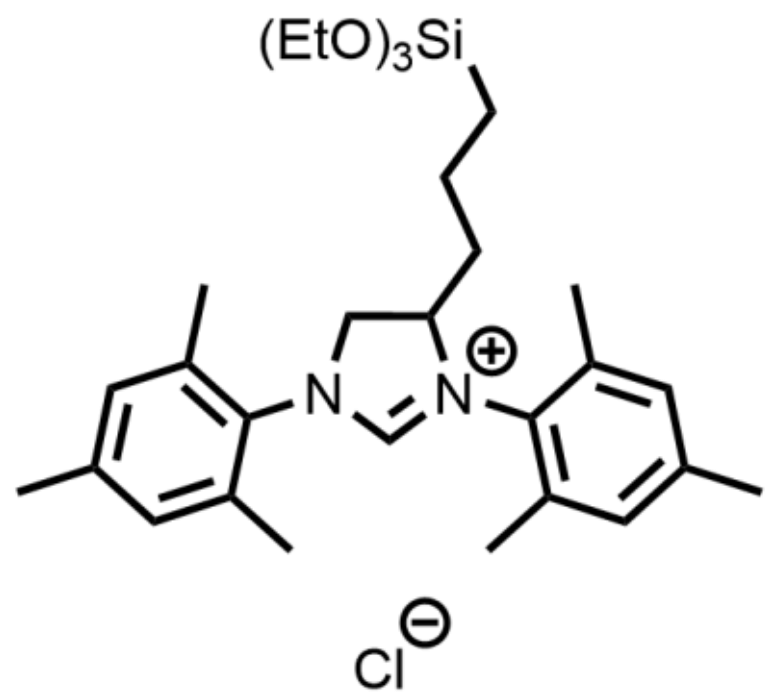

S5

The literature procedure was followed.[47] **S4** (500 mg, 1.31 mmol) was dissolved in 10 mL dry DCM. Trichlorosilane (5 mL, 6.7 g, 49.5 mmol) was added to the reaction mixture followed by Pt(0)-1,3-divinyl1,1,3,3-tetramethyldisiloxane (Karstedt's catalyst, 500 µl of a 0.10 M solution in xylenes, 0.050 mmol, 3.8 mol%). The mixture was allowed to stir at reflux for 20 hours. Excess trichlorosilane was removed under vacuum and collected in a secondary cold trap. The resulting residue was dissolved in 10 mL dry DCM and cooled to 0°C. A 5 mL solution of 1:1 ethanol and TEA was added dropwise and the mixture was warmed to room temperature and allowed to stir for 2 hours. The product was purified via column chromatography (5% EtOAc/DCM) to give 355.4 mg product (45% yield) verified by ^{1}H NMR.

^{1}H NMR (500 MHz, CD_2Cl_2) δ 10.46 (s, 1H), 7.04 (d, *J* = 7.3 Hz, 4H), 4.67 (ddd, *J* = 11.4, 5.2, 2.5 Hz, 1H), 4.46 (t, *J* = 11.5 Hz, 1H), 3.91 (dd, *J* = 11.8, 9.0 Hz, 1H), 3.72 (q, *J* = 7.0 Hz, 6H), 2.44 (s, 3H), 2.42 (s, 3H), 2.39 (s, 3H), 2.37 (s, 3H), 2.33 (s, 6H), 1.84 – 1.79 (m, 2H), 1.47 – 1.24 (m, 2H), 1.15 – 1.12 (m, 9H), 0.60 – 0.55 (m, 2H).

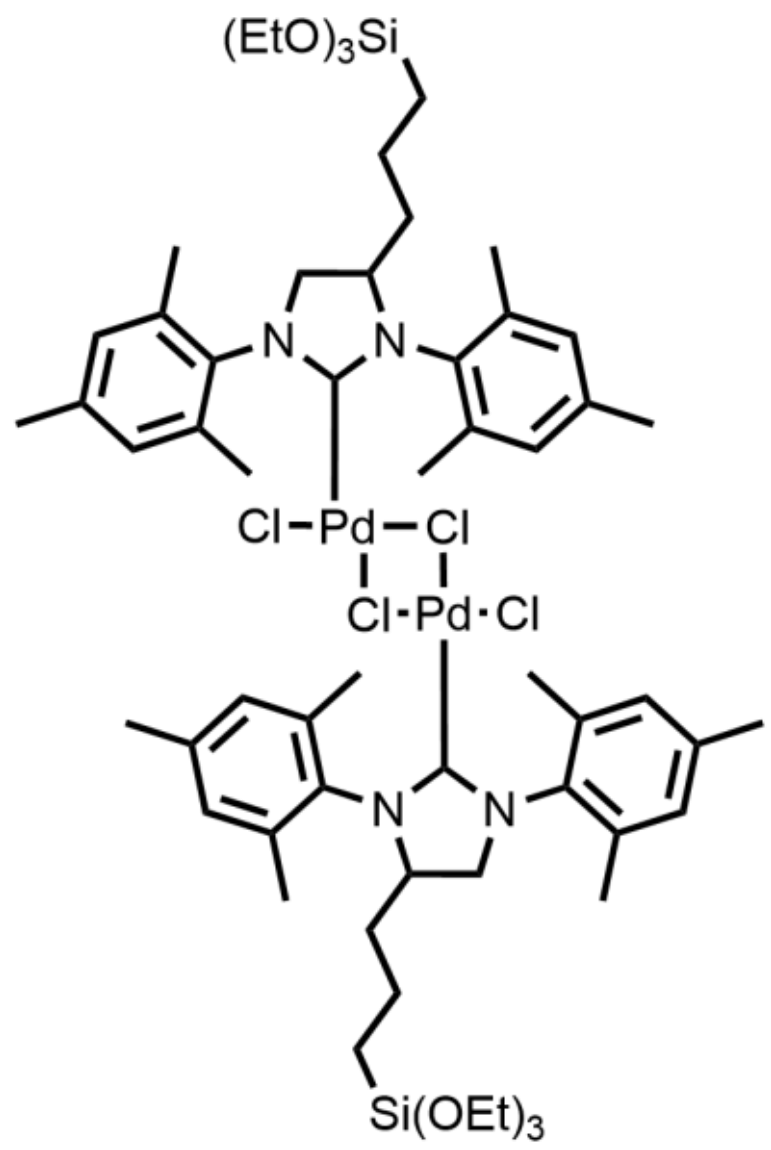

S6

The literature procedure was followed.[1] LiCl (579.3 mg, 13.69 mmol, dried on vacuum manifold at 140°C overnight) and palladium (II) acetate (154 mg, 0.68 mmol) were added to an oven dried 10 mL microwave vial. **S5** was dissolved in 3.5 mL 1,4-dioxane and added to the microwave vial. The mixture was allowed to stir at 100°C for one hour in a CEM Discover microwave reactor (300 W). The solution was then filtered through a Buchner funnel and washed with DCM to remove palladium black. The product was purified via column chromatography (40% EtOAc/hexanes) to give 82.3 mg product (18% yield) verified by ^{1}H NMR.

^{1}H NMR (500 MHz, CD_2Cl_2) δ 7.04 (d, *J* = 10.4 Hz, 4H), 6.98 (d, *J* = 4.2 Hz, 4H), 4.18 (dd, *J* = 8.6, 5.0 Hz, 2H), 4.02 – 3.90 (m, 2H), 3.71 (q, *J* = 6.9 Hz, 12H), 3.63 (dd, *J* = 10.7, 7.8 Hz, 2H), 2.58 (s, 5H), 2.52 (s, 6H), 2.45 (s, 6H), 2.40 (s, 6H), 2.35 (s, 12H), 1.64 (m, 4H), 1.30 (m, 5H), 1.12 (dt, *J* = 10.9, 7.0 Hz, 21H), 0.55 – 0.45 (m, 5H).

Scheme S2: Synthesis of red and green BODIPY labeled pyridine ligands.

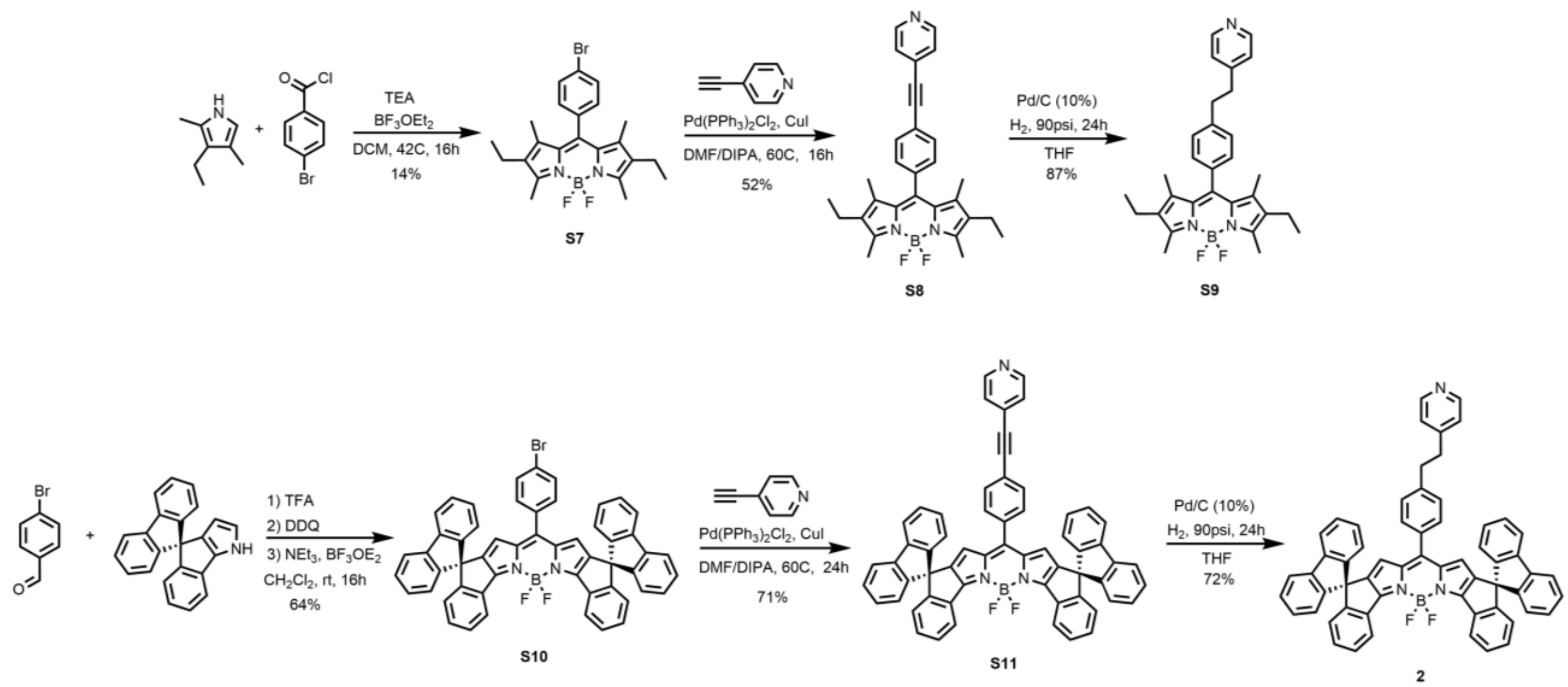

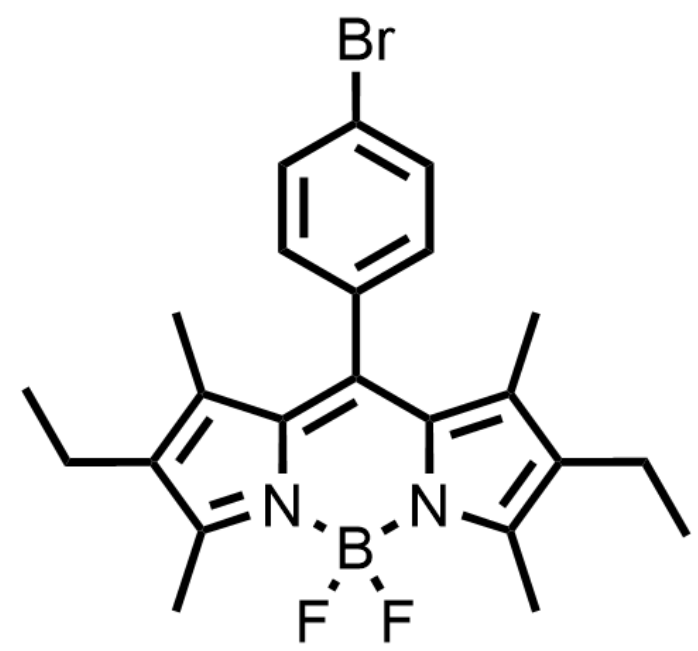

S7

A solution of 3-ethyl-2,4-dimethyl pyrrole (680 µL, 5.04 mmol) and 4-bromobenzoyl chloride (526.7 mg, 2.4 mmol) in 60 mL dry DCM was prepared in a dry 250 mL round bottom flask and heated to 42°C for 16 hours. Let come to room temperature before adding 2.0 mL TEA followed by 4.0 mL 47% BF_3OEt_2. The mixture was allowed to stir for 3 hours. The solution was extracted with 50 mL saturated NH_4Cl and the aqueous layer was washed with DCM (3 x 10 mL). The organic layers were combined and dried over Na_2SO_4. The product was purified via column chromatography (10% EtOAc/hexanes) to give 148.1 mg product (14% yield) verified by ^1H NMR.

^1H NMR (400 MHz, $CDCl_3$) δ 7.63 (d, J = 8.3 Hz, 2H), 7.18 (d, J = 8.4 Hz, 2H), 2.53 (s, 6H), 2.30 (q, J = 7.5 Hz, 4H), 1.31 (s, 6H), 0.98 (t, J = 7.6 Hz, 6H).

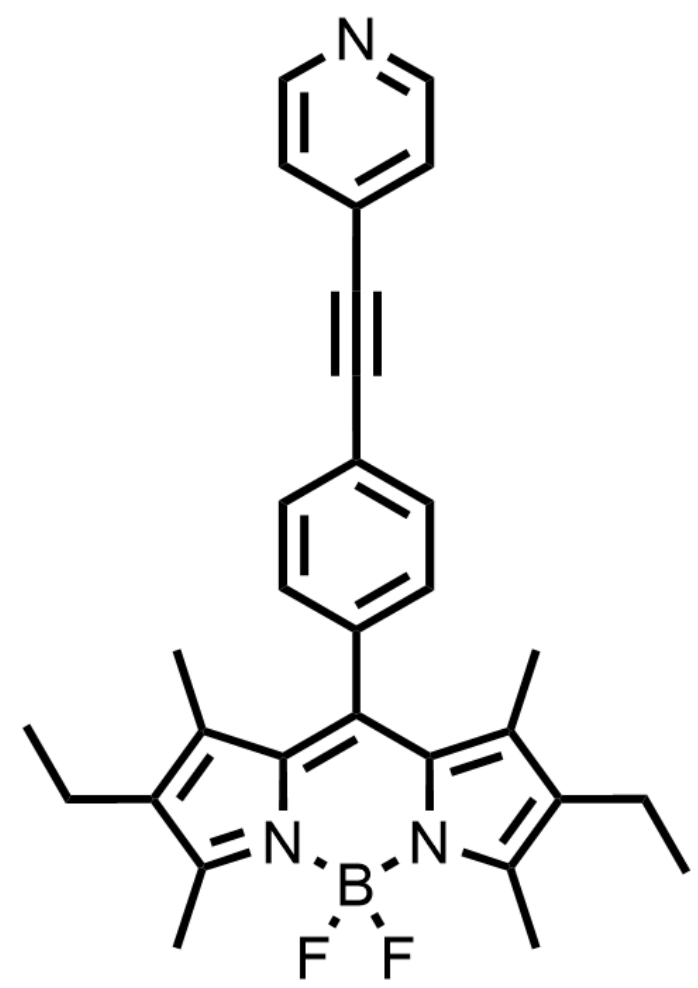

S8

The literature procedure was followed.[1, 14] An oven dried 50 mL single-neck round bottomed flask was charged with 4-ethynylpyridine (28 mg, 0.268 mmol), **S7** (112 mg, 0.242 mmol), $Pd(PPh_3)_2Cl_2$ (16 mg, 0.0242 mmol), CuI (8 mg, 0.0484 mmol), and 24 mL of Argon purged diisopropylamine (DIPA):DMF (1:2) solution. This reaction flask was then heated to 60°C and allowed to stir for 16 hours. The reaction was quenched with NH_4Cl and extracted with ethyl acetate (3 x 50 mL). The combined ethyl acetate extracts were washed with brine (50 mL), dried over Na_2SO_4. The product was purified via column chromatography (50% DCM/EtOAc) to give 117.9 mg product (52% yield) verified by ^{1}H NMR.

^{1}H NMR (400 MHz, $CDCl_3$) δ 8.64 (d, *J* = 6.1 Hz, 2H), 7.69 (d, *J* = 8.2 Hz, 2H), 7.42 (d, *J* = 6.0 Hz, 2H), 7.33 (d, *J* = 8.2 Hz, 2H), 2.54 (s, 6H), 2.31 (q, *J* = 7.5 Hz, 4H), 1.32 (s, 6H), 0.99 (t, *J* = 7.5 Hz, 6H).

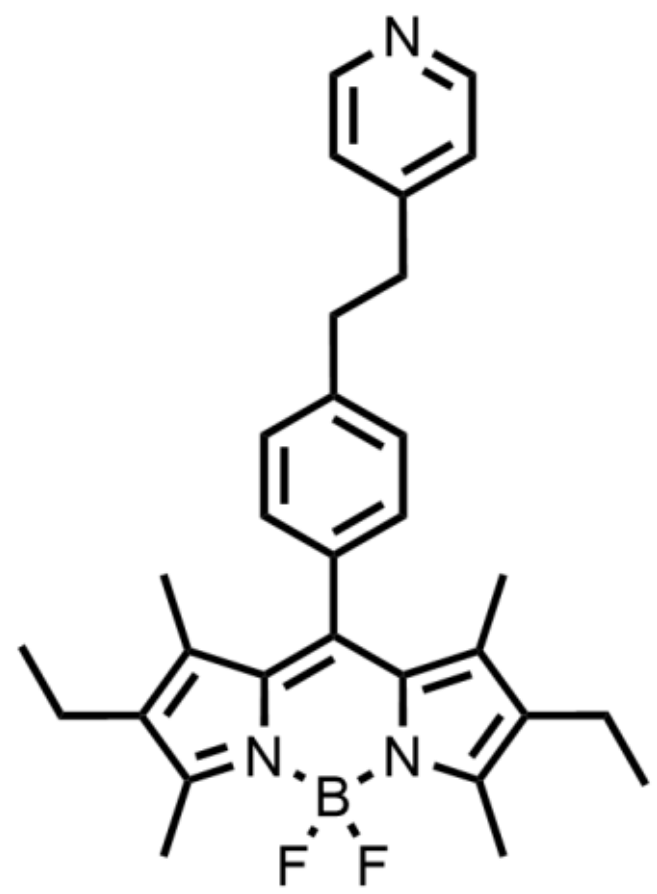

S9

The literature procedure was followed.[1, 14] The glass reaction chamber of a Parr reactor was oven dried and charged with **S8** (60 mg, 0.128 mmol) and Pd/C (10%, 6.0 mg, 10Wt%) in 5 mL dry THF. The Parr reactor was pressurized with hydrogen and evacuated three times, then maintained at 90 psi for 6 hours. Additional Pd/C (10%) (~2 mg) was added, the reactor was pressurized with hydrogen and evacuated three times, then maintained at 90 psi for 18 hours while being monitored by TLC, which showed an absence of starting material. Reaction was carefully filtered, washed with THF (50-100 mL) and DCM (100 mL). Leftover Pd/C was poured in cold water and decomposed. The product was purified via column chromatography (1:1 EtOAc:hexanes) to give 53.8 mg product (87% yield) verified by ^{1}H NMR.

^{1}H NMR (500 MHz, $CDCl_3$) δ 8.46 (d, *J* = 6.0 Hz, 2H), 7.17 (q, *J* = 8.2 Hz, 4H), 7.02 (d, *J* = 6.1 Hz, 2H), 3.01 (dd, *J* = 19.9, 6.7 Hz, 4H), 2.52 (s, 6H), 2.30 (q, *J* = 7.5 Hz, 4H), 1.26 (s, 6H), 0.98 (t, *J* = 7.6 Hz, 6H).

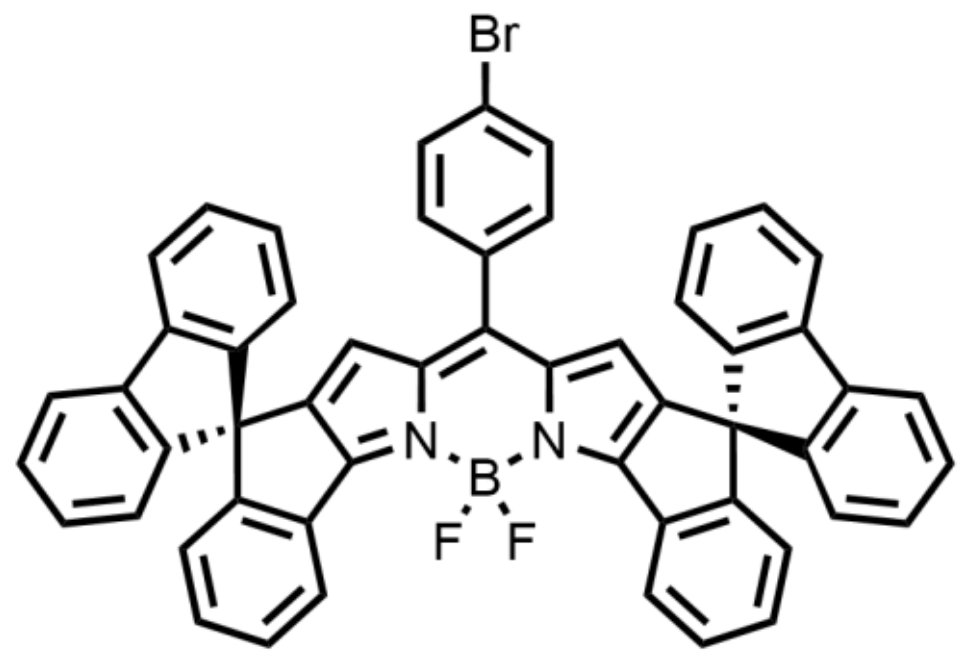

S10

The literature procedure was followed.[6, 14] A solution of spiro[fluorene-9,4'(1'H)-indeno[1,2-b]pyrrole (363.7 mg, 1.19 mmol)[6] and 4-bromobenzaldehyde (104.9 mg, 0.567 mmol) in 17 mL dry DCM was prepared in a dry 100 mL round bottom flask. Two drops of TFA were added and the mixture was stirred under nitrogen overnight. The following day, DDQ (141.7 mg, 0.624 mmol) was added along with an additional 17 mL of dry DCM. After stirring for 1 hour, 500 µL TEA was added, followed by 1 mL 47% BF_3OEt_2. This mixture was allowed to stir for 3 hours, then was quenched with 30 mL saturated $NaHCO_3$ and extracted with DCM (3 x 10 mL). The organic layers were combined, then extracted with 30 mL brine twice, washing the aqueous layer with DCM (3 x 10 mL) each time. The organic layers were combined and dried over $MgSO_4$. The product was purified via column chromatography (30% hexanes/$CHCl_3$) to give 289.7 mg product (64% yield) verified by ^{1}H NMR.

^{1}H NMR (400 MHz, $CDCl_3$) δ 8.49 (d, *J* = 7.8 Hz, 2H), 7.80 (d, *J* = 7.6 Hz, 4H), 7.52 (t, *J* = 7.6 Hz, 2H), 7.41 – 7.33 (m, 6H), 7.23 (d, *J* = 8.3 Hz, 4H), 7.17 (t, *J* = 7.5 Hz, 4H), 6.95 (d, *J* = 7.6 Hz, 4H), 6.65 (d, *J* = 7.7 Hz, 2H), 6.15 (s, 2H).

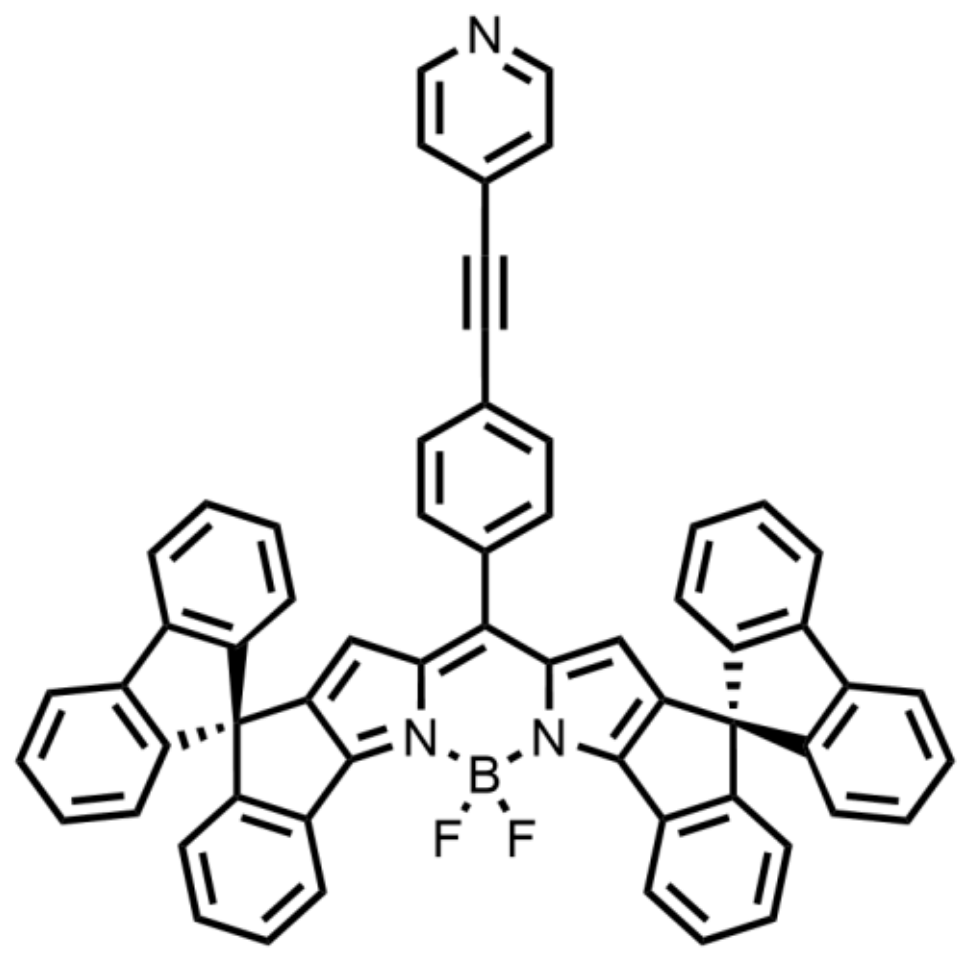

S11

The literature procedure was followed.[1, 14] An oven dried 25 mL single-neck round bottomed flask with stir bar and reflux condenser was charged with 4-ethynylpyridine (14 mg, 0.134 mmol), **S10** (100 mg, 0.121 mmol), $Pd(PPh_3)_2Cl_2$ (8 mg, 0.0121 mmol), CuI (4 mg, 0.0242 mmol), and 12 mL of Argon purged diisopropylamine (DIPA):DMF (1:2) solution. This reaction flask was then heated to 60°C and allowed to stir for 24 hours. The reaction was quenched with NH_4Cl and extracted with ethyl acetate (3 x 50 mL). The combined ethyl acetate extracts were washed with brine (50 mL) and dried over Na_2SO_4. The product was purified via column chromatography (25% EtOAc/DCM with 0.5 mL TEA/1 L eluent) to give 71.8 mg product (71% yield) verified by ^{1}H NMR.

^{1}H NMR (500 MHz, $CDCl_3$) δ 8.57 (d, *J* = 6.1 Hz, 2H), 8.50 (d, *J* = 7.7 Hz, 2H), 7.80 (d, *J* = 7.6 Hz, 4H), 7.52 (td, *J* = 7.6, 1.1 Hz, 2H), 7.42 (d, *J* = 8.5 Hz, 2H), 7.40 – 7.35 (m, 6H), 7.32 –7.30 (m, 2H), 7.22 (td, *J* = 7.6, 1.1 Hz, 2H), 7.18 (td, *J* = 7.5, 1.1 Hz, 4H), 6.96 (d, *J* = 7.6 Hz, 4H), 6.65 (dt, *J* = 7.6, 0.9 Hz, 2H), 6.18 (s, 2H).

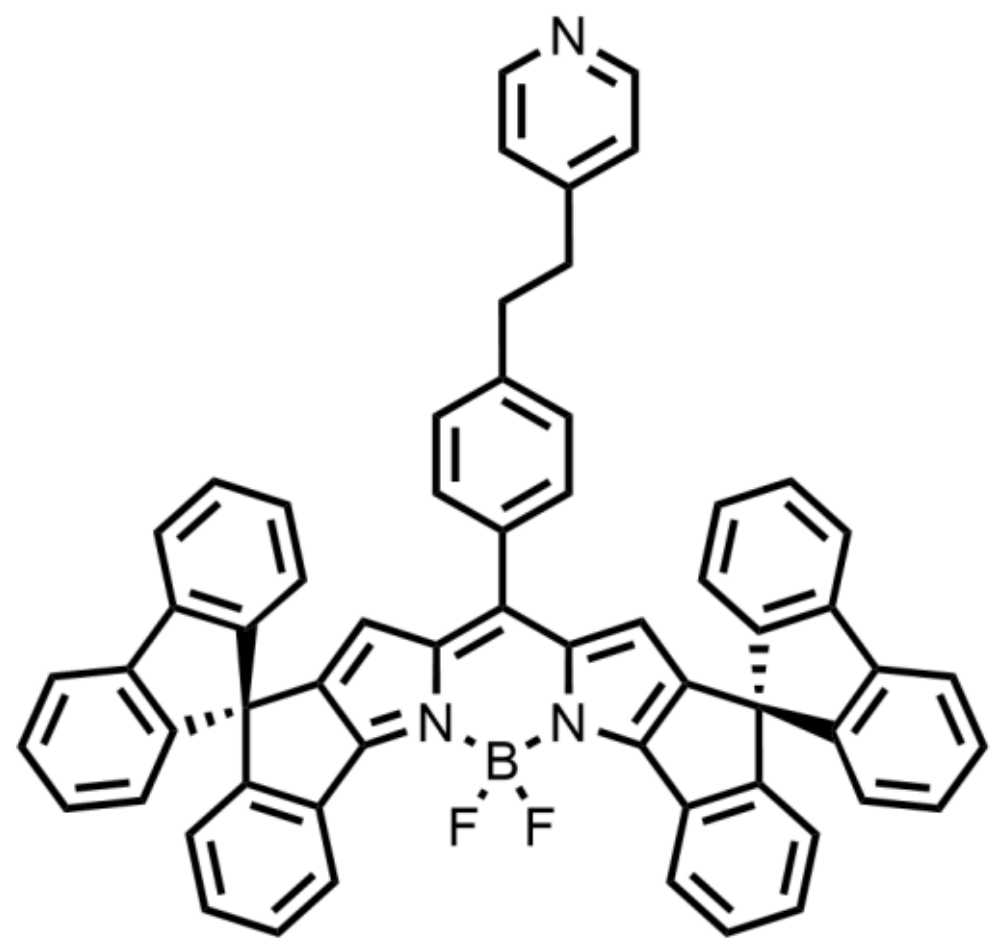

2

The literature procedure was followed.[1, 14] The glass reaction chamber of a Parr reactor was oven dried and charged with **S11** (36 mg, 0.0426 mmol) and Pd/C (10%, 3.6 mg, 10Wt%) in 5 mL dry THF. The Parr reactor was pressurized with hydrogen and evacuated three times, then maintained at 90 psi for 24 hours while being monitored by TLC, which showed an absence of starting material. Reaction was carefully filtered, washed with THF (50-100 mL) and DCM (100 mL). Leftover Pd/C was poured in cold water and decomposed. The product was purified via column chromatography (1:1:2 EtOAc:THF:DCM with 0.5 mL TEA/L eluent) to give 22.9 mg product (72% yield) verified by ^{1}H NMR.

^{1}H NMR (400 MHz, $CDCl_3$) δ 8.49 (d, J = 7.7 Hz, 2H), 8.45 – 8.41 (m, 2H), 7.80 (d, J = 7.6 Hz, 4H), 7.51 (t, J = 7.6 Hz, 2H), 7.37 (td, J = 7.5, 1.1 Hz, 4H), 7.29 (d, J = 8.0 Hz, 2H), 7.24 – 7.15 (m, 6H), 7.07 (d, J = 8.1 Hz, 2H), 7.05 (d, J = 5.9 Hz, 2H), 6.96 (d, J = 7.6 Hz, 4H), 6.64 (d, J = 7.7 Hz, 2H), 6.19 (s, 2H), 2.82 (d, J = 3.0 Hz, 4H).

Scheme S3: Final labeled catalyst synthesis.

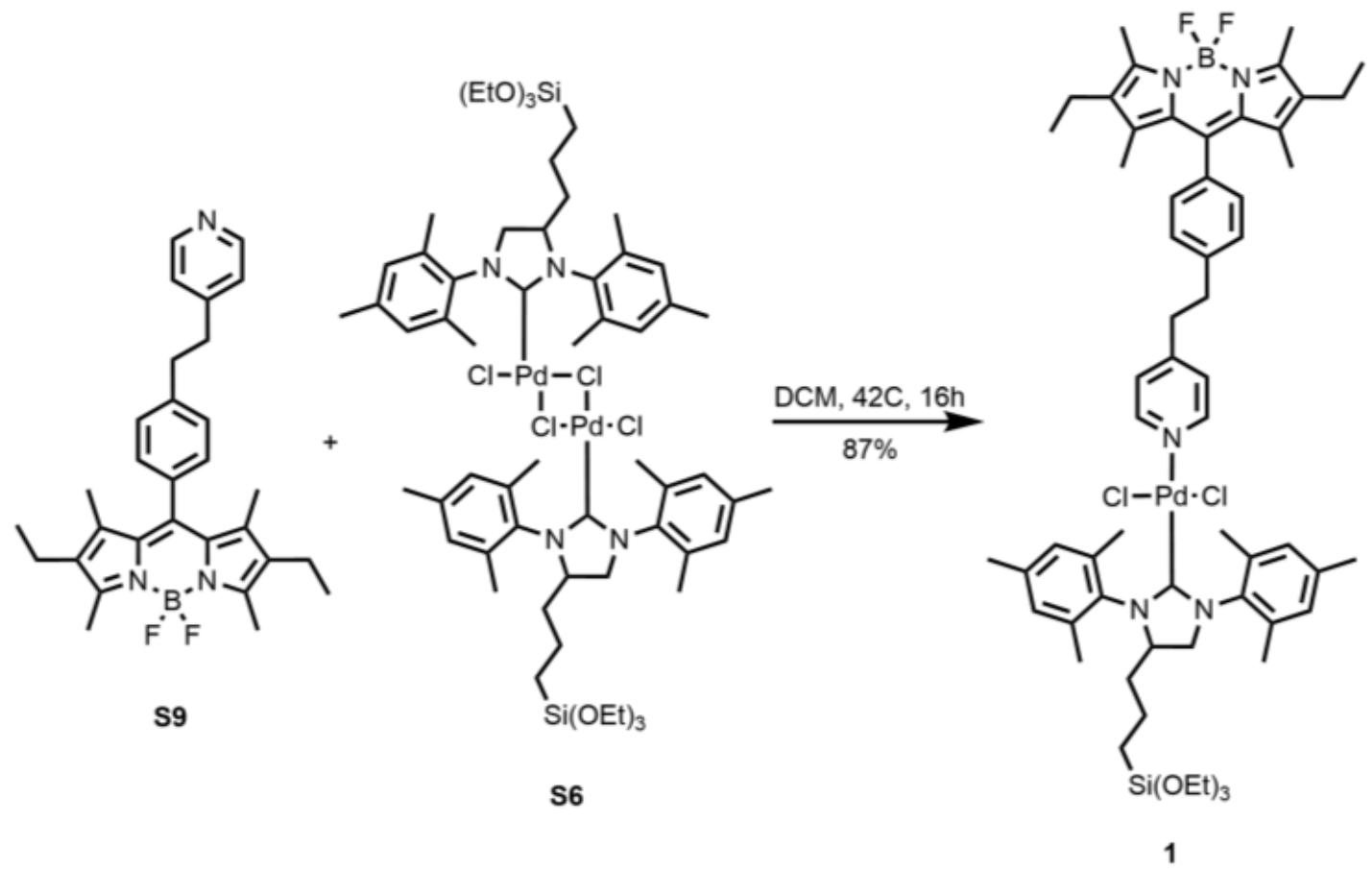

The literature procedure was followed.[1, 14] An oven dried 25 mL round bottomed flask was charged with **S9** (23 mg, 0.047 mmol) and dissolved in 11.76 mL dry DCM. **S5** (22 mg, 0.016 mmol) was dissolved in 0.24 mL dry DCM and added to the reaction flask. The reaction flask was then heated to 42°C for 16 hours while being monitored by TLC, which showed an absence of **S6**. The product was purified via column chromatography (1:1 EtOAc:hexane) to give 32.6 mg (87% yield) verified by ^{1}H NMR, ^{13}C NMR, and mass spectrometry.

^{1}H NMR (500 MHz, $CDCl_3$) δ 8.29 (d, *J* = 6.6 Hz, 2H), 7.20 – 7.12 (m, 4H), 6.99 (dd, *J* = 20.3, 8.6 Hz, 4H), 6.86 (d, *J* = 6.7 Hz, 2H), 4.22 (m, 1H), 4.06 (t, *J* = 10.7 Hz, 1H), 3.75 (q, *J* = 7.0 Hz, 7H), 3.69 (dd, *J* = 10.7, 7.3 Hz, 1H), 2.84 (m, 4H), 2.68 (s, 3H), 2.61 (s, 3H), 2.53 (d, *J* = 4.4 Hz, 9H), 2.48 (s, 3H), 2.32 (s, 6H), 2.29 (q, *J* = 7.5 Hz, 4H), 1.71 (m, 2H), 1.23 (s, 6H), 1.17 (td, *J* = 7.0, 3.6 Hz, 11H), 0.98 (t, *J* = 7.5 Hz, 6H), 0.57 (ddd, *J* = 9.4, 6.4, 4.5 Hz, 2H).

^{13}C NMR (500 MHz, $CDCl_3$) δ 183.99, 153.77, 152.14, 151.12, 141.12, 138.49, 138.15, 137.58, 137.23, 135.12, 134.22, 134.02, 132.88, 130.97, 129.88, 129.71, 129.60, 129.13, 128.62, 124.16, 64.14, 58.54, 56.50, 36.97, 36.25, 21.27, 21.02, 19.95, 19.71, 19.60, 19.53, 18.42, 17.22, 14.77, 12.64, 11.89, 10.52, 0.14.

ESI(M+Na)+: calculated 1195.4474; found 1195.4421.

7. Supplemental References

(1) Upadhyay, S. P.; Lupo, K. M.; Marquard, A. N.; Ng, J. D.; Bates, D. M.; Goldsmith, R. H. Fluorescent Dendrimeric Molecular Catalysts Demonstrate Unusual Scaling Behavior at the Single-Molecule Level. *J. Phys. Chem. C* **2015**, *119* (34), 19703-19714. DOI: 10.1021/acs.jpcc.5b06562.
(2) Hinton, D. A.; Ng, J. D.; Sun, J.; Lee, S.; Saikin, S. K.; Logsdon, J.; White, D. S.; Marquard, A. N.; Cavell, A. C.; Krasecki, V. K.; Knapper, K. A.; Lupo, K. M.; Wasielewski, M. R.; Aspuru-Guzik, A.; Biteen, J. S.; Gopalan, P.; Goldsmith, R. H. Mapping Forbidden Emission to Structure in Self-Assembled Organic Nanoparticles. *J Am Chem Soc* **2018**, *140* (46), 15827-15841. DOI: 10.1021/jacs.8b09149.
(3) White, D. S.; Goldschen-Ohm, M. P.; Goldsmith, R. H.; Chanda, B. Top-down machine learning approach for high-throughput single-molecule analysis. *eLife* **2020**, *9*, e53357. DOI: 10.7554/eLife.53357 (acccessed 2024/12/14/21:42:52).
(4) Bandyopadhyay, A.; Goldschen-Ohm, M. P. Unsupervised selection of optimal single-molecule time series idealization criterion. *Biophysical Journal* **2021**, *120* (20), 4472-4483. DOI: 10.1016/j.bpj.2021.08.045 (acccessed 2024/12/14/21:42:22).
(5) White, D. S.; Dunyak, B. M.; Vaillancourt, F. H.; Hoskins, A. A. A sequential binding mechanism for 5′ splice site recognition and modulation for the human U1 snRNP. *Nat Commun* **2024**, *15*, 8776. DOI: 10.1038/s41467-024-53124-5 (acccessed 2025/02/03/04:07:28).
(6) Kowada, T.; Yamaguchi, S.; Ohe, K. Highly Fluorescent BODIPY Dyes Modulated with Spirofluorene Moieties. *Org. Lett.* **2010**, *12* (2), 296-299. DOI: 10.1021/ol902631d (acccessed 2025/09/02/19:34:55).
(7) Ho, J. Predicting pKa in Implicit Solvents: Current Status and Future Directions*. *Australian Journal of Chemistry* **2014**, *67* (10), 1441-1460. DOI: 10.1071/ch14040.
(8) Perrin, D. D.; Pure, I. U. o.; Chemistry, A. C. C. o. E. *Dissociation Constants of Organic Bases in Aqueous Solution: Supplement 1972*; Butterworths, 1972.
(9) Blanco, M.; Walter, N. G. Analysis of complex single-molecule FRET time trajectories. In *Methods in enzymology*, Vol. 472; Elsevier, 2010; pp 153-178.
(10) Goldsmith, R. H.; Tabares, L. C.; Kostrcz, D.; Dennison, C.; Aartsma, T. J.; Canters, G. W.; Moerner, W. E. Redox cycling and kinetic analysis of single molecules of solution-phase nitrite reductase. *P Natl Acad Sci USA* **2011**, *108* (42), 17269-17274. DOI: DOI:10.1073/pnas.1113572108.
(11) Xie, X. S. Single-molecule approach to enzymology. *Single Mol* **2001**, *2* (4), 229-236.
(12) Yang, S. K.; Shi, X.; Park, S.; Ha, T.; Zimmerman, S. C. A dendritic single-molecule fluorescent probe that is monovalent, photostable and minimally blinking. *Nature Chemistry* **2013**, *5* (8), 692-697. DOI: 10.1038/nchem.1706 (acccessed 2025/02/03/04:49:07).
(13) Benniston, A. C.; Copley, G. Lighting the way ahead with boron dipyrromethene (Bodipy) dyes. *Phys. Chem. Chem. Phys.* **2009**, *11* (21), 4124. DOI: 10.1039/b901383k (acccessed 2025/02/03/04:57:19).
(14) Ng, J. D.; Upadhyay, S. P.; Marquard, A. N.; Lupo, K. M.; Hinton, D. A.; Padilla, N. A.; Bates, D. M.; Goldsmith, R. H. Single-Molecule Investigation of Initiation Dynamics of an Organometallic Catalyst. *J. Am. Chem. Soc.* **2016**, *138* (11), 3876-3883. DOI: 10.1021/jacs.6b00357 (acccessed 2025/09/02/19:33:08).
(15) Pibiri, E.; Holzmeister, P.; Lalkens, B.; Acuna, G. P.; Tinnefeld, P. Single-Molecule Positioning in Zeromode Waveguides by DNA Origami Nanoadapters. *Nano Lett.* **2014**, *14* (6), 3499-3503. DOI: 10.1021/nl501064b (acccessed 2025/01/09/17:33:15).
(16) Levene, M. J. Zero-Mode Waveguides for Single-Molecule Analysis at High Concentrations. *Science* **2003**, *299* (5607), 682-686. DOI: 10.1126/science.1079700 (acccessed 2019/10/15/02:23:05). From DOI.org (Crossref).
(17) Sneh, O.; George, S. M. Thermal Stability of Hydroxyl Groups on a Well-Defined Silica Surface. *J. Phys. Chem.* **1995**, *99* (13), 4639-4647. DOI: 10.1021/j100013a039 (acccessed 2025/01/22/22:44:05).

(18) Coperet, C.; Chabanas, M.; Petroff Saint-Arroman, R.; Basset, J. M. Homogeneous and heterogeneous catalysis: bridging the gap through surface organometallic chemistry. *Angewandte Chemie International Edition* **2003**, *42* (2), 156-181.
(19) Vaccaro, L.; Cannas, M.; Radzig, V.; Boscaino, R. Luminescence of the surface nonbridging oxygen hole center in silica: Spectral and decay properties. *Phys. Rev. B* **2008**, *78* (7), 075421. DOI: 10.1103/PhysRevB.78.075421 (acccessed 2025/01/22/22:44:30).
(20) Lupo, K. M.; Hinton, D. A.; Ng, J. D.; Padilla, N. A.; Goldsmith, R. H. Probing Heterogeneity and Bonding at Silica Surfaces through Single-Molecule Investigation of Base-Mediated Linkage Failure. *Langmuir* **2016**, *32* (36), 9171-9179. DOI: 10.1021/acs.langmuir.6b02456 (acccessed 2025/01/15/01:33:38).
(21) Wang, W.; Vaughn, M. W. Morphology and Amine Accessibility of (3-Aminopropyl) Triethoxysilane Films on Glass Surfaces. *Scanning* **2008**, *30* (2), 65-77. DOI: 10.1002/sca.20097 (acccessed 2025/07/20/22:24:48).
(22) Sindorf, D. W.; Maciel, G. E. Carbon-13 CP/MAS NMR study of molecular motion in n-alkylsilane bonded to the silica surface. *J. Am. Chem. Soc.* **1983**, *105* (7), 1848-1851. DOI: 10.1021/ja00345a028 (acccessed 2025/07/20/22:52:56).
(23) Gangoda, M.; Gilpin, R. K.; Figueirinhas, J. Deuterium nuclear magnetic resonance studies of alkyl-modified silica. *J. Phys. Chem.* **1989**, *93* (12), 4815-4818. DOI: 10.1021/j100349a027 (acccessed 2025/07/20/23:01:29).
(24) Lu, H. P.; Xun, L. Y.; Xie, X. S. Single-molecule enzymatic dynamics. *Science* **1998**, *282* (5395), 1877-1882.
(25) English, B. P.; Min, W.; van Oijen, A. M.; Lee, K. T.; Luo, G. B.; Sun, H. Y.; Cherayil, B. J.; Kou, S. C.; Xie, X. S. Ever-fluctuating single enzyme molecules: Michaelis-Menten equation revisited. *Nat Chem Biol* **2006**, *2* (2), 87-94.
(26) Scheerer, D.; Levy, D.; Casier, R.; Riven, I.; Mazal, H.; Haran, G. Interplay between conformational dynamics and substrate binding regulates enzymatic activity: a single-molecule FRET study. *Chem Sci* **2025**, *16* (7), 3066-3077.
(27) Li, W.; Yang, M.; Zhao, Z.; Zhao, M.; Ye, R.; Fu, B.; Chen, P. Single-molecule reaction mapping uncovers diverse behaviours of electrocatalytic surface Pd–H intermediates. *Nature Catalysis* **2025**, 1-10.
(28) Devasia, D.; Wilson, A. J.; Heo, J.; Mohan, V.; Jain, P. K. A rich catalog of C–C bonded species formed in CO2 reduction on a plasmonic photocatalyst. *Nature communications* **2021**, *12* (1), 2612.
(29) Werny, M. J.; Siebers, K. B.; Friederichs, N. H.; Hendriksen, C.; Meirer, F.; Weckhuysen, B. M. Advancing the compositional analysis of olefin polymerization catalysts with high-throughput fluorescence microscopy. *J Am Chem Soc* **2022**, *144* (46), 21287-21294.
(30) Sambur, J. B.; Chen, T.-Y.; Choudhary, E.; Chen, G.; Nissen, E. J.; Thomas, E. M.; Zou, N.; Chen, P. Sub-particle reaction and photocurrent mapping to optimize catalyst-modified photoanodes. *Nature* **2016**, *530* (7588), 77-80.
(31) Mao, X.; Liu, C.; Hesari, M.; Zou, N.; Chen, P. Super-resolution imaging of non-fluorescent reactions via competition. *Nature Chemistry* **2019**, *11* (8), 687-694. DOI: 10.1038/s41557-019-0288-8 (acccessed 2025/01/15/00:56:32).
(32) Roeffaers, M. B. J.; Sels, B. F.; Uji-i, H.; De Schryver, F. C.; Jacobs, P. A.; De Vos, D. E.; Hofkens, J. Spatially resolved observation of crystal-face-dependent catalysis by single turnover counting. *Nature* **2006**, *439* (7076), 572-575.
(33) Wu, L.-W.; Lyu, P.-T.; Zhang, R.; Chen, K.; Zuo, L.; Song, D.; Feng, Z.-Z.; Nie, Y.-T.; Cheng, X.; Huang, T.-X. Nanoscale correlation of single-molecule reactivity and charge carrier dynamics in a two-dimensional layered InSe photocatalyst. *Nature Catalysis* **2026**, 1-8.
(34) Decan, M. R.; Impellizzeri, S.; Marin, M. L.; Scaiano, J. C. Copper nanoparticle heterogeneous catalytic 'click'cycloaddition confirmed by single-molecule spectroscopy. *Nature communications* **2014**, *5* (1), 4612.

(35) Shen, M.; Ding, T.; Tan, C.; Rackers, W. H.; Zhang, D.; Lew, M. D.; Sadtler, B. In situ imaging of catalytic reactions on tungsten oxide nanowires connects surface–ligand redox chemistry with photocatalytic activity. *Nano Letters* **2022**, *22* (12), 4694-4701.
(36) Dong, B.; Pei, Y.; Zhao, F.; Goh, T. W.; Qi, Z.; Xiao, C.; Chen, K.; Huang, W.; Fang, N. In situ quantitative single-molecule study of dynamic catalytic processes in nanoconfinement. *Nature Catalysis* **2018**, *1* (2), 135-140.
(37) Costa, P.; Sandrin, D.; Scaiano, J. C. Real-time fluorescence imaging of a heterogeneously catalysed Suzuki–Miyaura reaction. *Nature Catalysis* **2020**, *3* (5), 427-437.
(38) Cavell, A. C.; Jensen, E. T.; Brewster, B. A.; Wu, M.; Smith, M. A.; Mattei, M. S.; Czerwinski, R.; Kneeley, L. C.; Foy, M. S.; Fleming, A. F. Imaging of Lithium Ion Release from Individual Cathode Particles Shows Evidence for Diversity of Intraparticle Contacts. *Chem Mater* **2025**, *37* (15), 5785-5795.
(39) Reis, I. F.; Gehlen, M. H. Single-Molecule Catalysis in the Palladium Cross-Coupling Reaction Cycle. *The Journal of Physical Chemistry Letters* **2024**, *15* (9), 2352-2358.
(40) Yu, D.; Garcia IV, A.; Blum, S. A.; Welsher, K. D. Growth kinetics of single polymer particles in solution via active-feedback 3D tracking. *J Am Chem Soc* **2022**, *144* (32), 14698-14705.
(41) Garcia IV, A.; Saluga, S. J.; Dibble, D. J.; López, P. A.; Saito, N.; Blum, S. A. Does Selectivity of Molecular Catalysts Change with Time? Polymerization Imaged by Single-Molecule Spectroscopy. *Angewandte Chemie International Edition* **2021**, *60* (3), 1550-1555.
(42) Liu, C. M.; Kubo, K. R.; Wang, E. D.; Han, K. S.; Yang, F.; Chen, G. Q.; Escobedo, F. A.; Coates, G. W.; Chen, P. Single polymer growth dynamics. *Science* **2017**, *358* (6361), 352-355. DOI: 10.1126/science.aan6837.
(43) Ye, R.; Sun, X.; Mao, X.; Alfonso, F. S.; Baral, S.; Liu, C.; Coates, G. W.; Chen, P. Optical sequencing of single synthetic polymers. *Nat Chem* **2024**, *16* (2), 210-217.
(44) Rybina, A.; Lang, C.; Wirtz, M.; Grußmayer, K.; Kurz, A.; Maier, F.; Schmitt, A.; Trapp, O.; Jung, G.; Herten, D. P. Distinguishing Alternative Reaction Pathways by Single-Molecule Fluorescence Spectroscopy. *Angewandte Chemie International Edition* **2013**, *52* (24), 6322-6325. DOI: 10.1002/anie.201300100 (acccessed 2025/01/15/05:28:12). From DOI.org (Crossref).
(45) Park, M.; Ahn, Y.; Cho, J.; Jang, J.; Lee, W. J.; Seo, S.; Lee, S.; Seo, D. Real-Time Visualisation of Reaction Kinetics and Dynamics: Single-Molecule Insights into the Iminium-Catalysed Diels–Alder Reaction. *Angewandte Chemie* **2025**, *137* (45), e202506535.
(46) Yang, C.; Li, Y.; Zhou, S.; Guo, Y.; Jia, C.; Liu, Z.; Houk, K. N.; Dubi, Y.; Guo, X. Real-time monitoring of reaction stereochemistry through single-molecule observations of chirality-induced spin selectivity. *Nature Chemistry* **2023**, *15* (7), 972-979.
(47) Allen, D. P.; Van Wingerden, M. M.; Grubbs, R. H. Well-Defined Silica-Supported Olefin Metathesis Catalysts. *Org. Lett.* **2009**, *11* (6), 1261-1264. DOI: 10.1021/ol9000153 (acccessed 2024/12/14/17:43:18).
